# Elucidating the Transition Kernel and Anharmonic Coupling in the Spin‐crossover Process of a [Fe^III^(qsal)_2_] CH_3_OSO_3_ Complex

**DOI:** 10.1002/anie.1079807

**Published:** 2026-04-01

**Authors:** Soumyajit Mitra, Dilara Farkhutdinova, Sebastian Mai, Stuart A. Hayes, Yifeng Jiang, Tadahiko Ishikawa, Kazuyuki Takahashi, Leticia González, R. J. Dwayne Miller

**Affiliations:** ^1^ Departments of Chemistry and Physics University of Toronto Toronto Canada; ^2^ Institute of Theoretical Chemistry Faculty of Chemistry University of Vienna Vienna Austria; ^3^ Vienna Doctoral School in Chemistry (DoSChem) University of Vienna Vienna Austria; ^4^ European XFEL Schenefeld Germany; ^5^ State Key Laboratory of Precision Spectroscopy East China Normal University Shanghai China; ^6^ Department of Chemistry Institute of Science Tokyo Tokyo Japan; ^7^ Department of Chemistry Kobe University Kobe Japan

**Keywords:** anharmonicity, multireference, nonadiabatic, non‐impulsive, spin‐crossover

## Abstract

A spin‐crossover (SCO) process involves a change in the spin‐state, affecting the spatial distribution of electron density through spin‐orbit coupling. SCO can be understood as the interplay of anharmonically coupled vibrational modes that collectively drive the system across curve‐crossings. However, these modes are difficult to identify due to challenges in simulating open‐shell systems. Here, we combine ultrafast broadband transient absorption spectroscopy in single crystals with multireference excited‐state dynamical simulations to reveal the SCO mechanism in an Fe(III) complex. We identify the key doorway modes that direct the system across the curve‐crossing region to form the high‐spin state. The pronounced anharmonicity and reactive forces at SCO curve crossings provide a strong driving force for these displaced modes, leading to phase‐delayed, coherent non‐impulsive vibrational energy transfer. This study leads to unprecedented direct visualization of the SCO dynamics, revealing how the transition kernel and low‐dimensional pathways emerge from the strongly anharmonic crossing regions of the potential energy surfaces. A detailed understanding of these SCO processes is crucial for the development of advanced materials with applications ranging from high‐speed memory storage to light‐harvesting devices.

## Graphical abstract

Ultrafast broadband transient absorption spectroscopy and multireference excited‐state nonadiabatic calculations in an open‐shell Fe(III) complex unveil the rich electronic and vibrational dynamics detailing the key reactive modes driving the spin‐crossover process. This work uncovers a new coherent vibrational energy transfer mechanism, highlighting the anharmonic nature of the potential energy surface.

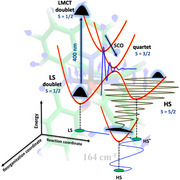

## 1 Introduction

Curve‐crossing events often define photochemical reactivity, with anharmonic couplings directing the correlated motions that transform reactants into products [1, 2]. At such reactive crossings or transition states, the effective dimensionality is reduced, and only a few key vibrational coordinates strongly couple to the reaction, forming a transition kernel that makes chemical reactivity a transferable concept. However, identifying these decisive modes is challenging, since vibrational motions mix along the reaction path and rarely align with simple normal modes. A particularly instructive case is the change in spin‐state—known as a spin‐crossover (SCO) process—which reshapes the spatial distribution of electron density in close analogy to an intramolecular charge‐transfer event. This behavior reflects the fermionic requirement of Hund's rule, which governs the energy ordering of spin levels. The resulting redistribution of electrons couples to nuclear motion, guiding the molecule toward a new equilibrium geometry associated with the spin change [3, 4, 5, 6]. The dynamics of SCO are influenced by spin‐orbit coupling and Franck‐Condon (FC) factors, which define the doorway between the low‐spin (LS) and the high‐spin (HS) states. Observed primarily in transition metal complexes, SCO is central for photochromism, memory storage, emerging quantum technologies, and light‐harvesting materials with long‐lived photoexcited states [7, 8, 9, 10, 11, 12, 13, 14].

Prototypical examples of photoinduced SCO are found in Fe(II) systems, where the transition from the LS to the HS state is accompanied by an elongation (0.2 Å) of the Fe─N bond [15, 16, 17, 18, 19, 20], the dominant nuclear motion driving the process. This bond elongation, however, is coupled to motions along other coordinates, reflecting the complexity of the SCO process. In the well‐studied [Fe^II^(bpy)_3_]^2+^ complex (bpy = 2,2′‐bipyridine), the HS state has been argued to be structurally trapped by the activation and damping of the molecular breathing of the FeN_6_ octahedron, resulting in an increase of the Fe─N bond length during the SCO process [21]. Studies have also shown that the activation of the breathing mode increases the N─Fe─N bond angle, which impulsively excites the bending modes or the torsional coordinates [22, 23]. A more detailed description of the SCO mechanism has emerged from ultrafast electron diffraction experiments that highlighted the presence of ligand sphere motion alongside the Fe─N bond elongation and N─Fe─N bond angle increase as the key reactive modes that trigger the SCO process [6, 24]. The general picture involving the electronic evolution after photoexcitation to the singlet metal‐to‐ligand charge transfer (^1^MLCT) state has underscored the existence of intermediate ^1,3^MLCT or ^3^T states during the photoinduced SCO process, where a sequential population transfer occurs in the following manner: ^1^MLCT→^3^MLCT/^3^T→HS (^5^T_2_) [10, 11, 18, 25, 26, 27, 28]. However, an optical pump‐probe study with better temporal resolution has inferred a sub‐50 fs formation of the HS (^5^T_2_) state in the Fe(II) complex without the involvement of the intermediate state [29]. The photoinduced generation of the HS state can also be described as light‐induced excited state spin trapping (LIESST) since the HS state is a metastable state that returns to the LS ground state, which is contrary to the thermodynamically stable HS state formed with increasing temperature [20, 30]. While several Fe(II) complexes have been studied extensively, far less is known about SCO in Fe(III) counterparts [31]. Existing ultrafast studies of Fe(III) are mostly limited to spectroscopic measurements on solid crystalline or nanocrystalline samples, revealing SCO dynamics on a sub‐200 fs timescale [32, 33, 34, 35, 36]. The limitation arises largely because the excited‐state lifetimes of Fe(III) species in solution are often drastically shortened by solvent‐mediated non‐radiative decay pathways, fast recombination of the initial charge transfer excited states, and vibrational quenching, which are suppressed in the rigid crystalline environment [37, 38, 39, 40]. The crystalline state provides opportunities to carry out reactions in a solvent‐free environment and promotes specific system‐bath interactions due to the spatial arrangement of the reactants in the lattice [41, 42, 43, 44, 45, 46]. This offers the ability to control, to a certain degree, the anharmonic coupling in the nuclear reorganization during the SCO process. The previous studies on solid‐state Fe(III) SCO have further suggested the presence of an intermediate state that mediates the conversion from the initially photoexcited ligand‐to‐metal charge transfer (LMCT) LS (*S* = 1/2) state to the HS (*S* = 5/2) state [32, 33, 34, 35, 36]. On the computational side, quantum chemical calculations on Fe(III) complexes remain equally scarce [47], since treating open‐shell species requires expensive multireference methods, making excited‐state dynamics simulations particularly challenging.

**FIGURE 1:**
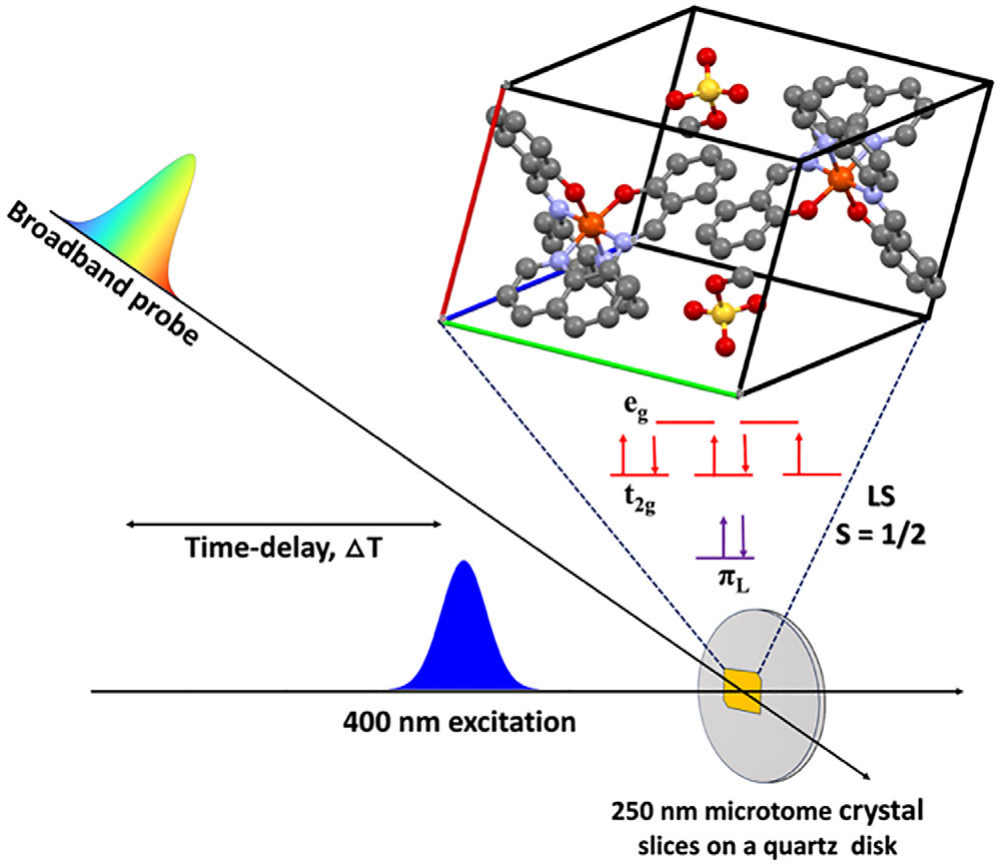
Broadband transient absorption spectroscopy in a single crystal [Fe^III^(qsal)_2_] CH_3_OSO_3_ complex. Crystal structure based on data reported in Ref. [51]. The iron (Fe) atoms are colored orange, the nitrogen atoms are colored blue, the oxygen atoms are colored red, the sulfur atoms are colored yellow, the carbon atoms are colored gray, and the hydrogen atoms are omitted for visual clarity in the crystal lattice. The parallelepiped shape represents a unit cell, and the red, green, and blue lines are the crystallographic *a*, *b*, and *c* axes, respectively. The Fe(III) complex is in the LS state at room temperature with one unpaired *d* electron in the metal orbitals.

In this study, we uncover the ultrafast SCO mechanism in a single crystal of [Fe^III^(qsal)_2_] CH_3_OSO_3_ (Hqsal = *N*‐(8‐quinolyl)salicylaldimine) complex using broadband ultrafast transient absorption (TA) spectroscopy (Figure 1). By combining time‐resolved experiments with nonadiabatic dynamical simulations and advanced data‐analysis techniques, we disentangle the cascade of events that drive the system from the initially excited LMCT doublet state (*S* = 1/2) to the HS sextet state (*S* = 5/2). Central to our theoretical strategy is the application of multiconfigurational quantum chemistry methods. By explicitly constructing wavefunctions that account for all chemically relevant electronic configurations on an equal footing, this framework overcomes the limitations of single‐reference methods and achieves the accuracy required to rigorously map the complex electronic cascade in the SCO mechanism of the [Fe^III^(qsal)_2_] CH_3_OSO_3_ complex. In this pursuit, we identified the transition kernel of the SCO process, in which a butterfly‐like motion of the ligands alters the N─Fe─N bond angle, serving as the key reaction coordinate for the SCO curve‐crossing event. Additionally, serendipitous observation of the non‐impulsive growth of oscillation amplitude during the relaxation process in the HS potential reflects both the anharmonic nature of the SCO process and the system's out‐of‐equilibrium position near the curve‐crossing region during intersystem crossing (ISC) [48, 49, 50]. This highlights how the spatial arrangement of molecules in the crystal lattice modulates the anharmonicity of the many‐body potential in the curve‐crossing region. The remarkable agreement between the experimental data and the simulations validates the proposed mechanism and provides unprecedented molecular‐level insight into the nuclear motions governing Fe(III) spin‐state switching in single crystals.

## 2 Results

### 2.1 Electronic Dynamics of [Fe^III^(qsal)_2_] CH_3_OSO_3_ in Single Crystals

**FIGURE 2:**
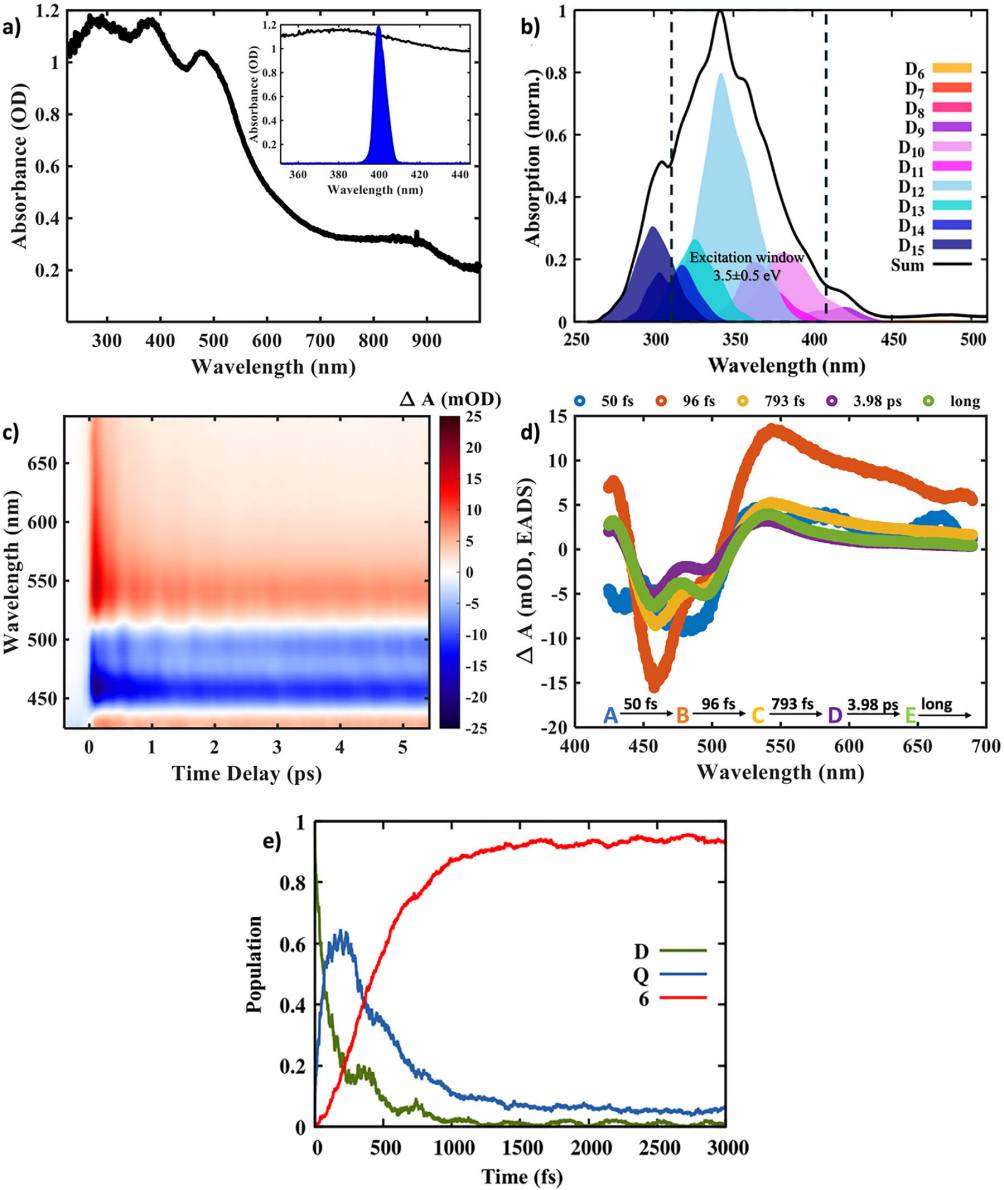
Spin‐state dynamics in the Fe(III) system from spectroscopy and computational data. (a) Steady‐state absorption spectrum of [Fe^III^(qsal)_2_] CH_3_OSO_3_ complex at 297 K (LS state). Inset: Laser spectrum of 400 nm. (b) Calculated absorption spectrum of the isolated [Fe^III^(qsal)_2_]^+^ cation, deconvoluted according to the contributions of the involved electronic doublet spin‐states. (c) Broadband ultrafast TA data of the [Fe^III^(qsal)_2_] CH_3_OSO_3_ complex photoexcited with a 400 nm actinic pump pulse. (d) Evolution Associated Difference Spectra (EADS) were obtained after the sequential global analysis of the TA data shown in (c). (e) Calculated time‐resolved populations from the doublet (D) state to the intermediate quartet (Q) state in 85 fs, followed by the formation of the HS sextet (6) state in 370 fs. For simplicity, we present the total electronic populations grouped by spin multiplicity, averaged over 842 multireference nonadiabatic dynamic trajectories spanning 142 electronic/spin states. The evolution of the individual electronic state populations (see Figure S26) reveals that the dynamics of the doublet and quartet states involve multiple states and a high density of crossings, rather than a single crossing. In contrast, the spectroscopic measurements provide a more coarse‐grained view, reflecting the ensemble‐averaged dynamics and spectral changes.

Figure 2a shows the absorption spectrum of the LS state of a single crystal [Fe^III^(qsal)_2_] CH_3_OSO_3_ complex at room temperature [51]. The calculated absorption spectrum of the isolated [Fe^III^(qsal)_2_]^+^ cation in the gas phase is shown in Figure 2b. The experimental absorption spectrum is broadened and red‐shifted relative to the isolated molecular spectrum due to intermolecular interactions. The maximum of the calculated absorption spectrum, obtained at the equilibrium geometry of the electronic ground state (D_0_), is dominated by the LMCT transition leading to the excited D_12_ state (see Supporting Information Section S1 and Table S1). Figure 2c shows the temporal evolution of the TA data at room temperature after LMCT photoexcitation (inset of Figure 2a) at 400 nm (see Supporting Information Section S2 for experimental details).

We modeled our TA data using a sequential global analysis method [41, 52] (Supporting Information Section S3.1). Figure 2d shows the evolution‐associated difference spectra (EADS) for individual species with relaxation time constants of 50 ± 2, 96 ± 3, 793 ± 8 fs, and 3.98 ± 0.37 ps, along with a non‐decaying long component (see Figure S6 for the fitting agreement). From the global analysis, we infer the instrument response function limited 50 fs component as the evolution of the system from the FC region of the LMCT doublet state (species A) to an intermediate state (species B). This is followed by a 96 fs dynamics, during which the system evolves into species C from species B. Experimental TA data complemented by the nonadiabatic dynamic simulations of the [Fe^III^(qsal)_2_]^+^ cation illustrate that the SCO process begins in the doublet LMCT states, proceeds through the quartet intermediate states, and culminates in the formation of the sextet states within <500 fs, as evidenced in Figure 2e. By combining the nonadiabatic dynamic simulations with the global analysis procedure, we assign species B as the intermediate quartet (*S* = 3/2) state and species C as the HS sextet (*S* = 5/2) state. The excellent agreement between the simulated and observed experimental timescales (Table 1) validates the simulations. Analysis of the simulations reveals that the SCO dynamics is dominated by changes in spin density localized to the molecular frame, arising from the rapid decoherence of the spatially extended delocalized electronic states [53] to single Fe(III) sites in the crystal. A previous report on a Fe(III) complex has shown sub‐100 fs LMCT dynamics followed by a 160 fs ISC timescale for the sextet HS state formation [36], supporting the above assignments. The presence of a sub‐picosecond relaxation component (793 fs) in the EADS analysis corresponds to the structural reorganization of the system in the sextet HS potential energy surface (PES) to reach the minimum of the HS geometry [36] (species D; see Figure S5). The additional ∼4 ps component corresponds to the vibrational cooling of the HS structure and energy transfer to the surrounding medium or lattice [54, 55]. When we analyzed the long‐time data, we identified a 30 ps Decay‐Associated Difference Spectra (DADS) component (Figure S7a,b). This observation is indicative of the cooperative effect of heat dissipation in the [Fe^III^(qsal)_2_] CH_3_OSO_3_ crystal, resulting in lattice expansion [54, 56]. The relaxation of the photoexcited SCO system to the initial LS ground state in crystals occurs on a few µs timescale, which is beyond the time range (1 ns; Figure S7c) of our measurement [56, 57] (see Supporting Information Section S3.2 for long‐time dynamics). The ultrafast (<100 fs) formation of the HS state and its long‐lived nature give a rare example of LIESST in [Fe^III^(qsal)_2_] CH_3_OSO_3_ derivative [51]. Although the Fe(III) compounds were believed to be unable to undergo LIESST because of smaller structural changes (ΔFe─N = 0.1–0.13 Å) as compared to Fe(II) (ΔFe─N = 0.2 Å) derivatives [31], the discovery of [Fe^III^(qsal)_2_] CH_3_OSO_3_ derivative showing ultrafast SCO with large structural changes (ΔFe─N ∼ 0.19 Å) [51] highlights the high quantum yield of the process. This is similar to Fe(II) derivatives with a unity quantum yield [29].

**TABLE 1:** Theoretical analysis of key time constants and normal modes.

Source	Time constants (fs) from the exponential (theory) and global (experimental) fit	Frequencies (cm^−1^)
**Theory**: Time‐resolved electronic populations from nonadiabatic dynamic simulation (Supporting Information Section S5)	85 (doublet to quartet), 370 (quartet to sextet)	23, 40, 52, 75, 81, 96, 108
**Theory**: Potential energy surface relaxation over time (Figure 5)	348 (relaxation time)	196, 1720
**Theory**: Simulated TAS (Figure S29 and Supporting Information Section S6)	444 (HS state formation)	23, 40, 52, 75, 96, 108, 164, 196, 238
**Theory**: Coherent normal mode analysis (Supporting Information Section S7)	—	23, 40, 52, 80, 96, 164, 201, 229, 242, 257, 273, 1720
**Experiment**: TAS in Figure 2	50 (doublet to quartet), 96 (quartet to sextet), 793 (structural reorganization)	23, 42, 53, 65, 84, 96, 104, 117, 125, 138, 160, 194, 236, 257, 265

The source plots, exponential fits, and corresponding Fourier transform spectra are provided in the Supporting Information Sections S5, S6, and S7. Comparison with experimental findings is shown in the fifth row.

### 2.2 Vibrational Dynamics of [Fe^III^(qsal)_2_] CH_3_OSO_3_

**FIGURE 3:**
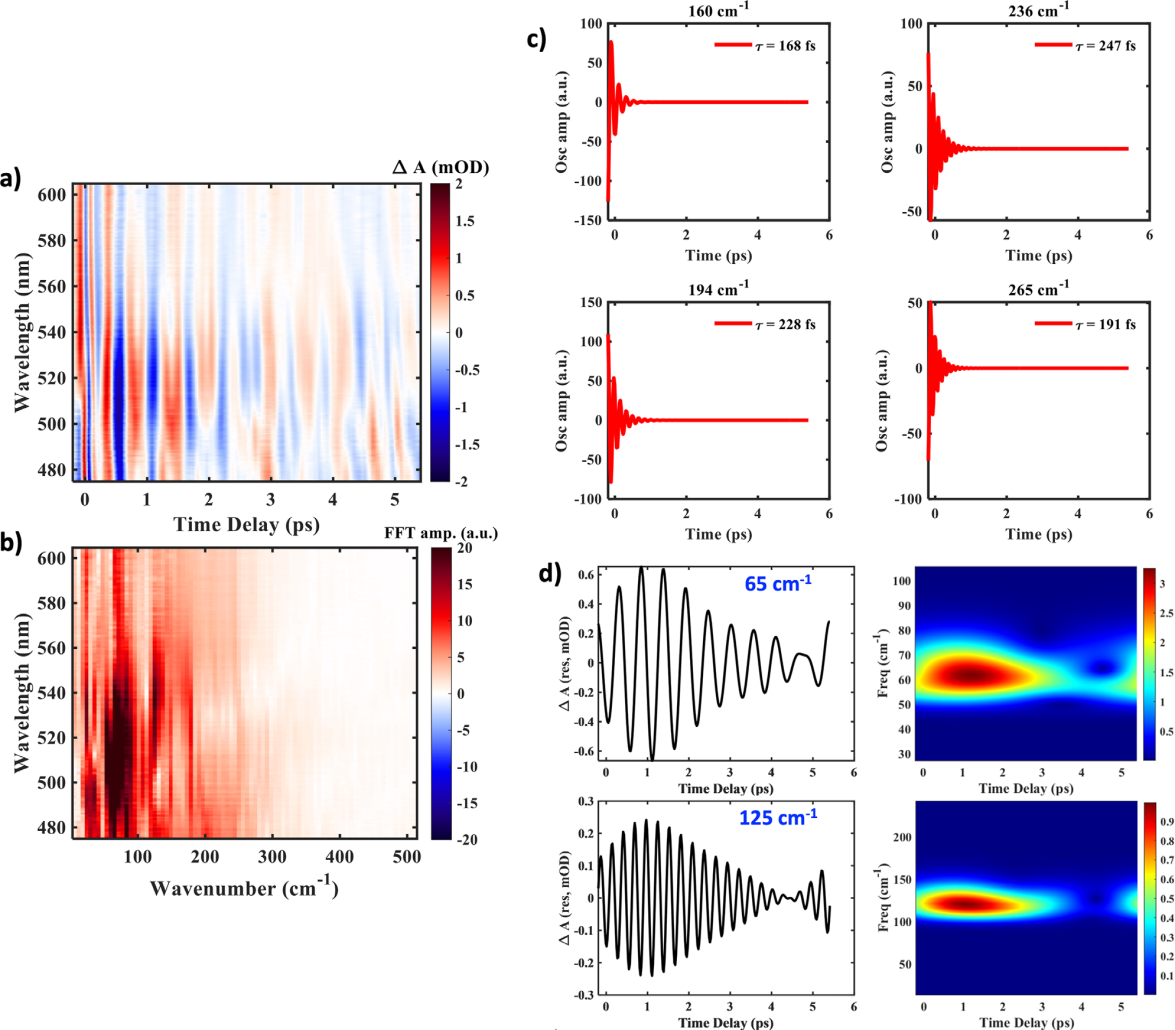
Vibrational analysis of wavepacket motion in the Fe(III) system. (a) Residual map obtained after subtracting the global fit from the TA data. The plot represents coherent oscillations correlated to the wavepacket dynamics. (b) Fast Fourier transform (FFT) of the residual matrix along the time axis. (c) The observed modes decaying in <250 fs strongly correlate with the SCO dynamics. The vibrational components were obtained through time‐frequency and Fourier‐filtering analysis methods, as described in Supporting Information Section S3.3. (d) Non‐impulsive generation of 65 cm^−1^ (upper‐left panel) and 125 cm^−1^ (lower‐left panel) modes, displaying a growth in the amplitude of oscillations with time. These frequencies are Fourier filtered in the frequency domain. The upper‐right and lower‐right panels display the wavelet analysis of 65 and 125 cm^−1^ modes, respectively. The wavelet analysis of the unfiltered residual also shows the growth of the above‐mentioned modes (Figure S21).

Figure 3a,b shows the coherent vibrational dynamics after subtracting the global analysis fit from the raw data [58, 59] (Figure 3a: residual contour map and Figure 3b: FFT contour map). To identify the nature of the vibrational modes, we applied time‐frequency analysis and Fourier‐filtering methods to isolate the frequency component of interest [41, 42, 60, 61] (Supporting Information Section S3.3 and Figure S8). We have identified four vibrational modes that are decaying on 160–250 fs timescales, and are sufficiently close to the SCO process to be assigned as reactive modes that define the epicenter of the reaction forces [62] (Supporting Information Section S3.4). These are 160 cm^−1^ (*τ_d_*/*τ_p_* = 0.80), 194 cm^−1^ (*τ_d_*/*τ_p_* = 1.32), 236 cm^−1 ^(*τ_d_*/*τ_p_* = 1.75), and 265 cm^−1^ (*τ_d_*/*τ_p_* = 1.52), respectively (see Figure 3c; *τ_d_*/*τ_p_* is the ratio of decay time, *τ_d_*, to period of oscillation, *τ_p_*). Since the 160 cm^−1^ mode is decaying at a faster timescale than its period of oscillation, it is critically damped. Thus, there would be less probability of recurrence along this coordinate back to the initial state, making it the strongest contributor to the reaction coordinate. This implies that the reaction forces along this coordinate dominate the structural relaxation as the system passes through the crossing region to form the HS state during the SCO process, with its assignment as the key reactive mode.

In addition to the modes strongly coupled to the reaction coordinate, we find molecular vibrations decaying on a sub‐picosecond timescale, as listed in Figure S20. Since their decay timescale closely matches the reorganization timescale (793 fs) from the global analysis of the TA data, we assign these vibrations as assistive modes involved in molecular reorganization of the HS structure during the ultrafast SCO process. The above correlations were made possible by the constrained single‐crystal environment, which ensures well‐defined initial conditions in the ultrafast SCO process (HS state formation) within a uniquely defined lattice structure, allowing us to track the structural relaxation along specific modes that collectively define the reorganization coordinate [41]. These details would be lost in the random distributions for solution‐state studies [41]. Since the HS structure is not fully relaxed to the global minimum of the product surface during the ultrafast SCO curve‐crossing event, it results in a non‐equilibrium geometry in the HS PES, leading to structural relaxation along the reorganization coordinate.

Upon closer inspection of the vibrational dynamics, we observe an unusual temporal behavior with the 65 and 125 cm^−1^ modes (Figure 3d). Unlike other oscillations, the amplitude of these modes increases over time. This is counterintuitive because, in an impulsive excitation, all the vibrations are excited simultaneously at *t* = 0 with displacive excitation in the excited‐state surface, forming a wavepacket. The delayed response of the vibrational modes indicates that these modes are not excited by the actinic pump and are non‐impulsive. Since the amplitude of these modes reaches its maximum value in a picosecond, they are strongly correlated to the structural reorganization in the HS state. Figure S22a,b shows the contour map for the filtered frequencies at 65 and 125 cm^−1^, respectively. It clearly shows that the growth is observable in the 490–550 nm wavelength range, affirming that this wavelength range is sensitive to the SCO reaction, leading to the formation of the HS state. Interestingly, we have also seen the decay of the assistive modes on a sub‐picosecond timescale (Figure S20), suggesting a possible correlation between the assistive modes and the vibrations whose amplitudes are increasing, resulting in a non‐ubiquitous vibrational energy transfer mechanism [41] (see Supporting Information Section S4, Figures S23, and S24). This observation is intriguing as the initial ultrafast SCO process evolves the system to a highly anharmonic region of the HS PES, driving the structural transition. The persistence of these non‐impulsively generated modes beyond the subpicosecond timescale, which is normally associated with intramolecular vibrational relaxation in condensed matter systems [63], reflects that the assistive modes are more strongly coupled to each other, generating the non‐impulsive modes (Figure 3d), than to competing channels for energy relaxation in the crystalline environment.

**FIGURE 4:**
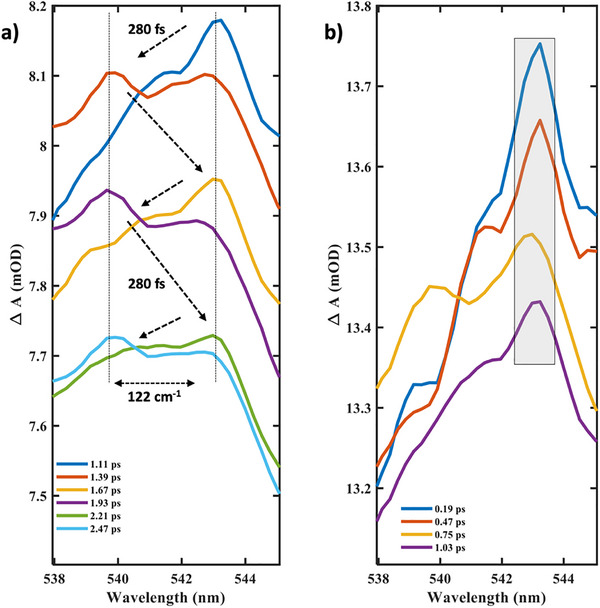
Spectroscopic signature of anharmonicity. (a) Peak shift dynamics showing the modulation of the ESA peak with 61 cm^−1^ frequency (half period = 280 fs), and an energy gap of 122 cm^−1^. (b) Peak shift dynamics begin 1 ps after time zero, highlighting the phase‐delayed non‐impulsive nature of the 61 and 122 cm^−1^ modes.

On plotting the peak maximum shift of the excited‐state absorption (ESA) signal, shown in Figure 4a, we find a strong spectral modulation with a frequency of 61 cm^−1^ (half period of 280 fs), which results in the ESA peak shifting back and forth within an energy gap of 122 cm^−1^ matching with the non‐impulsive modes discussed above (see Figure 3d). This modulation begins after 1 ps (see Figure S25), before which the peak maximum does not shift, as shown in Figure 4b. A similar 1 ps delay was found in the rise of the amplitude of 65 and 125 cm^−1^ modes, respectively (Figure 3d), suggesting a strong coupling between the non‐impulsively generated modes and the HS electronic state through an electron‐phonon or vibronic coupling mechanism. The spectral range of the peak shift dynamics (Figure 4) matches the wavelength range of the non‐impulsive modes (see Figure S22), substantiating the above correlations. This observation further highlights the impact of far‐from‐equilibrium geometry resulting from the abrupt shift in equilibrium position and the anharmonicity of the HS PES involved in the ultrafast SCO reaction.

### 2.3 Nonadiabatic Dynamics and Normal‐Mode Analysis of [Fe^III^(qsal)_2_]^+^ Cation

**FIGURE 5:**
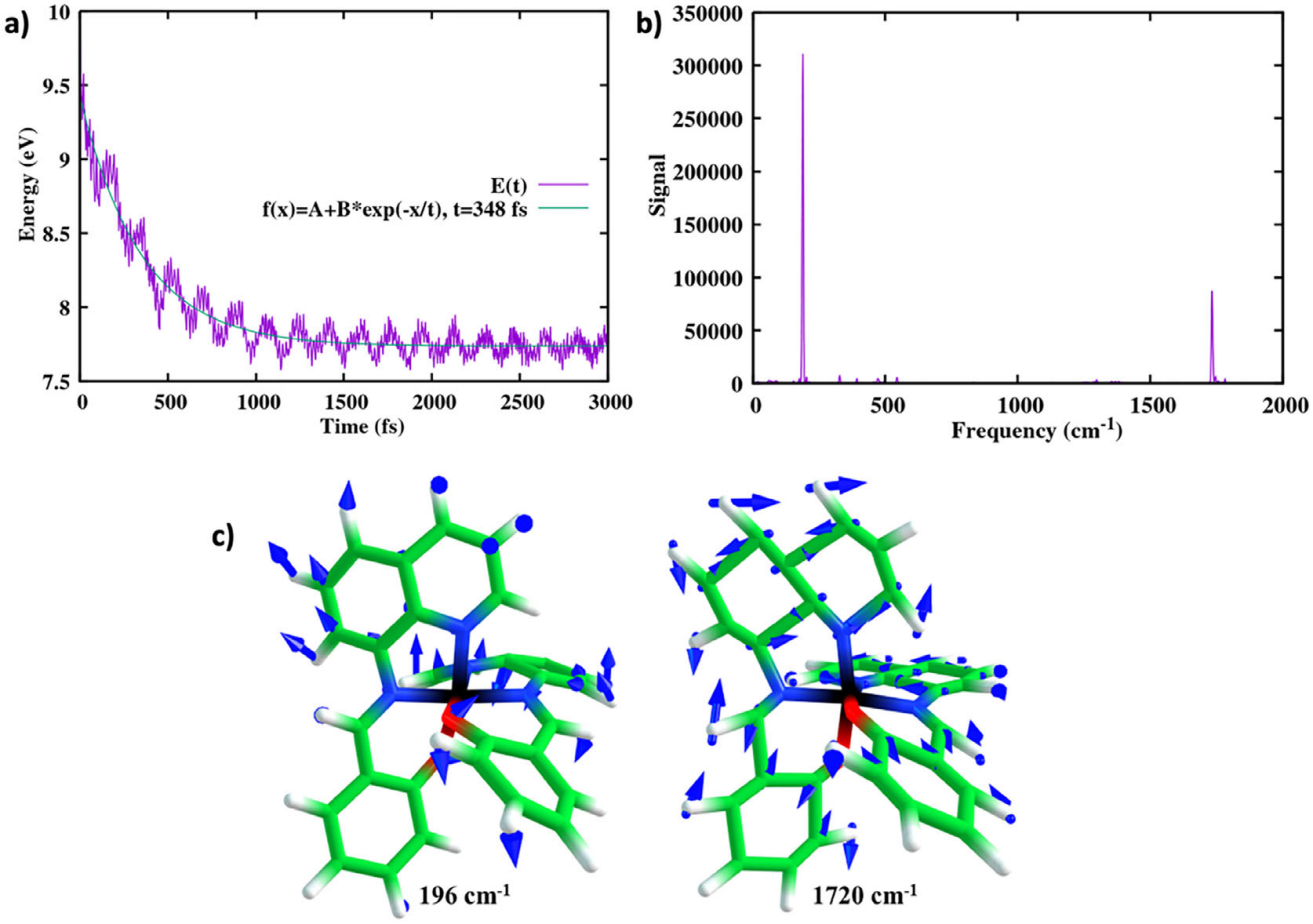
Potential energy surface relaxation over time. (a) Simulated potential energy evolution over time, overlayed with an exponential fit. (b) FFT of the oscillatory signal. The FFT analysis reveals frequency components at 196 and 1720 cm^−^^1^, respectively, which correspond well with experimentally observed reactive vibrational modes (see Figure 3c for the 194 cm^−1^ experimentally observed reactive mode). These modes likely play a key role in energy redistribution and facilitate the system's transition between the electronic states from the FC region. (c) Normal modes are identified from the Fourier transformation of the potential energy over time.

To rationalize the SCO process, we carried out nonadiabatic dynamics simulations (Supporting Information Section S5) based on a full‐dimensional vibronic model comprising 16 doublet states, 17 quartet states, and 7 sextet states. The potentials and couplings are calculated using multiconfigurational quantum chemistry, specifically the restricted active space self‐consistent field (RASSCF) [64] method (see details in methods below and in Section S1), which has been interfaced within our trajectory surface‐hopping excited‐state dynamics code SHARC [65, 66]. From the trajectories and using the descriptors and observables listed in Table 1, we identified the reactive modes among the 177 degrees of freedom, thereby capturing the interplay between electronic and nuclear degrees of freedom in the curve‐crossing regions. The simulated time‐resolved electronic populations of the doublet and the quartet states reveal vibrational modes (first row of Table 1) that drive oscillatory population exchange, sustaining coherence until the system stabilizes in the quartet state before ultimately transitioning to the sextet state. The theoretical analysis of the potential energy over time yields a relaxation time constant of 348 fs from the FC state (Figure 5a). Strong modulation in the potential energy, with two prominent frequencies of 196 and 1720 cm^−^^1^, respectively, is evident from Figure 5b. Notably, the 196 cm^−^^1^ mode closely matches the experimentally observed 194 cm^−^^1^ reactive mode (Figure 3c). Analysis of the simulated TA data for the isolated [Fe^III^(qsal)_2_]^+^ cation, particularly the ESA feature above 500 nm, indicates that the HS state forms within 444 fs (Figure S29a,b). This timescale is consistent with both the PES time evolution (see Table 1) and the ISC to the HS state (Figure 2e). The simulated TA data also exhibits coherent oscillations, with FFT analysis shown in Figure S29c. The excellent agreement between theory and experiment is summarized in Table S3. The analysis highlights maximum activity in the 196 cm^−^^1^ mode, alongside significant contributions from the 164 and 238 cm^−^^1^ frequencies (Figure S30).

The coherent mode analysis (see Supporting Information Section S7) identifies all the normal modes that are coherently or impulsively excited, regardless of their direct relevance to the SCO process (Figures S31 and S32). The mode at 164 cm^−^^1^ (experimental: 160 cm^−^^1^) corresponds to ligand butterfly motion altering the N─Fe─N bond angle (Video S1). The 238 cm^−^^1^ mode (experimental: 236 cm^−^^1^) involves the stretching of the Fe─N bond coupled with ligand torsion motion (Video S2), while the 273 cm^−^^1^ mode (experimental: 265 cm^−^^1^) represents the breathing of the FeN_4_O_2_ core unit (Video S3). The 1720 cm^−^^1^ mode corresponds to the C═C stretching of the ligands affecting the N─Fe─N bond angle (Video S4), and the 196 cm^−^^1^ mode (experimental: 194 cm^−^^1^) induces bond angle distortion in the N─Fe─N unit (Video S5). These vibrational motions, particularly the 1720 and 194 cm^−^^1^ modes (Figure 5c), drive the transition from the FC region to the SCO crossing point, and the 160 cm^−^^1^ mode facilitates the crossing, leading to the HS state formation. These three modes are collectively responsible for changing the N─Fe─N bond angle, making it a key reactive motion for the SCO process. The FC weighted factor between the photoexcited LS and the HS surface is defined along the high‐frequency (C═C stretching) 1720 cm^−1^ coordinate, enabling a <100 fs SCO process with a ∼1.5 eV driving force (Figure 5a), similar to an intramolecular electron‐transfer reaction [67]. The strong agreement between theoretical predictions and experimental data confirms the assignment of the coherent oscillations to specific vibrational modes.

## 3 Discussion

**FIGURE 6:**
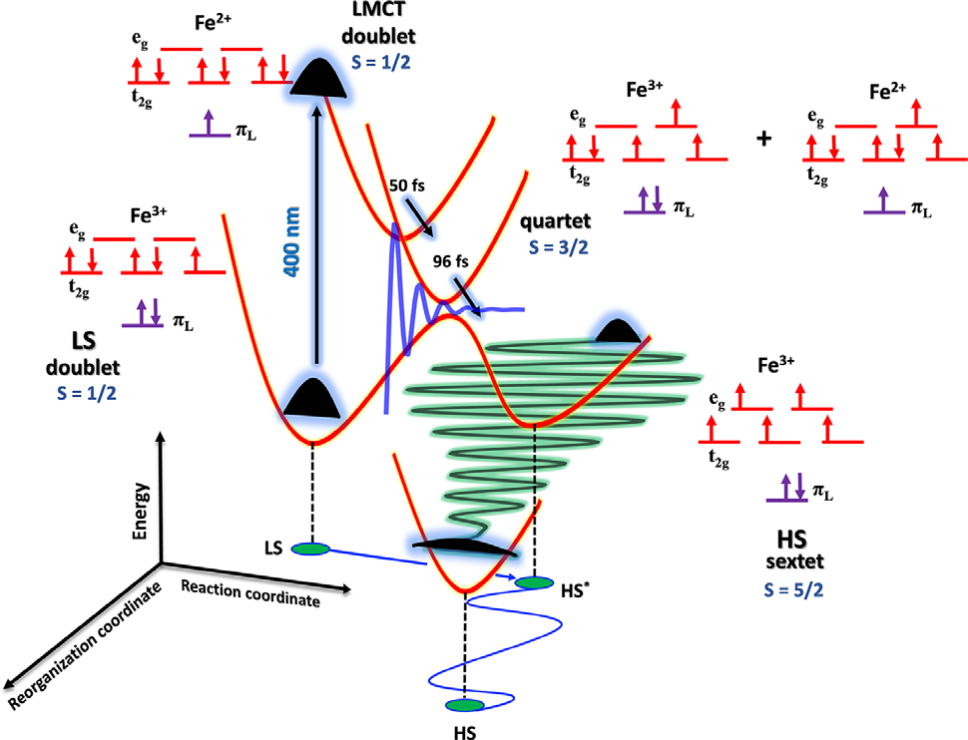
Summary of the SCO dynamics in [Fe^III^(qsal)_2_] CH_3_OSO_3_ complex with electronic distribution in the metal (*e_g_*, *t_2g_*) and the ligand (π_L_) orbitals of [Fe^III^(qsal)_2_]^+^ cation at different spin and oxidation states. The experimental timescales are shown in the figure. The theoretical timescales involved in the spin‐state dynamics are collected in Table 1. The butterfly motion of the 164 cm^−1^ mode (experimental: 160 cm^−1^) obtained from the normal mode analysis is the key reactive mode, critically damped (blue oscillation), that directs the system across the curve‐crossing region during the SCO process. The process involves: doublet LS (Fe^3+^) + hν (400 nm)→LMCT doublet (Fe^2+^)→quartet (Fe^3+^/Fe^2+^)→HS* (Fe^3+^)→HS (Fe^3+^). The ultrafast SCO process triggers the non‐impulsive generation of molecular modes (green oscillation) during structural reorganization in the HS state (HS* (Fe^3+^)→HS (Fe^3+^)). This structural reorganization is mediated by the anharmonicity of the HS PES, resulting in the nonlinear coupling of intramolecular vibrational or assistive modes (Supporting Information Section S4).

Our simulations and experiments show that the SCO process proceeds through a stepwise mechanism, evolving through an intermediate quartet spin state to reach the final HS limit. From the LS and HS crystal structures of [Fe^III^(qsal)_2_] CH_3_OSO_3_ complex [51], the average Fe─N bond length is found to increase by ∼0.19 Å during the SCO process. This elongation arises from the antibonding character of the e_g_ orbitals, which become progressively populated as the system evolves from the LS to the HS state [51, 68]. Compared with Fe(II) complexes, the Fe‐N bond length changes by ∼0.2 Å [31]. As the [Fe^III^(qsal)_2_]^+^ system transitions through quartet and sextet states, the increasing, e_g_ orbital occupancy (one electron in the quartet, two in the sextet) drives expansion and twisting of the Fe coordination shell. Thus, from the initial doublet ground state, and upon photoexcitation to the doublet LMCT state, the orbital occupancy of the electrons changes at the Fe center, triggering structural reorganization (Figure 6). The bond length analysis from the nonadiabatic dynamics simulations (see Supporting Information Section S8) shows that the Fe─N bond length expands by approximately 0.2 Å, shifting from an initial value of ∼1.96 Å to a new equilibrium around 2.20 Å within the first 100 fs. This rapid displacement is consistent with the fast kinetic component (*τ* = 85 fs) derived from the population dynamics (Table 1), which shows that the kinetics of this electronic cascade are intimately coupled to the structural timescales. The sudden electronic force creates a coherent nuclear wavepacket, manifesting as the pronounced oscillations in bond length observed in the trajectory analysis (Figure S33). This reorganization is dominated by the overdamped butterfly motion at 160 cm^−1^, which effectively dissipates the excess energy while guiding the system toward the relaxed HS geometry. Its critically damped character (with a 1/e decay faster than its period of motion) suggests strong reaction forces along this coordinate, effectively steering the system across the crossing region to form the HS sextet state. The resulting N─Fe─N distortions associated with the 160 cm^−1^ mode reflect the structural adjustments required by Hund's rule, redistributing electron density [3, 4, 5, 6, 29] as the spin multiplicity changes in the HS state (Δ*S* = 2). The angular dependence of this butterfly mode further underscores how channeling angular momentum facilitates the spin‐state transition. Recent work by Oppermann et al. has also highlighted the role of symmetry‐breaking non‐radial mode in the SCO dynamics of an Fe(II) complex [69]. In addition, we find that the Fe‐N stretching and ligand torsional modes (experimental: 265 and 236 cm^−1^; calculated: 273 and 238 cm^−1^) also contribute to the SCO dynamics. The experiment shows that, unlike the butterfly motion, these modes decay rapidly but remain underdamped. This behavior highlights their coupling to the intramolecular vibrational modes or reorganization coordinate. Similar relaxation pathways have been discussed by Veenendaal et al., where dephasing of the photoexcited state to a large density of phonon continuum states or assistive modes determines ultrafast ISC [70]. In our case, the rapid dephasing of the Fe─N breathing and ligand torsional modes precludes recurrence toward the initial LMCT state, thereby structurally trapping the system in the HS state. Finally, once the HS state is reached, further motion along the reaction coordinate is constrained by larger barriers to lattice rearrangement in the solid state. This trapping enables the observation of changes in vibrational coherences that report directly on the anharmonicity of the PES along the reorganization coordinate, providing mechanistic fingerprints of how electronic and nuclear factors conspire to drive the SCO reaction.

An important issue in solid‐state chemistry is the extent of the excitonic effect in photoexcitation, which leads to additional splitting of the absorption into exciton peaks and broadening of the absorption spectrum in a solid crystal (Figure 2a) compared to its isolated gaseous state (Figure 2b). The broadening effect of the absorption lineshape of the exciton transitions is dominated by the rapid decoherence of the LMCT excited state [41]. The initial absorption would be delocalized over multiple sites owing to the intermolecular interactions in a single crystal (π‐π interactions between ligands of neighboring complexes in [Fe^III^(qsal)_2_] CH_3_OSO_3_) [51]. However, the excited state undergoes fast decoherence due to random fluctuations in the surrounding sites and directed sampling of the excited state population that localizes the excited state [53, 71]. This directed sampling of the SCO dynamics would destroy the coherent coupling between adjacent sites, as energy dissipation (∼1.5 eV, Figure 5a) would well exceed excitonic coupling [41]. These processes would break the required condition of resonance for spatially extended states [72]. Thus, the bath fluctuations and more significantly, the intramolecular nuclear motion along the reaction coordinate due to the change in electron density for the SCO process would kill the spatially extended excitonic coherences, resulting in the collapse of the electronic wavefunction to a single Fe(III) molecular site in the crystal. This intrinsic rapid decoherence mitigates the effect of intermolecular interactions in the SCO dynamics. In this regard, our theoretical calculation of nonadiabatic simulations capturing the SCO dynamics in an isolated [Fe^III^(qsal)_2_]^+^ cation without accounting for the effect of lattice or counteranion, beautifully captures the experimental dynamics with a near‐perfect match in the structural modes directly responsible for the SCO curve‐crossing event. There is an understandable shift in the calculated spectra relative to the experiment, but it still provides an accurate assignment of electronic states. Current studies are underway to observe the effect of counteranion (CH_3_OSO_3_ vs. SCN) [51, 73] on the SCO dynamics and anharmonic coupling, using ultrafast electron diffraction measurements [1]. Counteranion effects are known to alter the macroscopic magnetic behavior of the Fe(III) complexes [31, 73].

## 4 Conclusion

SCO dynamics in transition metal complexes with open‐shell configurations remain notoriously difficult to unravel due to their multireference character and the strong coupling between spin and nuclear degrees of freedom. With few exceptions [64, 74], nonadiabatic studies on transition metal complexes have mainly addressed simpler singlet to triplet ISC processes [75], which can often be described with standard density functional theory. Here, by combining ultrafast spectroscopy and multireference RASSCF nonadiabatic dynamics, we elucidate the doublet‐quartet‐sextet SCO dynamics of the [Fe^III^(qsal)_2_] CH_3_OSO_3_ complex presented as a scheme in Figure 6. The excellent agreement between the simulated and measured spin population dynamics, together with the identification of the vibrational modes most displaced during the spin‐induced relaxation, provides unprecedented mechanistic detail of the SCO process in this Fe(III) complex, and sets a new benchmark for computational studies of spin‐state switching in open‐shell systems. The butterfly motion of the ligands, which alters the N─Fe─N bond angle, is identified as the key reaction or doorway mode guiding the system through the SCO crossing region to form the transition kernel of the ISC process. This underscores the effect of reduced dimensionality in the SCO reaction mechanism at the saddle point of the curve‐crossing region. Previous theoretical and experimental work on the Fe(II) system has highlighted the significance of the N─Fe─N bond angle in facilitating the SCO process [29, 76, 77]. In this work, supported by the theoretical calculations, we find a similar effect in the butterfly angular motion with cascaded levels of nuclear reorganization dictated by a discrete number of modes as the system relaxes further to the lowest energy HS sextet state (HS*→HS). Moreover, the rapid transformation (<100 fs) to the HS state, driven by a large energy difference (∼1.5 eV, Figure 5a), induces an abrupt shift in the equilibrium atomic coordinates. This involves significant molecular motions that push the system into a far‐from‐equilibrium region with large kinetic energy during the curve‐crossing event, unveiling the highly anharmonic region of the HS PES along the reorganization coordinate (HS*→HS, Figure 6), which ultimately leads to the coherent non‐impulsive excitation of vibrational modes. It enables spatially extended energy dissipation over the molecule and creates the ideal conditions for a nonlinear vibrational mixing process [41] (Supporting Information Section S4). These non‐impulsive modes ultimately channel the dynamics onto a few nonlinearly coupled intramolecular coordinates, resulting in the direct observation of the highly anharmonic region of the reactive crossings in the many‐body potential. The enhanced spatial correlations imposed by the solid crystalline state are evident, with discrete reactive geometries that direct the Fe(III) SCO dynamics along a well‐defined reaction pathway. This provides new strategies to probe and optimize spin‐orbit couplings and FC factors, offering a better understanding and control over molecular pathways in highly correlated SCO systems.

## 5 Methods

### 5.1 Sample Preparation

The [Fe^III^(qsal)_2_] CH_3_OSO_3_ single crystals were prepared using the methods reported previously (Ref. [51]). For the TA experiment, the single crystals were ultramicrotomed to 250 nm thickness and placed on a 1‐inch diameter quartz window (1 mm thick, see Figure 1).

### 5.2 Optical Measurement

The steady‐state absorption spectrum of the Fe(III) complex was obtained using a homemade absorption set‐up, which included a fiber‐coupled deuterium halogen lamp (DH‐2000‐BAL) and an Ocean Optics device as the detector. TA spectroscopy was performed using a home‐built TA spectrometer, as described in refs. [41] and [42]. The spectroscopy set‐up used a Ti:Sapphire laser (Coherent Legend Elite USP) with 40 fs, 800 nm output pulses running at 1 kHz. The 400 nm pulses were generated by the second harmonic generation using a BBO crystal, Barium beta Borate (*θ* = 29.3°). The 400 nm pulses ran at 125 Hz during the experiments with an excitation fluence of 1.67 mJ/cm^2^. The probe pulses were generated by focusing a portion of the 800 nm fundamental laser beam into a 2 mm path‐length quartz cuvette filled with ultrapure deionized water, which produced a supercontinuum. The supercontinuum spanned from 400 to 750 nm spectral range. The probe beam at the sample plane had a focal diameter (FWHM) of 130 µm, and the 400 nm pump beam had a focal diameter of 210 µm. The transmitted probe was routed to a home‐built Czerny‐Turner spectrograph coupled to a linear array CCD (Hamamatsu, S11155‐2048‐01 with C11165‐01 driver).

### 5.3 Computation

All simulations were performed on the isolated cation of [Fe^III^(qsal)_2_]^+^ without accounting for the effects of the counteranion or lattice.

### 5.4 Electronic Structure Methods

The doublet ground state of [Fe^III^(qsal)_2_]^+^ in the gas phase was optimized with unrestricted Kohn–Sham density functional theory using the CAM‐B3LYP functional [78] and ZORA‐def2‐SVP basis [79]. Scalar relativistic effects were included using the ZORA Hamiltonian [80]. The D3BJ correction was used to include dispersion effects [81]. For the self‐consistent‐field (SCF) calculations optimization, the resolution‐of‐identity approximation (RIJCOSX) with def2/J auxiliary basis sets and the tight SCF convergence criteria (TightSCF settings) were used [82]. A frequency calculation was conducted to compute the normal modes at this geometry and confirm its identity as a minimum through the absence of imaginary frequencies. Optimization and frequency calculation were performed using the ORCA 4.2 program package [83]. The 𝑑^5^ open‐shell electron configuration of [Fe^III^(qsal)_2_]^+^ gives rise to many close‐lying singly and doubly excited configurations that can contribute to the wave functions of excited electronic states. To describe the ground and excited electronic states of [Fe^III^(qsal)_2_]^+^ adequately, we employed multireference, restricted‐active‐space self‐consistent field (RASSCF) [64, 84] calculations with an active space that included 13 electrons in 14 orbitals (abbreviated as RASSCF(13,2,2;4,5,5)/ANO‐RCC‐VDZP), using the OpenMolcas program package version v.23.10 [85]. Further details on the electronic structure calculations are provided in Supporting Information Section S1.

### 5.5 Absorption Spectrum

A total of 1000 initial conditions (geometries and velocities), sampled from a Wigner distribution defined by a ground state frequency calculation, was used [86]. At each geometry, a single point calculation using the linear vibronic model (see below) was performed. The obtained energies and oscillator strengths were used to convolute an absorption spectrum [87] and provide initial electronic states in the energy window of 3–4 eV [88].

### 5.6 Nonadiabatic Dynamics Simulations

The nonadiabatic dynamics were started from the doublet ground state geometry. We use the trajectory surface‐hopping method SHARC [65, 89, 90] on potential energy surfaces described with a linear vibronic coupling model [66] parametrized with RASSCF. The model included 16 doublet states, 17 quartet states, and 7 sextet states. The model parameters were determined from the RASSCF (13, 2, 2; 4, 5, 5)/ANO‐RCC‐VDZP calculations. Interstate and intrastate coupling elements were computed numerically [91] using geometries displaced by ±0.05 units along each of the 177 vibrational normal modes from the doublet ground‐state geometry. RASSCF vertical excitation energies were calculated at the doublet ground‐state geometry. Spin‐orbit couplings were calculated at the doublet ground‐state geometry using the spin‐orbit‐RASSI method [92]. The nonadiabatic dynamics calculations were started from 842 trajectories generated with a Wigner ensemble. All trajectories were initialized for states within the energy window of 3–4 eV based on their oscillator strength [93]. Initial states were chosen in the adiabatic representation, leading to trajectories started in the D_3_(1), D_4_(2), D_5_(1), D_6 _(2), D_7_ (6), D_8_ (4), D_9_ (19), D_10_ (108), D_11_ (76), D_12_ (344), D_13_ (96), D_14_ (71), D_15_(112) adiabatic doublet states, the ground state here is being denoted as D_1_. The trajectories were propagated with the pySHARC module [66], using nuclear time steps of 0.5 fs [65], electronic time steps of 0.02 fs, the local diabatization propagator [94], and an energy‐based decoherence correction [95] (*α* = 0.1 au). During a surface hop, the kinetic energy was adjusted by rescaling the velocity vectors [96]. All trajectories reached the maximum simulation time and were included in the analysis.

### 5.7 Trajectory Analysis and SCO Kinetics

The trajectories were analyzed in terms of the electronic populations in the molecular Coulomb Hamiltonian representation (i.e., spin‐free states ordered by energy) [65]. We report the classical amplitudes transformed into this representation. For simplicity, in Figure 2e, we sum up populations of the same multiplicity. Section 5 in the Supporting Information presents the diabatic (ordered by character) electronic state populations of the simulations for the first 100 fs, including all states and accompanying discussions. Based on our dynamical simulation data, we extracted time constants for ISC, time constants for potential energy relaxation, and a set of key normal modes associated with SCO relaxation. The data extraction and analysis were performed using SHARC [89] and in‐house codes. For the calculated electronic populations, we applied a biexponential sequential model to determine the time constants for ISCs from doublet to quartet states and from quartet to sextet states. It is assumed that, at time zero, only the doublet states are populated as a result of an instantaneous δ‐pulse excitation. Using the energies and transition dipole moment values obtained at each time step, a TAS is calculated. The monoexponential kinetic fitting was performed on the obtained signal. The fitting curves and simulated TAS are presented in Figure S29. To identify the key vibrational coordinates, we performed a Fourier transform of the oscillatory signals from the time‐resolved electronic populations, the simulated TA spectrum, and the potential energy over time. The resulting signals are presented in Figures S27, S29, and 5.

### 5.8 Coherence mode analysis

The details of this transformation can be found in Supporting Information Section S7.

## Author Contributions

R.J.D.M. formulated and supervised the project. S.M., S.A.H., and R.J.D.M. did the planning and discussion of the experiments. S.M. performed the transient absorption (TA) experiment and the TA data analysis, including the time‐frequency analysis and Fourier‐filtering methods. K.T. provided the single crystals for the experiment. S.M. and S.A.H. microtomed the crystals for the TA experiment. S.M. and R.J.D.M. did the TA data interpretation with inputs from S.A.H., Y.J., and T.I. L.G. conceptualized, supervised, and discussed the theoretical part of the project. D.F. performed the quantum chemical calculations and nonadiabatic dynamics simulations, and analyzed the data. S.Mai. implemented the interface to parametrize the linear vibronic coupling model from RASSCF potentials, supported, and analyzed the dynamical simulations. S.M., D.F., L.G., and R.J.D.M. wrote the manuscript with inputs from all authors.

## Conflicts of Interest

The authors declare no conflicts of interest.

## Supporting information




**Supporting File 1**: anie72009‐sup‐0001‐SuppMat.docx.

**Supporting File 2**: anie72009‐sup‐0002‐Supporting_video_S1_164cm.mov.

**Supporting File 3**: anie72009‐sup‐0003‐Supporting_video_S2_238cm.mov.

**Supporting File 4**: anie72009‐sup‐0004‐Supporting_video_S3_273cm.mov.

**Supporting File 5**: anie72009‐sup‐0005‐Supporting_video_S4_1720cm.mov.

**Supporting File 6**: anie72009‐sup‐0006‐Supporting_video_S5_196cm.mov.

**Supporting File 7**: anie72009‐sup‐0007‐SI_Video_guide.txt.

## Data Availability

The data that support the findings of this study are presented in the manuscript and Supporting Information in graphical form. The datasets underlying the results presented in the manuscript are available at https://doi.org/10.5281/zenodo.19112788. Farkhutdinova, D., & Mitra, S. (2025). Data from the manuscript: “Elucidating the transition kernel and anharmonic coupling in the spin‐crossover process of a [Fe^III^(qsal)_2_] CH_3_OSO_3_ complex ” [Data set]. Zenodo.
