# Supplementary material for: Elucidating the Transition Kernel and Anharmonic Coupling in the Spin‐crossover Process of a [FeIII(qsal)2] CH3OSO3 Complex
Source: Angew Chem Int Ed Engl. 2026 Apr 1;65(20):e1079807. doi: 10.1002/anie.1079807 (PMC13159429; doi:10.1002/anie.1079807)
Supplement: Supplementary file 1 — Supporting File 1: anie72009‐sup‐0001‐SuppMat.docx. [file ANIE-65-e1079807-s003.docx]

**Supporting Information**

**Elucidating the transition kernel and anharmonic coupling in the spin-crossover process of a [Fe^III^(qsal)_2_] CH_3_OSO_3_ complex**

**Soumyajit Mitra^[a],§^** (ORCiD: [0009-0000-7239-1547](https://orcid.org/0009-0000-7239-1547))**, Dilara Farkhutdinova^[b],[c],§^** (ORCiD: [0009-0003-2012-1221](https://orcid.org/0009-0003-2012-1221))**, Sebastian Mai^[b]^** (ORCiD: [0000-0001-5327-8880](https://orcid.org/0000-0001-5327-8880))**, Stuart A. Hayes^[a],[d]^** (ORCiD: [0009-0007-6750-5563](https://orcid.org/0009-0007-6750-5563))**, Yifeng Jiang^[d],[e]^, Tadahiko Ishikawa^[f]^** (ORCiD: [0000-0001-7018-654X](https://orcid.org/0000-0001-7018-654X))**, Kazuyuki Takahashi^[g]^, Leticia González^[b]*^ (**ORCiD: [0000-0001-5112-794X](https://orcid.org/0000-0001-5112-794X)**) and R. J. Dwayne Miller**^[a]*^ (ORCiD: [0000-0003-0884-0541](https://orcid.org/0000-0003-0884-0541))

^[a]^Departments of Chemistry and Physics, University of Toronto, Toronto, Canada.

^[b]^Institute of Theoretical Chemistry, Faculty of Chemistry, University of Vienna, Vienna, Austria.

^[c]^Vienna Doctoral School in Chemistry (DoSChem), University of Vienna, Vienna, Austria

^[d]^European XFEL, Schenefeld, Germany.

^[e]^State Key Laboratory of Precision Spectroscopy, East China Normal University, Shanghai 200241, China

^[f]^Department of Chemistry, Institute of Science Tokyo: Tokyo, Japan.

^[g]^Department of Chemistry, Kobe University, Kobe, Japan.

^§^Equal Contribution

^*^Corresponding author: [leticia.gonzalez@univie.ac.at](https://utoronto-my.sharepoint.com/personal/soumyajit_mitra_mail_utoronto_ca/Documents/Desktop/laptop_desktop/Feqsal/NatChem/leticia.gonzalez@univie.ac.at); [dwayne.miller@utoronto.ca](mailto:dwayne.miller@utoronto.ca)

**Contents**

Section S1 Electronic Structure Calculation3

Section S2 Experimental Conditions6

Section S2.1 Experimental condition of Fe(III) complex in the transient absorption spectroscopy 6

Section S2.2 Calculation of excitation fraction9

Section S3 Data Analysis11

Section S3.1 Global Analysis 11

Section S3.2 Long-time data analysis16

Section S3.3 Time-Frequency Analysis 18

Section S3.4 Decay of the reactive modes 21

Section S4 Coordinate dependence of anharmonicity and non-linear mixing37

Section S4.1 Classical analog of non-linear mixing38

Section S5 Nonadiabatic dynamics simulation43

Section S6 Normal modes from simulated Transient Absorption spectrum46

Section S7 Coherence analysis48

Section S7.1 Total Standard Deviation48

Section S7.2 Coherence Standard Deviation49

Section S8. Bond length analysis51

Section S9. References52

**Section S1. Electronic structure calculations**

We carried out RASSCF (Restricted Active Space self-consisted field) (13, 2, 2; 4, 5, 5), where 13 corresponds to the total number of active electrons, 2 are the maximum number of holes in the RAS1 (Restricted Active Space) and electrons in RAS3, respectively, while 4, 5, and 5 correspond to the number of orbitals in the RAS1, RAS2, and RAS3 subspaces, respectively^[1]^. To speed up the integral calculations, the Cholesky decomposition (RICD) was employed^[2]^. The state-averaged RASSCF (13, 2, 2; 4, 5, 5) calculations were performed using the ANO-RCC-VDZP^[3,4]^ basis set at the optimized doublet ground-state geometry. Different active spaces were tested, and different partition schemes within active spaces were analyzed; the RASSCF (13, 2, 2; 4, 5, 5) option was chosen as the most accurate and cost-efficient one. The RAS1 active space is composed of two bonding metal-ligand sigma-type iron orbitals and two pi orbitals; the RAS2 contains the five 3d orbitals, and RAS3 has a full set of 4d orbitals (see Figure S1). Similar active spaces have been widely used in related transition metal complexes and are expected to provide a balanced description of the most important static and non-dynamic electron correlation^[5,6]^. Notably, the chosen active space can describe both metal-centered (MC) states as well as ligand-to-metal charge transfer (LMCT) states. The characters of the electronic states were analyzed in terms of charge transfer numbers computed with the libwfa program^[7]^.


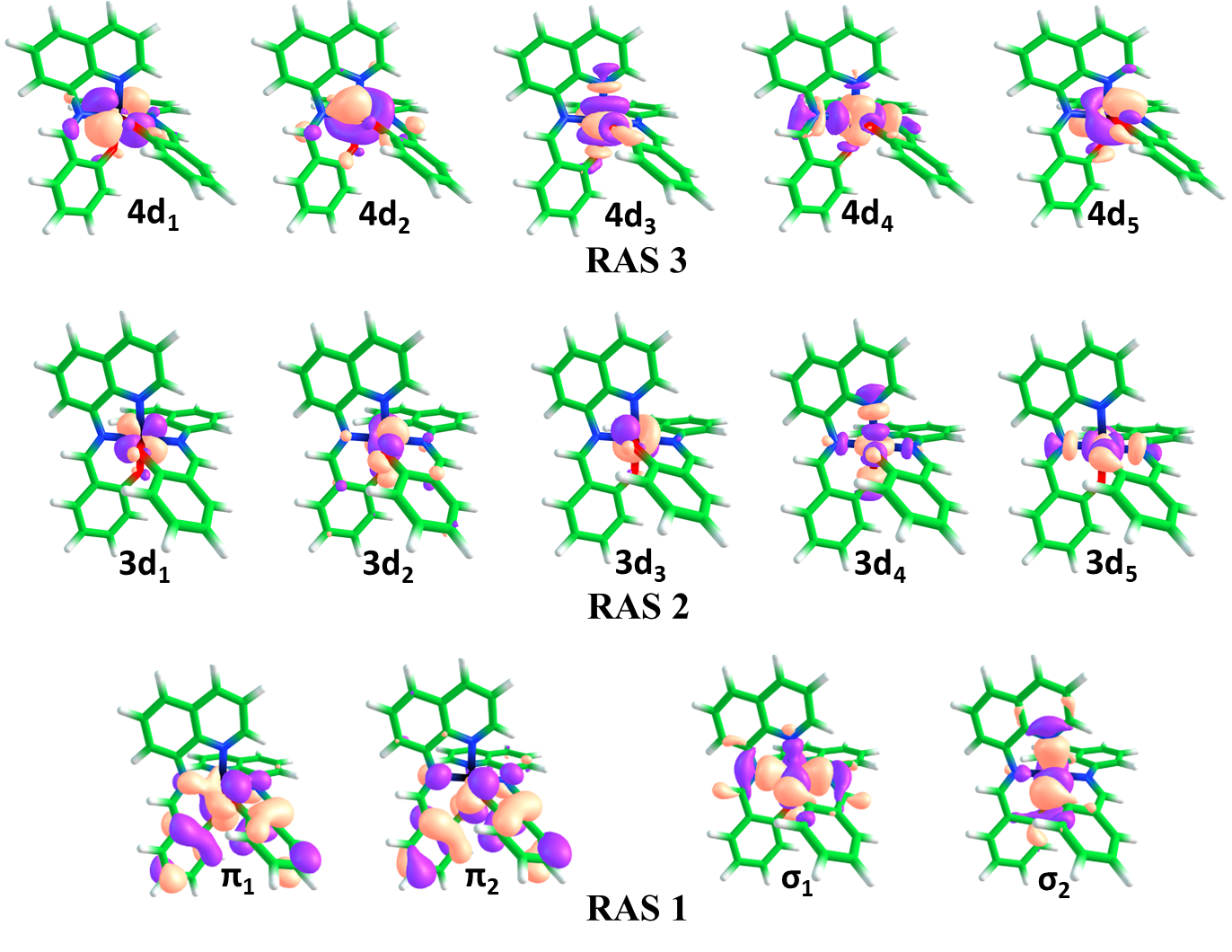


**Figure S1**. The RASSCF (13,2,2;4,5,5) active space natural orbitals. The RAS1 active space is composed of two bonding metal-ligand sigma-type iron orbitals and two pi orbitals; RAS2 contains five 3d orbitals, and RAS3 has a full set of 4d orbitals.

| **Doublets** | | | | **Quartets** | | | **Sextets** | | |
| --- | --- | --- | --- | --- | --- | --- | --- | --- | --- |
|  | **E, eV** | **f_osc_**  **10^-5^** | **State** |  | **E, eV** | **State** |  | **E, eV** | **State** |
| D_0_ | 0,00 |  | MC | Q_1_ | 0,70 | MC | 6_1_ | 0,95 | MC |
| D_1_ | 0,45 | 2,1 | MC | Q_2_ | 0,82 | MC | 6_2_ | 3,01 | LMCT |
| D_2_ | 0,51 | 1,5 | MC | Q_3_ | 1,04 | MC | 6_3_ | 3,02 | LMCT |
| D_3_ | 2,36 | 11,8 | MC | Q_4_ | 1,49 | MC | 6_4_ | 3,23 | LMCT |
| D_4_ | 2,39 | 20,3 | MC | Q_5_ | 1,70 | MC | 6_5_ | 3,25 | LMCT |
| D_5_ | 2,50 | 15,4 | MC | Q_6_ | 1,85 | MC | 6_6_ | 3,44 | LMCT |
| D_6_ | 2,64 | 10 | MC | Q_7_ | 2,99 | MC+LMCT | 6_7_ | 3,88 | LMCT |
| D_7_ | 2,67 | 18,1 | MC | Q_8_ | 3,02 | MC+LMCT |  |  | |
| D_8_ | 2,76 | 9,1 | MC | Q_9_ | 3,13 | MC |  |  | |
| D_9_ | 3,10 | 2,1 | MC | Q_10_ | 3,49 | MC |  |  |  |
| D_10_ | 3,33 | 1,8 | MC | Q_11_ | 3,51 | MC |  |  |  |
| D_11_ | 3,37 | 5,3 | MC | Q_12_ | 3,67 | MC |  |  |  |
| D_12_ | 3,58 | 5952 | LMCT | Q_13_ | 3,84 | LMCT |  |  |  |
| D_13_ | 3,75 | 7,1 | MC | Q_14_ | 3,86 | LMCT |  |  |  |
| D_14_ | 3,80 | 30,6 | MC | Q_15_ | 4,08 | MC |  |  |  |
| D_15_ | 4,02 | 671 | LMCT | Q_16_ | 4,13 | MC |  |  |  |
|  |  | | | Q_17_ | 4,22 | LMCT |  |  | |

**Table S1**. RASSCF (13,2,2;4,5,5) relative energies of the electronic states and oscillation strength for the doublet states, with relative energies (in eV) referenced to the ground doublet state (D₀).

**Section S2. Experimental Conditions**

**Section S2.1. Experimental condition of Fe(III) complex in the transient absorption spectroscopy**

Despite numerous studies of Fe(II) complexes in the solution state, performing ultrafast optical experiments in a single crystal environment is challenging due to the requirement for sample thickness to a few hundred nanometers since thicker samples will excessively attenuate the transmission of the optical probe beam and require higher excitation that leads to multiphoton processes that no longer give well defined initial excited state preparation^[8]^. The samples were excited with a 400 nm actinic pump pulse for transient absorption (TA) spectroscopy with an instrument response function (IRF) of 50 fs (σ of the IRF, Section S3.1). Moreover, the thickness of the sample (250 nm) is sufficiently thin to allow the transmission of the broadband white-light probe. The time resolution of this experiment was better than that of the previous experiment in ref. 9. The dispersive optics involving the third harmonic generation after the second harmonic generation was removed for this experiment. Moreover, the previous experiment in ref. 9 used a cryostat with a quartz window, which also acts as a dispersive optical material contributing to a larger time resolution ^[9]^. Our experiments were performed at room temperature (see Figure 1 in the main manuscript)

Crystal fatigue and reversibility are also important issues in single-crystal spectroscopic measurements, which can be overcome by using a lower repetition rate of the actinic pump^[10]^. In a single crystal TA experiment, the photoexcitation is confined to one area (210 μm, beam diameter) in the sample plane; hence, it is important to be cautious about the stability of the sample at the focal spot. For the TA measurements in [Fe^III^(qsal)_2_] CH_3_OSO_3_ samples, we used 125 Hz as the repetition rate for the actinic pump (Figure S2). A lower repetition rate for the actinic pump laser and fluence lower than the threshold limit for the non-linear regime is important for spectroscopic studies in solid-state systems (Figure S3). We conducted our transient absorption experiment at 1.67 mJ/cm^2^, which is below the threshold limit for non-linearity and with an excitation fraction of 10.56 % (Section S2.2)^[11]^. This ensured that all the molecules were fully relaxed to their initial thermally equilibrated ground state before the next cycle of actinic pulse excitation. The photoexcitation was done with a circularly polarized 400 nm pulse and probed with a linearly polarized broadband white-light ranging from 400 nm to 750 nm. The polarization of the white light was set to maximize the TA signal.


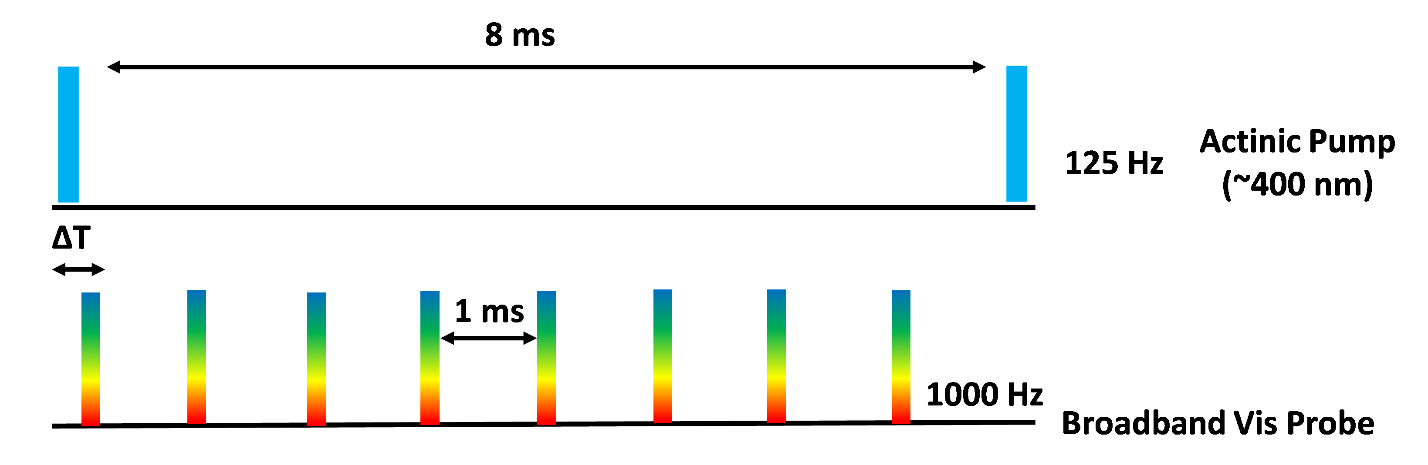


**Figure S2**. Schematics for the transient absorption experiment in [Fe^III^(qsal)_2_] CH_3_OSO_3_ single crystal SCO reaction. It involved a low repetition rate for the femtosecond laser excitation at 400 nm (125 Hz).


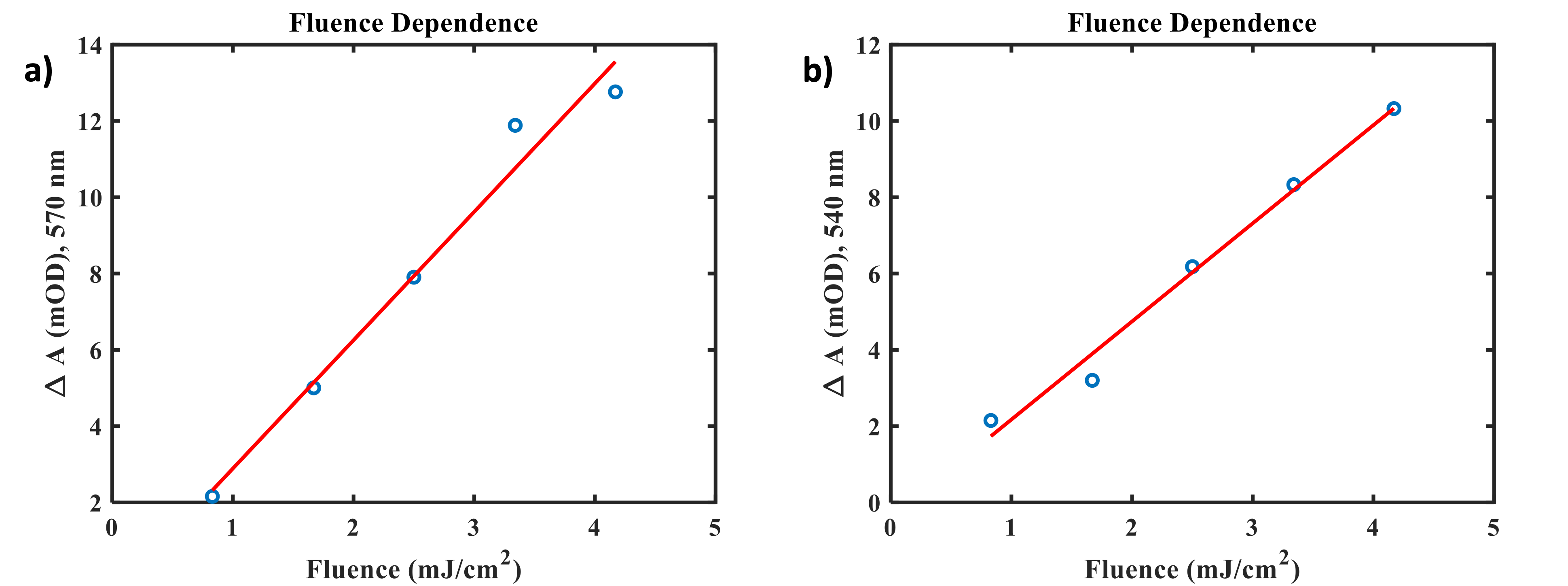


**Figures S3** (a) and **S3** (b). Fluence dependence of the TA signal at the wavelengths of 540 nm and 570 nm, respectively. Both plots show the linear regime of photoexcitation.

**Section S2.2. Calculation of excitation fraction**

For the calculation of excitation fraction for photoexcitation, one needs to calculate the absorption coefficient, and it is given in the units of cm^-1^ as:

$$\alpha=\frac{OD}{l (cm)}$$

Where $l$is the sample thickness in centimeters. With a crystal thickness of 250 nm and an absorbance of 1.11 at 400 nm, we get an absorption coefficient $\alpha$ of 44000 cm^-1^ for the [Fe^III^(qsal)_2_] CH_3_OSO_3_ derivative.

The absorption coefficient is related to the absorption cross-section by the following relationship

$$\sigma=\frac{\alpha}{n}$$

Where $n$ is the number density given by

$$n= \frac{\rho\left( g {cm}^{-3} \right)* N_{A} ({mol}^{-1})}{M_{r}(g {mol}^{-1})}$$

The density of the crystal $\rho$ is in g cm^-3^, which can be obtained from the CIF file of the crystal structure of [Fe^III^(qsal)_2_] CH_3_OSO_3_ derivative^[12]^. *N_A_* is Avogadro’s constant in mol^-1^, and *M_r_* is the molecular weight in g mol^-1^. Thus, the number density $n$ has the unit of cm^-3^, and when it is inserted in the above equation of $\sigma$, we get $\sigma$ in terms of cm^2^, which is the unit for cross-section. Using the above equation for $\sigma$, we have the absorption cross-section of 3.2*10^-17^ cm^2^ for the [Fe^III^(qsal)_2_] CH_3_OSO_3_ derivative.

The next step is to calculate the photon flux F,

$$F= \frac{Fluence \left( \frac{mJ}{{cm}^{2}} \right)*}{h* c}$$

Where is the wavelength of light, *h* is Planck’s Constant, and *c* is the speed of light. Thus, with a fluence of 1.67 mJ/cm^2^ and an excitation wavelength of 400 nm, we get a photon flux of 3.3*10^15^ cm^-2^.

The final step is to calculate the excitation fraction. The excitation fraction (%) = $\sigma*F*100$.

Thus, the excitation fraction for the [Fe^III^(qsal)_2_] CH_3_OSO_3_ derivative was 10.56 %.

The penetration depth of the sample is defined as,

$$\delta= \frac{1}{\alpha}$$

It is the depth at which the intensity of the incoming radiation inside the material falls to 1/e times its original value. For [Fe^III^(qsal)_2_] CH_3_OSO_3_, the penetration depth was 227 nm, which indicates a relatively homogenous excitation within the thickness of the crystal (250 nm).

**Section S3. Data Analysis**

**Section S3.1. Global Analysis**

Data analysis strategies are important for understanding the underlying dynamics of the TA spectroscopy data. It is important to deconvolve the incoherent noise from the data. This was achieved through the singular value decomposition method (SVD). The TA data is an M x N matrix in the form of ΔA (λ, t). SVD processes the ΔA into ΔA = USV^T^. Where U = U (λ) and V = V(t) are orthogonal matrices of size M x M and N x N, respectively^[13]^.

S is an M x N diagonal matrix. The U (λ) and V (t) contain the basis spectra and the respective kinetic traces of the data. Depending upon the value of the singular components shown in Figure S4, the first six components constituted the approximated TA data matrix (Figures S4a, S4b, and S4c), and the remaining components served as the incoherent noise floor of the data (Figure S4d).

After SVD truncation, the TA dataset is subjected to a global analysis procedure, which can provide the spectral information of the initial state, the intermediate states, and the final photoproduct state along with their temporal dynamics. This can be recovered from the evolution-associated difference spectra (EADS) using target analysis methods^[14]^.

The experimental data ΔA (λ, t) can be written in the matrix form ΔA (λ, t) = S(λ) T(t). If we multiply the inverse matrix T^-1^ from the right side, it gives the EADS vectors S = ΔA T^-1^. Since the matrix of the response function is highly non-symmetric, its inversion can be achieved through an SVD-based pseudoinversion method (the ‘*pinv*’ function in MATLAB was used to calculate the pseudoinverse of the response matrix).

S*_λ,i_* = $\sum_{t}$ ΔA*_λ,t_* T*_t,i_*^-1^ (summed over temporal index)

For the sequential model of the global analysis procedure, the response function is defined by the unbranched compartmental unidirectional model (A→ B→ C→ …→ n_comp_), also known as the sequential model. Each of these components (A→ B→ C→ …→ n_comp_) is called a species that is present in the evolution of the data and hence also referred to as EADS (Figure S5). In the sequential model, back reactions are ignored based on the assumption that the energy losses are large enough that reverse reaction rates are negligible.

The generalized parallel or sequential model can be solved using the following equations:

T = $\sum_{j=1}^{l} I \left( t \right) \bigotimes{b_{jl}e}^{-k_{j}t}$

$T \left( t \right)=0.5 \left[ \sum_{j=1}^{l} b_{jl}e^{-\left( \frac{t-t_{0}}{\tau_{j}} \right)}e^{\left( \frac{\sigma^{2}}{2\tau_{j}} \right)}\left[ 1+erf\left( \frac{t-t_{0}-\frac{\sigma^{2}}{\tau_{j}}}{\sqrt{2}\sigma} \right) \right] \right], k_{j}=\frac{1}{\tau_{j}};$

Where $t_{0}$ is the time zero of the signal, $\tau_{i}$ is the lifetime of the species, $\sigma$ is the width of the IRF, *I (t)*, (which was modelled as a Gaussian function), and *erf* is the error function, resulting from the convolution of the Gaussian with the Heaviside step function, *H (t-t_0_)*.

$k_{j}$ in the above equation, is the decay rate of the compartment j, and the amplitudes $b_{jl}$ in the sequential model ^[14]^ are defined by $b_{11}=1$ and for $j\leq l$ :

$b_{jl}= {\prod_{m=1}^{l-1} k_{m}}/{\prod_{n=1, n\neq j}^{l} {(k}_{n}-k_{j}})$

In particular, for $j<l$, $b_{j,l}= b_{j,l-1}k_{l-1}/(k_{l}-k_{j}).$

The fitting procedure was obtained by varying the fit parameters of τ*_i_* ’s (lifetime parameter) in the response function using the following minimization criterion:

$\sum$ (ΔA*_λ,t_* - $\sum_{i}$ S*_λ,i_* T*_t,i_*) → min.

The minimization algorithm for the EADS method was written in MATLAB 2021a, which involved the ‘lsqnonlin’ solver.


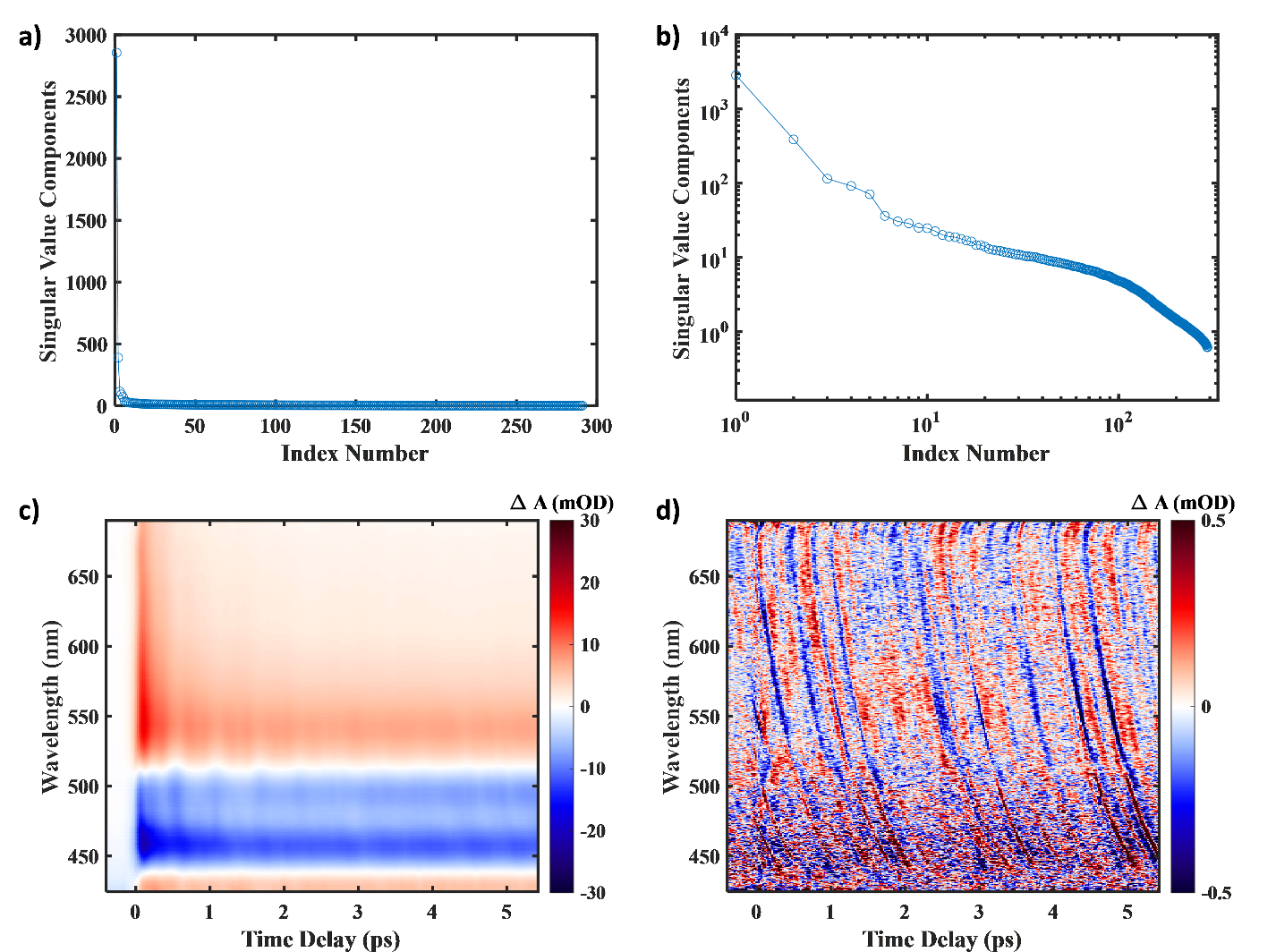


**Figure S4**. (a) and (b) Plot of the singular value components (SVD) versus index number, where the first six components comprise the data matrix, and the remainder of the SVD components make up for the noise floor. (c) The reconstituted data matrix with six SVD components was used for subsequent data analysis (shown in Figure 2c in the main manuscript), and (d) the residual matrix was obtained after subtracting the raw data matrix from the data matrix reconstituted from six SVD components, explained in Section S3.1.


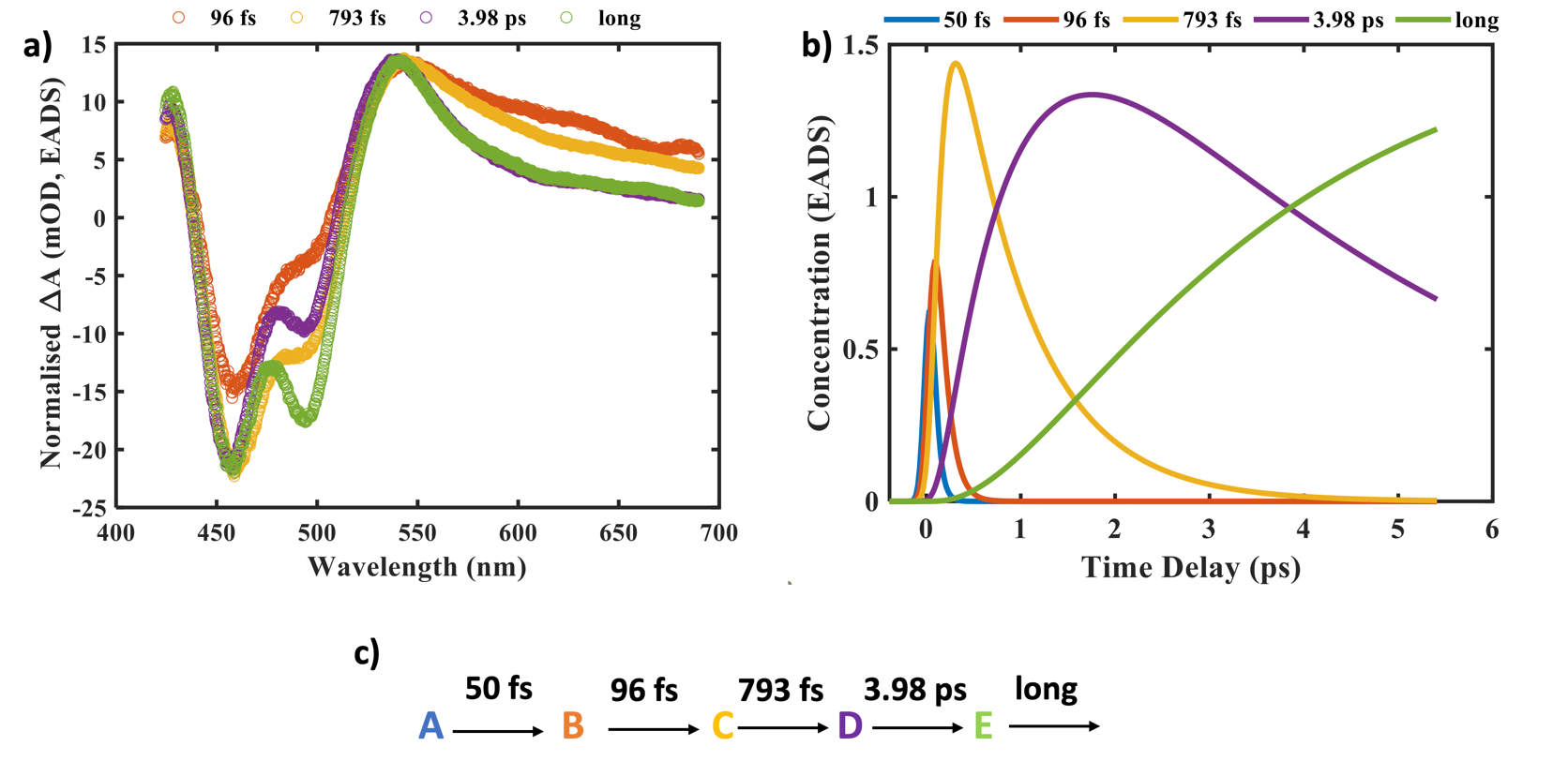


**Figure S5**. (a) Normalized EADS spectra, normalized to 544 nm. The EADS species associated with 793 fs undergoes spectral narrowing (narrower spectral feature of species D, relaxed minimum of HS state), indicating structural reorganization or an IVR (intramolecular vibrational relaxation) like mechanism^[15,16]^. (b) Concentration vs time plot for the EADS evolution from the global fitting procedure. Note: 50±2 fs, 96±3 fs, 793±8 fs, 3.98±0.37 ps.

**
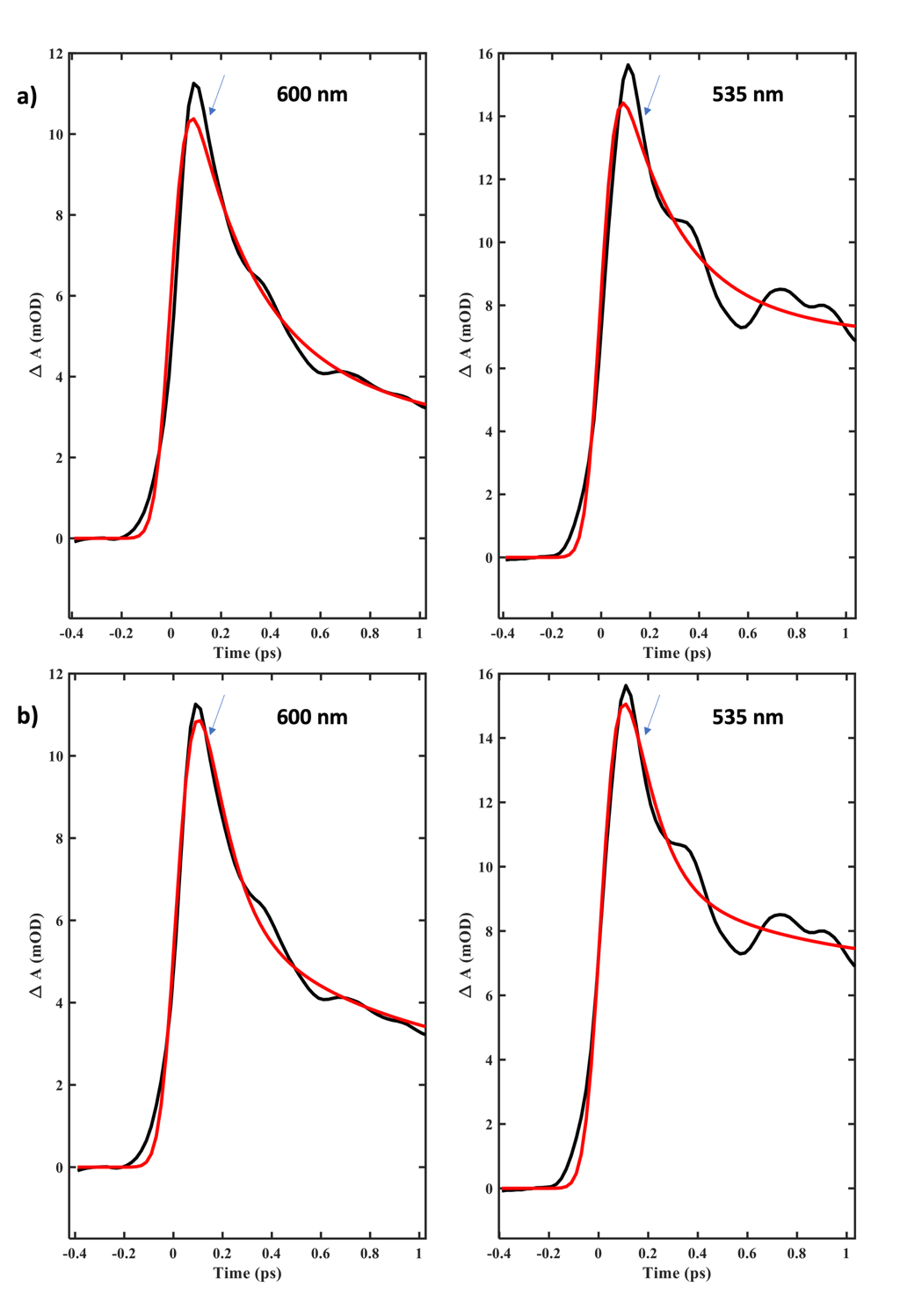
Figure S6**. (a) Illustrates EADS fitting with three components and a long, non-decaying one. The left and right panels show two different wavelengths. (b) EADS fitting with four components and a long, non-decaying one (Figure 2d main manuscript and Figure S5). The arrows highlight the initial fast component being fit better with the latter fit. The sequence of events in these fitting parameters also follows the Fe(III) dynamics explained in the previous report^[9]^.

**Section S3.2. Long-time data analysis**

**
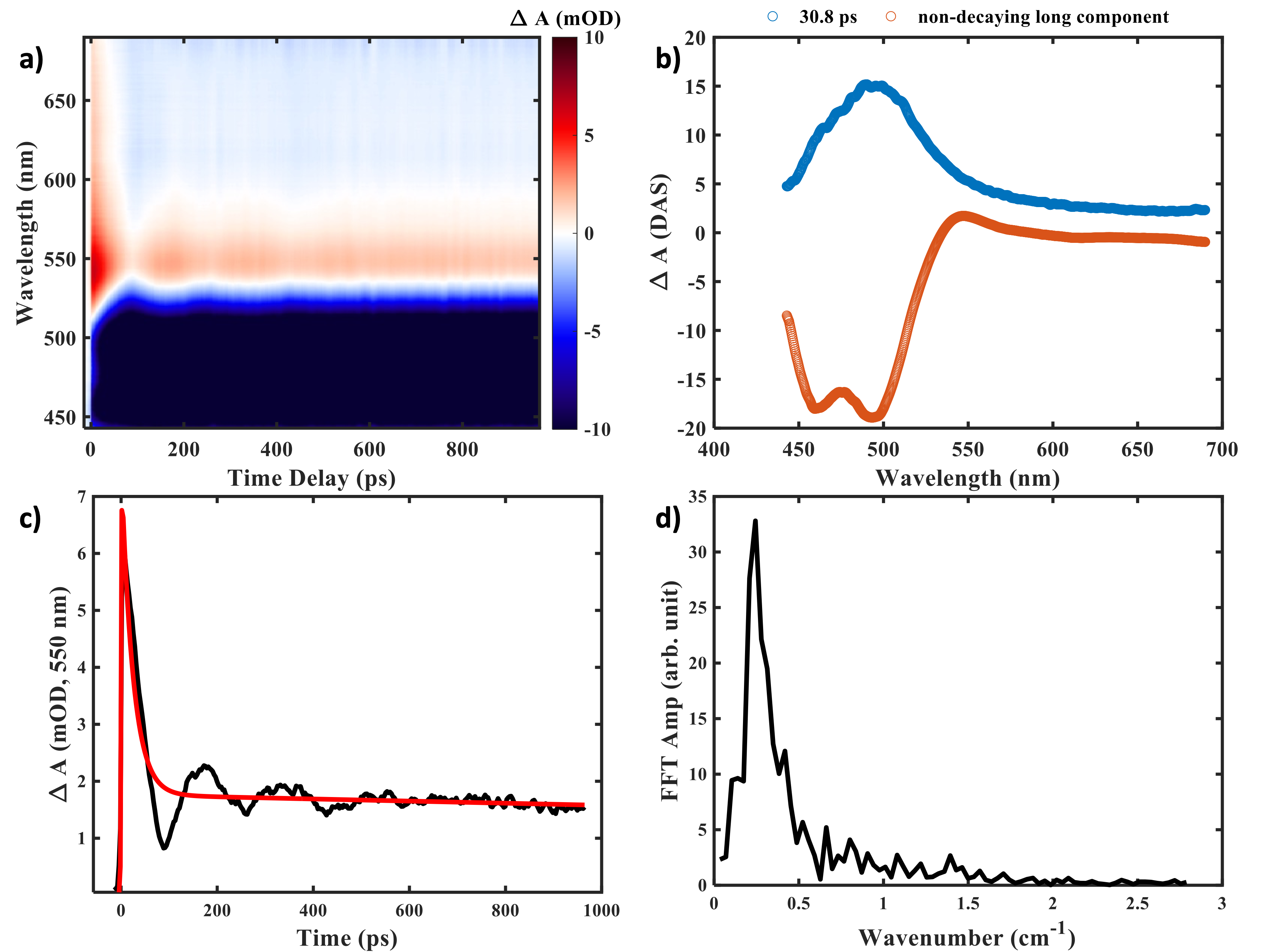
**

**Figure S7**. (a) 1 ns TA data of the [Fe^III^(qsal)_2_] CH_3_OSO_3_ derivative. (b) Decay Associated Difference Spectra (DADS) derived from the 1 ns TA data, showing a ~30 ps component and a non-decaying long component. (c) and (d) display the presence of a coherent acoustic phonon oscillation at 530 nm probe wavelength. (d) The frequency response of the residual.

The cooperative effect of heat dissipation in the [Fe^III^(qsal)_2_] CH_3_OSO_3_ crystal, resulting in lattice expansion, is indicative of lattice heating due to energy dissipation to the phonon bath (Figures S7a, S7b, and S7c). Previous observation of an optical phonon-mediated (28 cm^-1^) energy transfer to the surrounding lattice led to localized thermal expansion or molecular swelling^[17,18]^. We observed a similar mode (23 cm^-1^) across the probe wavelength range (Figure S10; lower panel), causing energy transfer from the photoexcited SCO state to the surrounding unit cells. This relaxation process is followed by volume expansion of the unit cell, leading to displacive forces on the surrounding unexcited unit cells in the lattice ^[18]^. These interactions, due to the change in internal pressure between the photo-switched HS molecules formed in a constrained LS lattice, activate acoustic phonons, which contribute to frequencies below 1 cm^-1^ in the FFT spectrum (Figure S7d). These frequencies correspond to acoustic modes from the thermal expansion of the crystal itself ^[17,19]^.

**Section S3.3. Time-frequency analysis**

The residual matrix obtained from the global analysis procedure (the sequential model) was fast Fourier transformed (FFT) to get the frequency response of the vibrational coherences excited by the actinic pulse (Figures 3a and 3b in the main manuscript). For the FFT analysis, a total time range of *t* = 5.6 ps was considered with a step size of Δ*t* = 20 fs. The maximum resolvable frequency (ν*_sample_*) or Nyquist frequency is ν*_sample_* = 1/ (2. Δ*t*. *c*) =833.92 cm^-1^, where *c* is the speed of light. The spectral resolution is calculated as follows: Δν = 1/ (*t*. *c*) = 5.95 cm^-1 [20,21]^ .

As mentioned in the main manuscript, we selected three wavelengths for the time-frequency analysis spanning three different ranges of the TA spectrum^16^. Each trace was averaged over a wavelength range of 5 nm. For the spectral filtering in the frequency domain, let’s look at Figure S9a. The 520 nm residual trace is fast Fourier transformed (Figure S9b), and we find the presence of an intense peak at 65 cm^-1^. The intense peak is filtered in the frequency domain as shown in Figures S9b and S9c. The filtered frequency domain response is further inverse Fourier transformed to get the temporal dynamics of the 65 cm^-1^ peak (Figure S9d). This method is also employed for residuals at different wavelengths (490 nm, 520 nm, and 600 nm; Figure S10), with varying bandwidths of the frequency domain filters, to obtain information on assistive and reactive modes (Figures S11, S14, and S17). The identified wavelengths for the time-frequency analysis of the transient residual signals represent the ground-state bleach (GSB: 490 nm) and the excited-state absorption region (ESA: 520 and 600 nm) (see Figure S10). For 490 nm and 520 nm transient residual signals, the frequency domain spectrum was divided into four parts (Figures S11 and S14), and the 600 nm spectrum was divided into two parts (Figure S17). The spectral windows were chosen to keep in mind the temporal dynamics of the growing amplitude of the oscillations^16^.

These Fourier-filtered temporal responses are fitted with a sum of decaying sinusoidal functions. Depending upon the amplitude and the decay times of these sinusoidal curves, judgments on the assignments of reactive and assistive modes are made (Table S2). Oscillation amplitudes greater than or equal to 10 % were only used in the assignment procedure^[16,22,23]^. A pictorial overview of the time-frequency data analysis scheme is shown in Figure S8.

The sum of decaying sinusoidal functions was used for the fitting procedure:

F = $\sum_{i} A_{i} e^{{-t}/{\tau_{i}}}sin( \omega_{i}t+\varphi)$;

Depending upon the input parameters (A*_i_*, τ*_i_*, ω*_i_*, φ*_i_*), fitting was performed using a MATLAB-based algorithm involving the ‘lsqcurvefit’ solver.

The goodness of the fit in the time domain was judged based on R^2^ values. The important modes related to the SCO process are observed during this analysis, and their relevance to the reaction coordinate is judged based on their amplitudes and decay times.

**
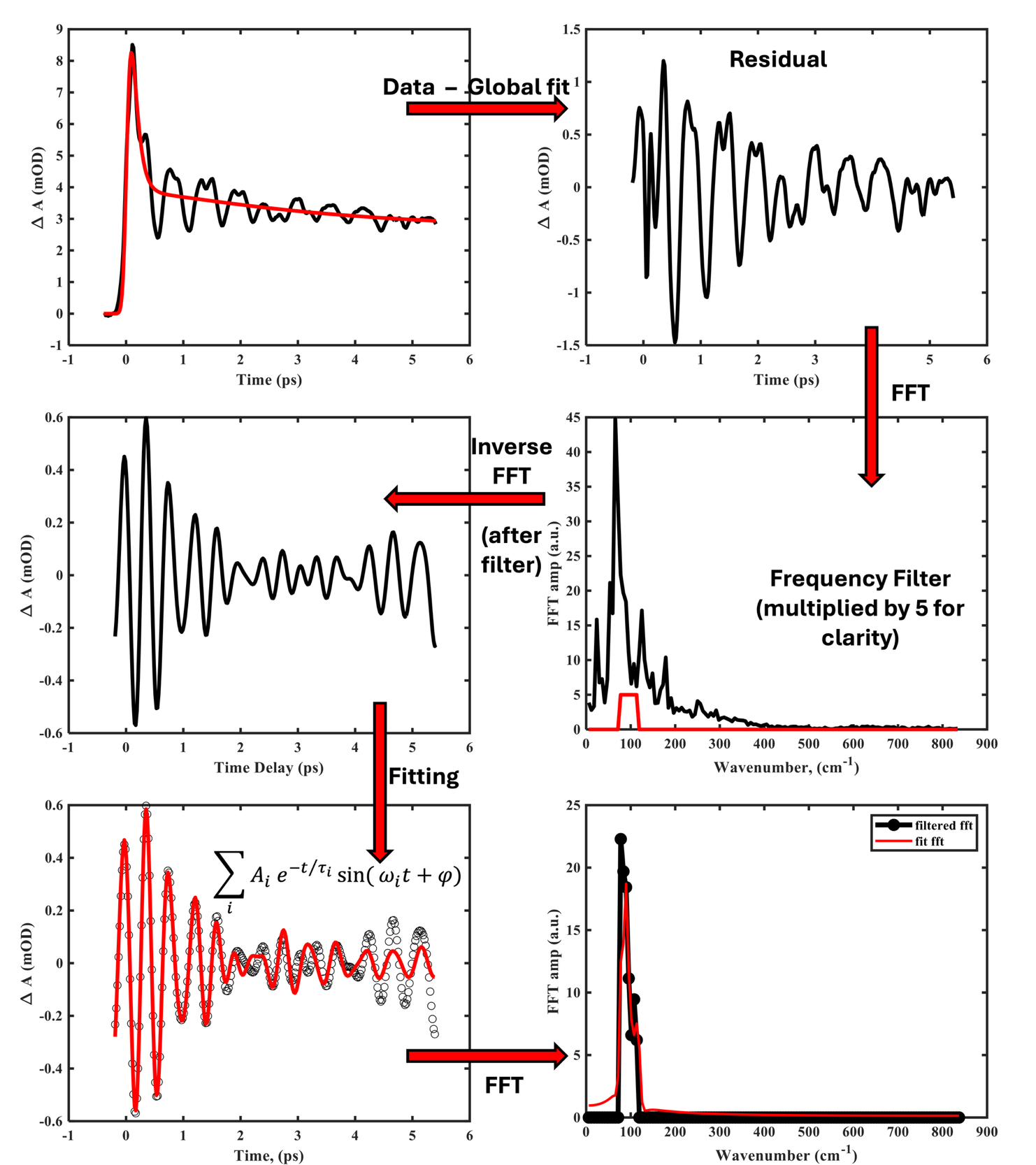
Figure. S8**. The above figure highlights the data analysis scheme utilized in this paper (The frequency domain filter is multiplied five times for visualization clarity).

**Section S3.4. Decay of the key reactive modes**

The total dephasing time $t_{2}$ is measured by $\frac{1}{t_{2}}=\frac{1}{{2t}_{1}}+\frac{1}{t_{2}^{*}}$ , where $t_{1}$ is the population decay and $t_{2}^{*}$ is the pure dephasing time. For crystals, the pure dephasing time $t_{2}^{*}$ is longer since there is no solvent interaction. However, if the population decay $t_{1}$ is very fast (<100 fs in SCO) compared to the pure dephasing time $t_{2}^{*}$ ( $t_{1}\ll t_{2}^{*}$ ), then the total dephasing rate is approximately comparable to the population decay time. Hence, if molecular modes or vibrations decay on a similar timescale to the SCO reaction, then they can be considered the key reactive mode driving the chemical transformation through the curve-crossing region. The nearly identical decay dynamics for these vibrational modes indicate dissipation leading to the displacements involved in molecular reorganization in response to the changes in the spin-states or electronic distribution.


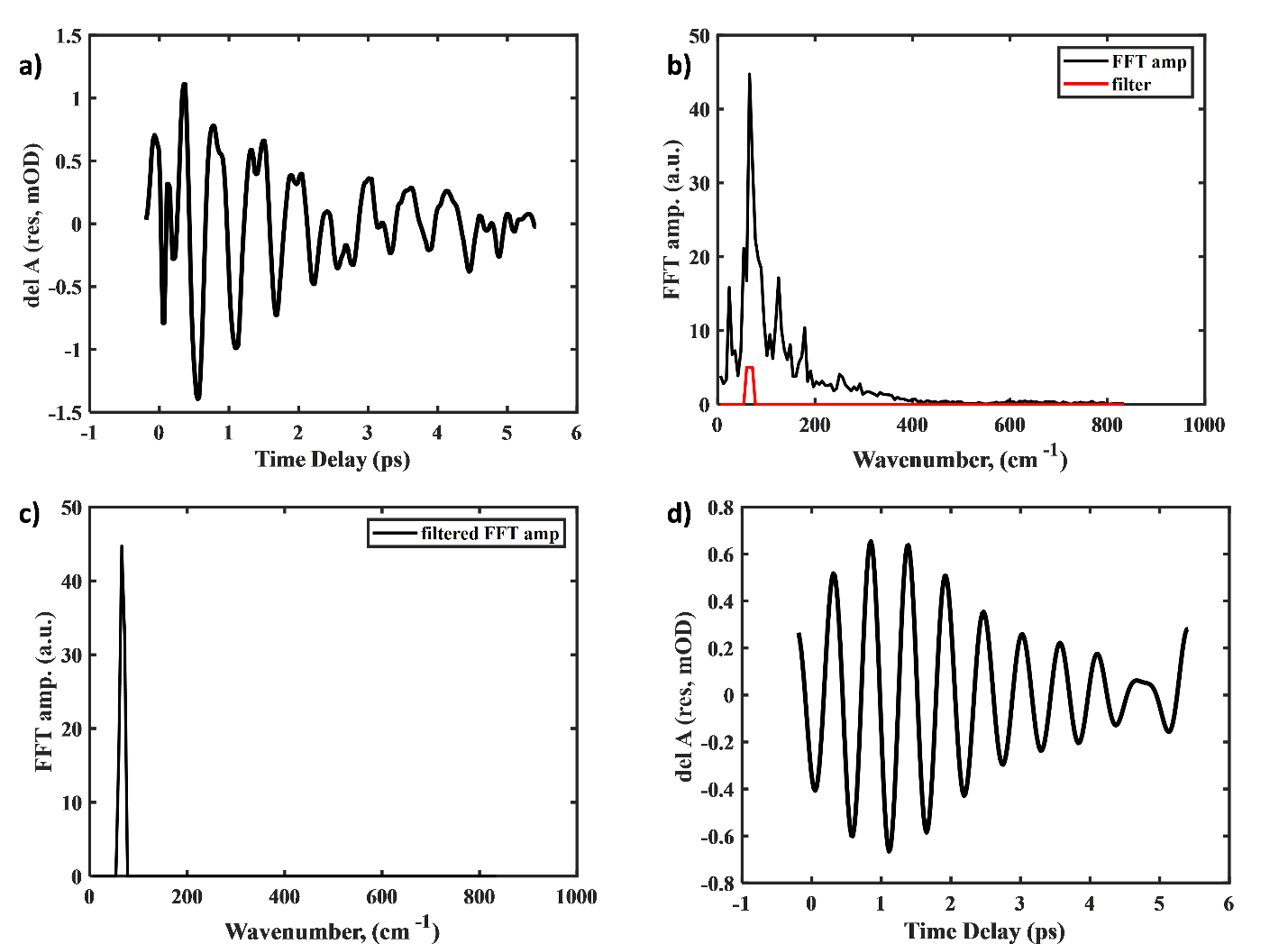


**Figure S9**. (a) Residual at 520 nm. (b) FFT spectrum of the residual, along with a Fourier domain filter centered around 65 cm^-1^ (The filter is multiplied five times for visualization clarity). (c) Filtered frequency, and (d) temporal evolution of the filtered frequency after inverse Fourier transform. Detailed explanation in Section S3.3.


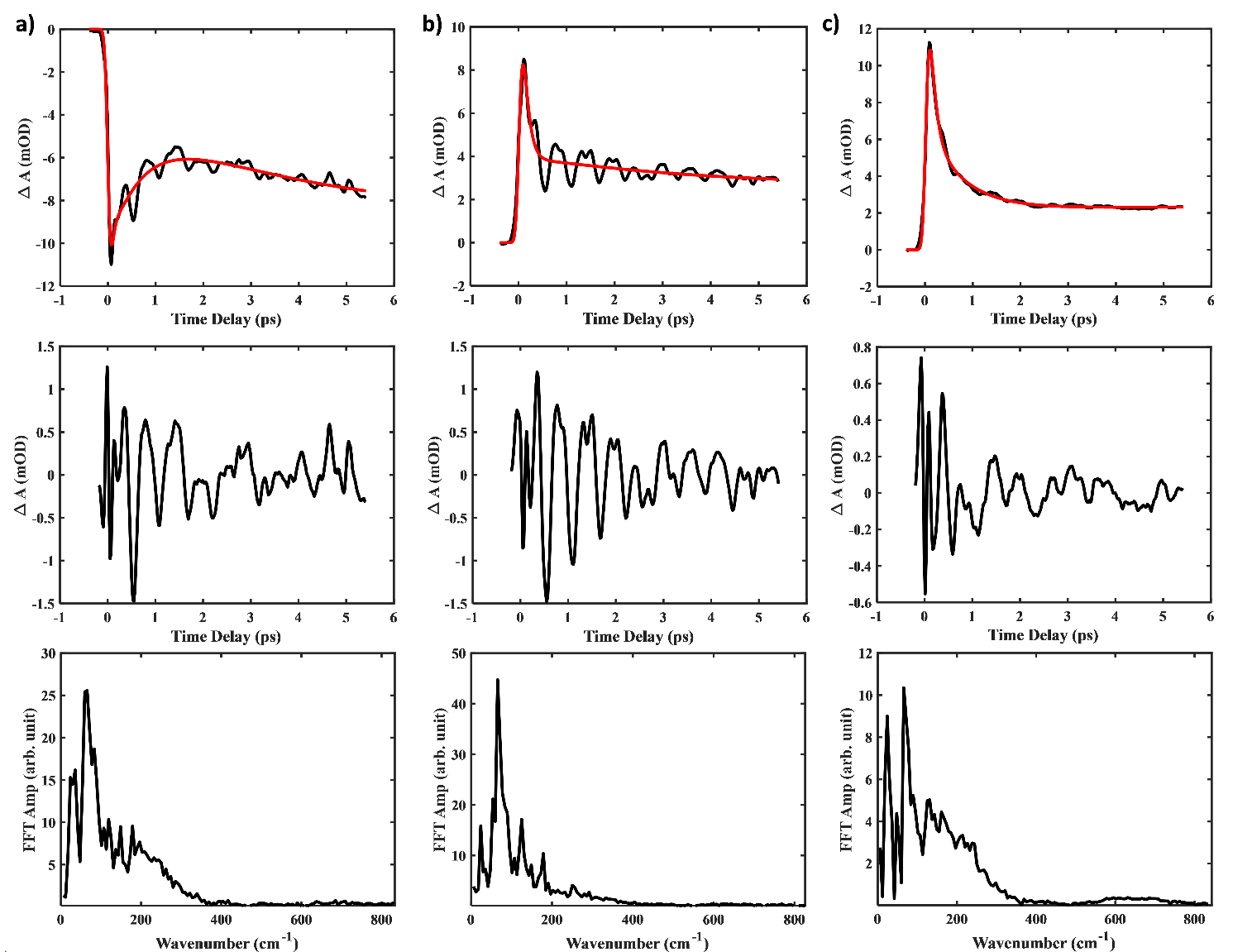


**Figure S10**. Top panel: Transient absorption, ∆A, signal at selected wavelengths (a) 490 nm, (b) 520 nm, and (c) 600 nm, respectively. Middle panel: Coherent vibrational wavepacket dynamics overriding the electronic dynamics in the ∆A signal at selected wavelengths (a) 490 nm, (b) 520 nm, and (c) 600 nm, respectively. Bottom panel: Frequency response of the wavepacket dynamics observed at selected wavelengths (a) 490 nm, (b) 520 nm, and (c) 600 nm, respectively.


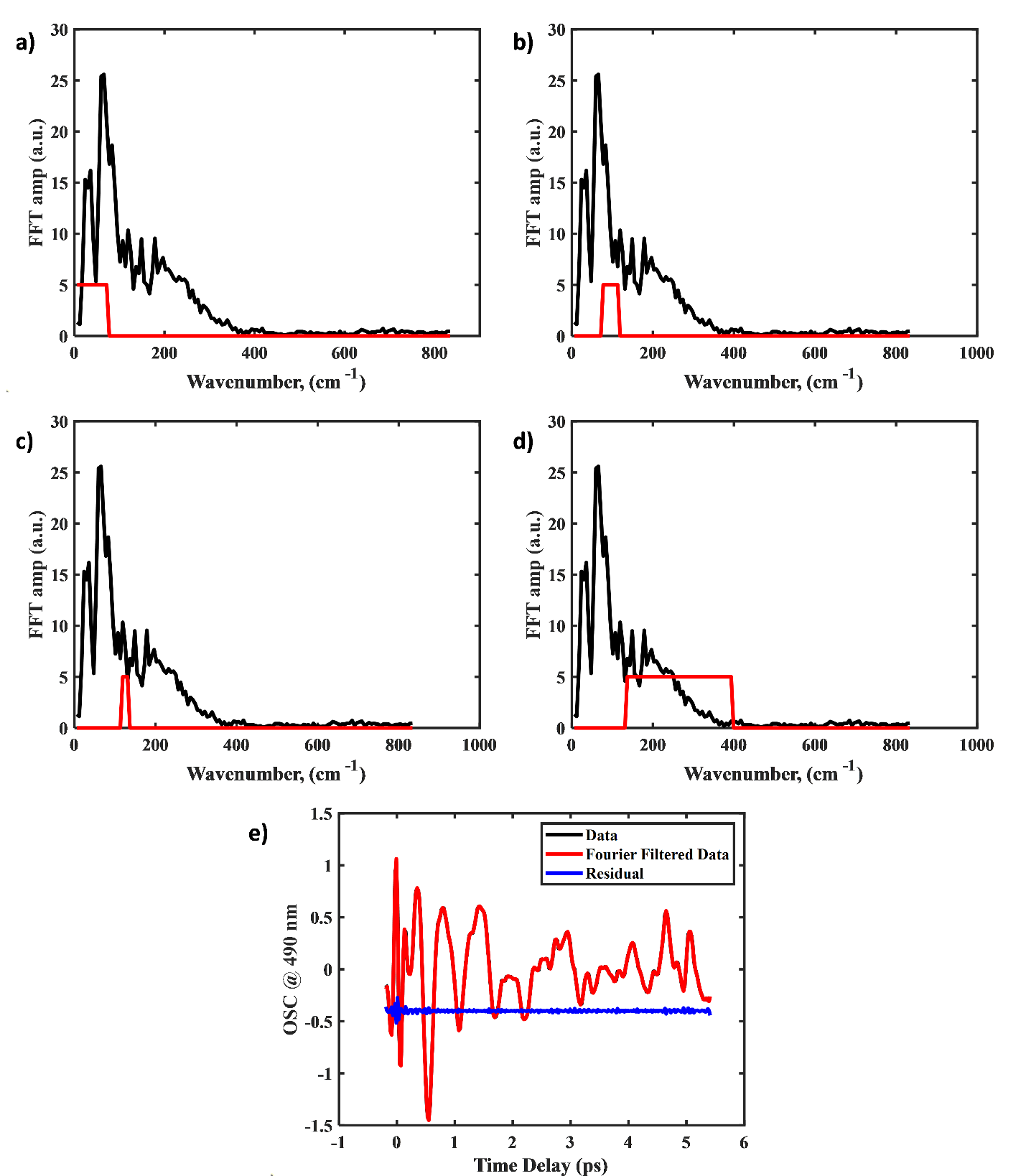


**Figure S11**. (a), (b), (c), and (d) show four different filtering ranges for the FFT spectrum at 490 nm (The filter is multiplied five times for visualization clarity). Filtered ranges shown in Figures S11a and S11c have rising amplitude and, hence, are not fitted with a decaying sinusoidal function. (c) Overlay of the actual residual along with the sum of the Fourier filtered data. The residual part of the plot reflects the unused frequencies, whose contributions are negligible to the overall vibrational dynamics.


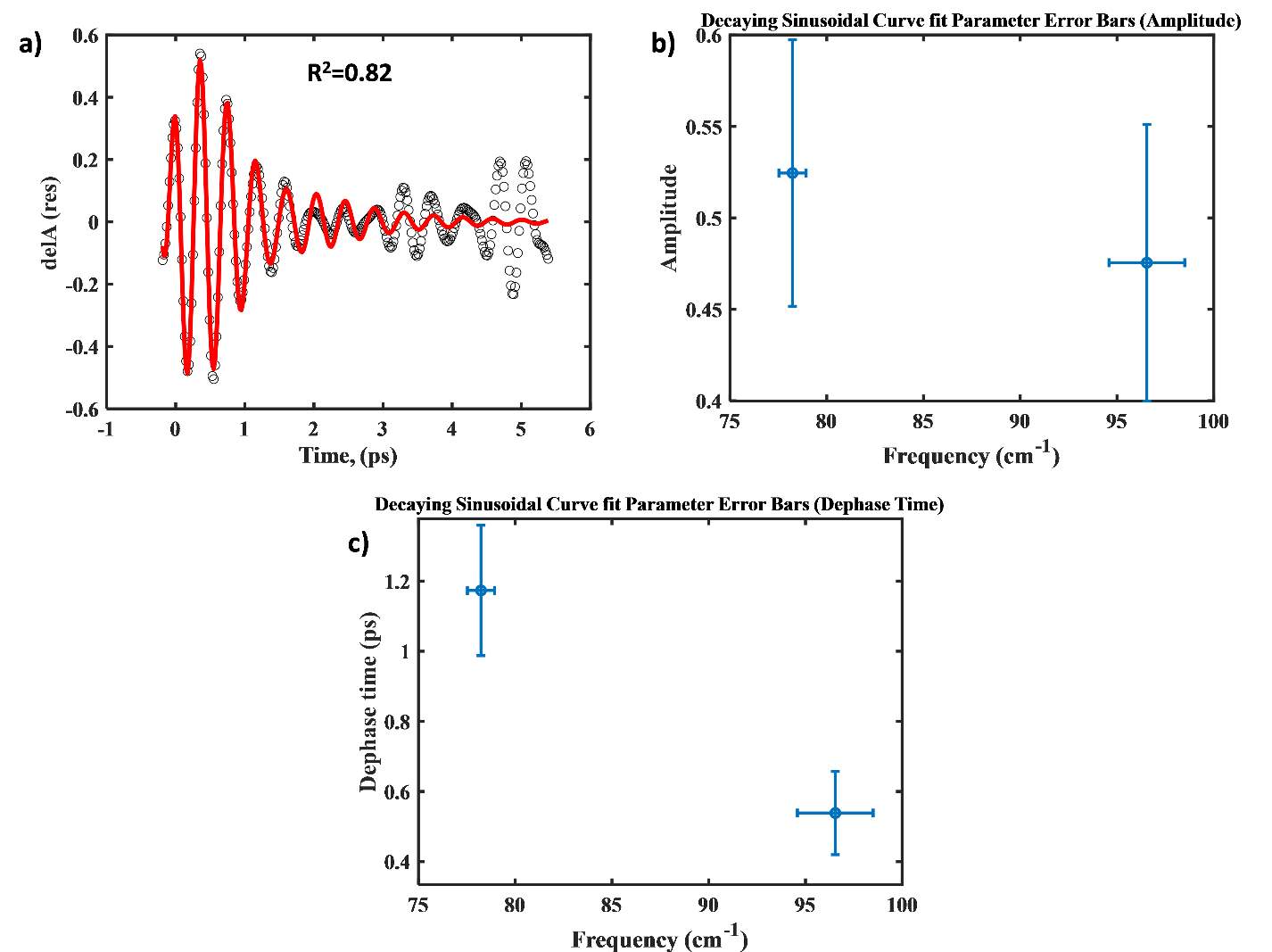


**Figure S12**. (a) Decaying sinusoidal fit of the Fourier filtered data in Figure S11b. (b) Error bars of the fit, amplitude vs frequency. (c) Error bars of the fit, dephasing time vs frequency. Amplitudes less than 10% have large error bars in the dephasing time. They comprise the non-decaying components in the fitting with large error bars in the dephasing time.


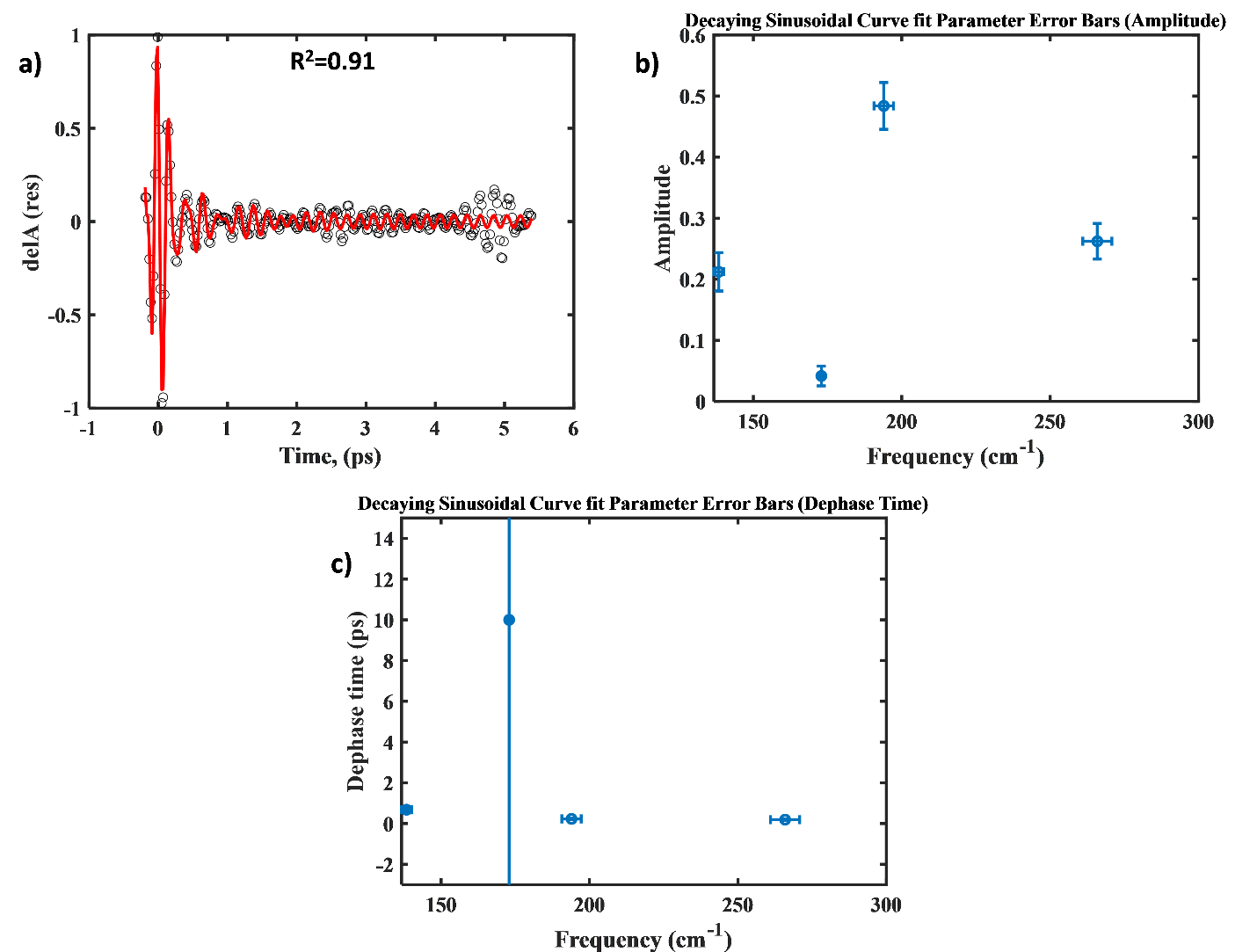


**Figure S13**. (a) Decaying sinusoidal fit of the Fourier filtered data in Figure S11d. (b) Error bars of the fit, amplitude vs frequency. (c) Error bars of the fit, dephasing time vs frequency. Amplitudes less than 10% have large error bars in the dephasing time. They comprise the non-decaying components in the fitting with large error bars in the dephasing time.


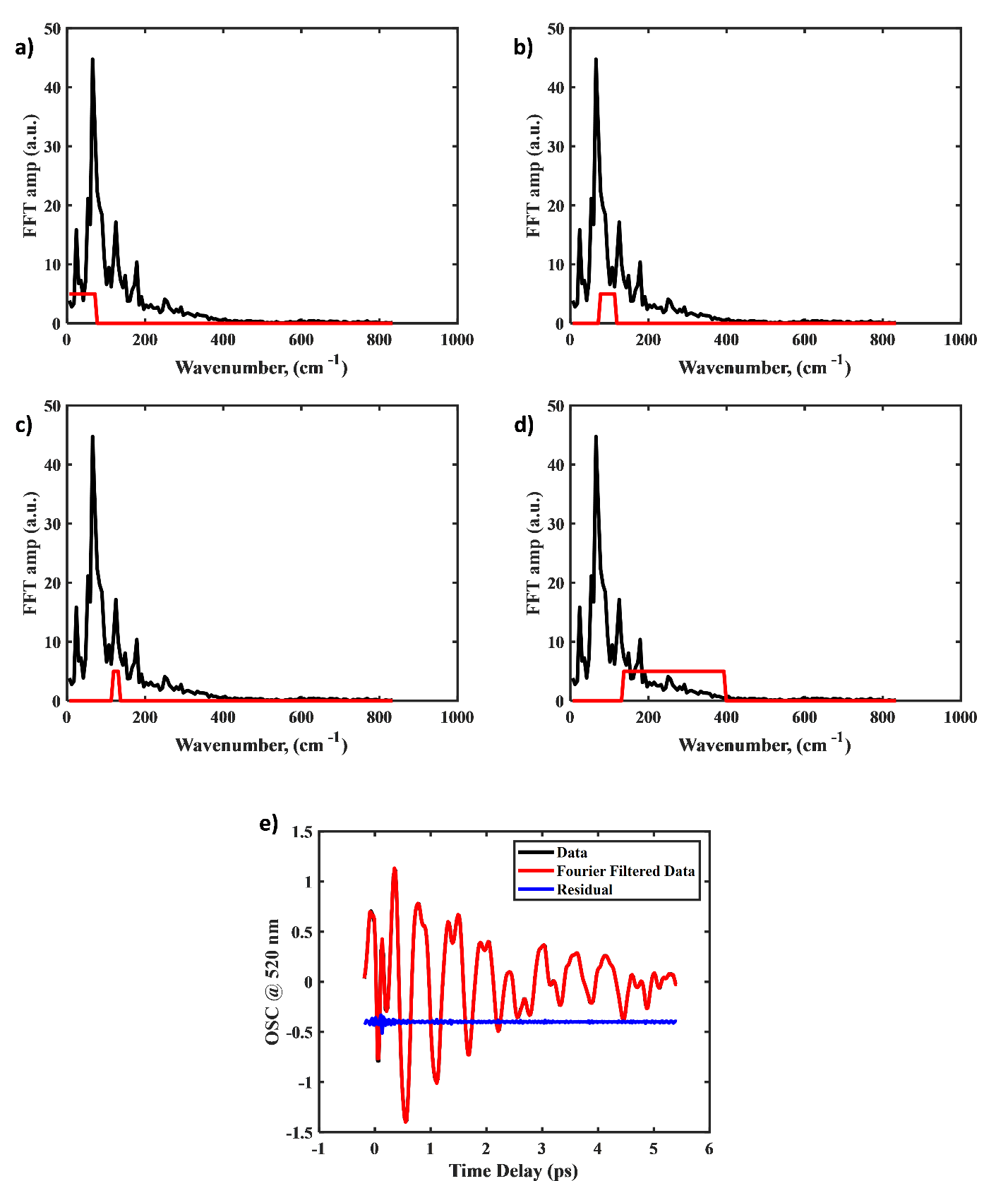


**Figure S14**. (a), (b), (c), and (d) show four different filtering ranges for the FFT spectrum at 520 nm (The filter is multiplied five times for visualization clarity). Filtered ranges shown in Figures S14a and S14c have rising amplitude and, hence, are not fitted with a decaying sinusoidal function. (c) Overlay of the actual residual along with the sum of the Fourier filtered data. The residual part of the plot reflects the unused frequencies, whose contributions are negligible to the overall vibrational dynamics.


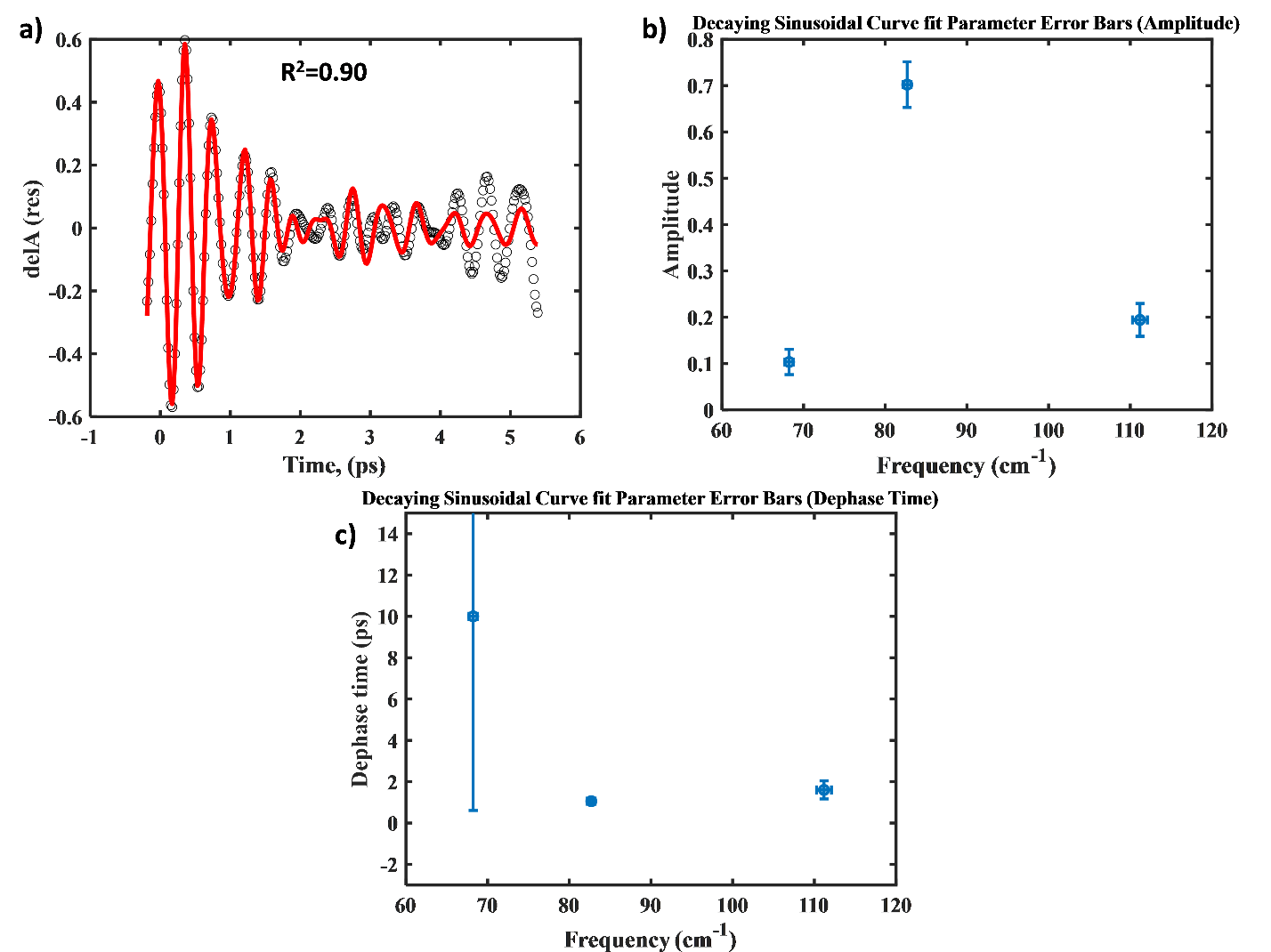


**Figure S15**. (a) Decaying sinusoidal fit of the Fourier filtered data in Figure S14b. (b) Error bars of the fit, amplitude vs frequency. (c) Error bars of the fit, dephasing time vs frequency. Amplitudes less than 10% have large error bars in the dephasing time. They comprise the non-decaying components in the fitting with large error bars in the dephasing time.


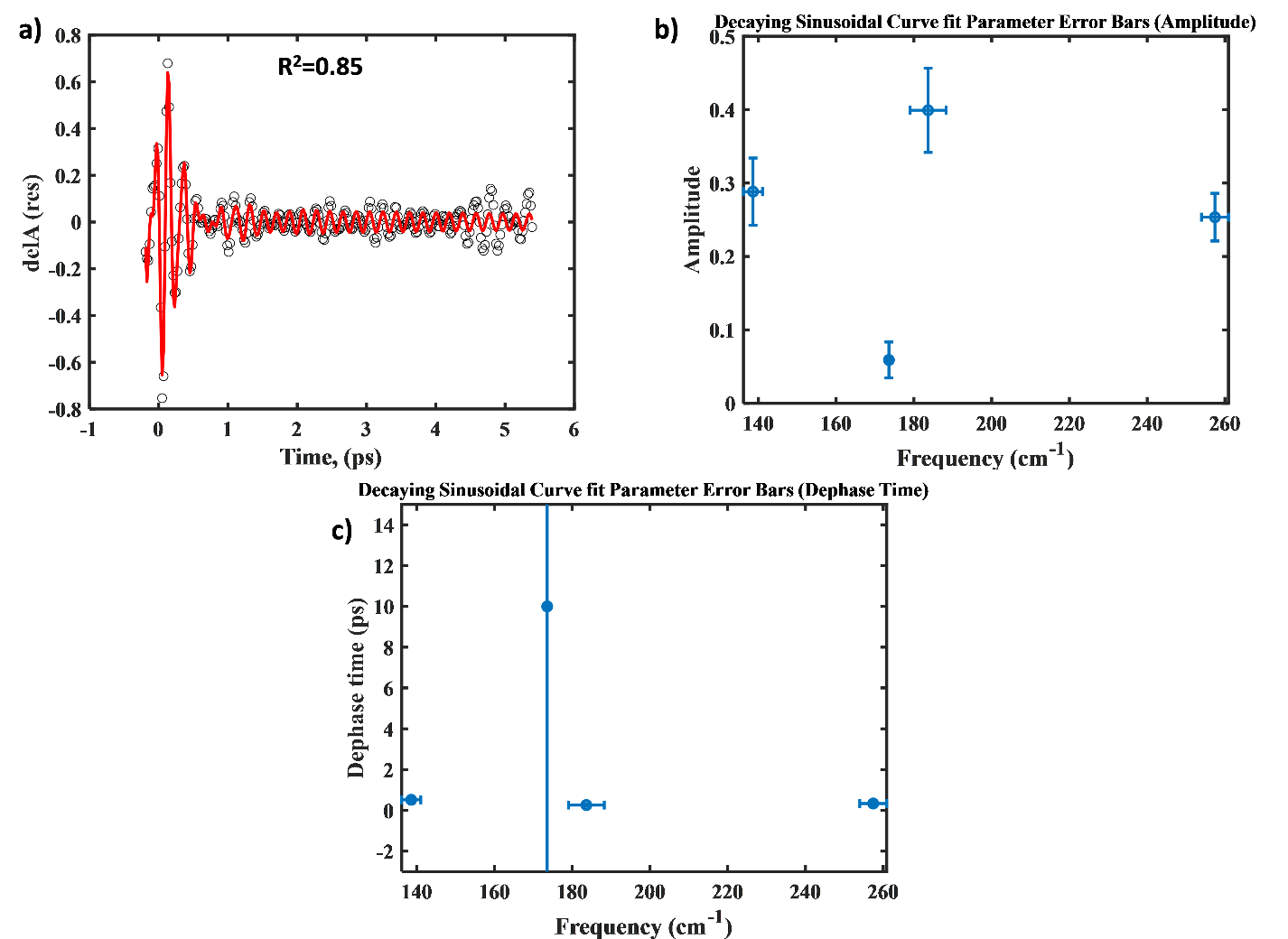


**Figure S16**. (a) Decaying sinusoidal fit of the Fourier filtered data in Figure S14d. (b) Error bars of the fit, amplitude vs frequency. (c) Error bars of the fit, dephasing time vs frequency. Amplitudes less than 10% have large error bars in the dephasing time. They comprise the non-decaying components in the fitting with large error bars in the dephasing time.


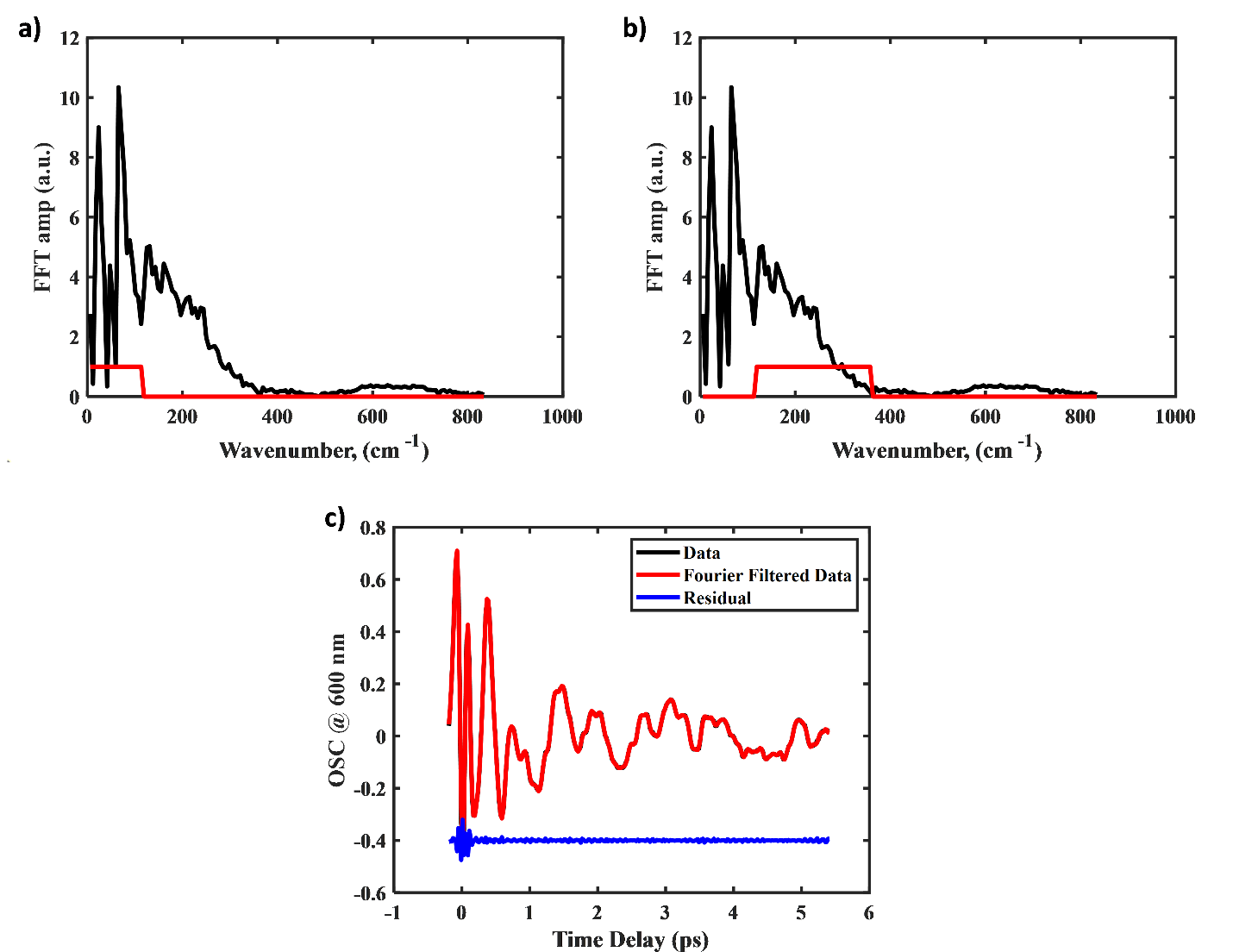


**Figure S17**. (a) and (b) show two different filtering ranges for the FFT spectrum at 600 nm. (c) Overlay of the actual residual along with the sum of the Fourier filtered data. The residual part of the plot reflects the unused frequencies, whose contributions are negligible to the overall vibrational dynamics.


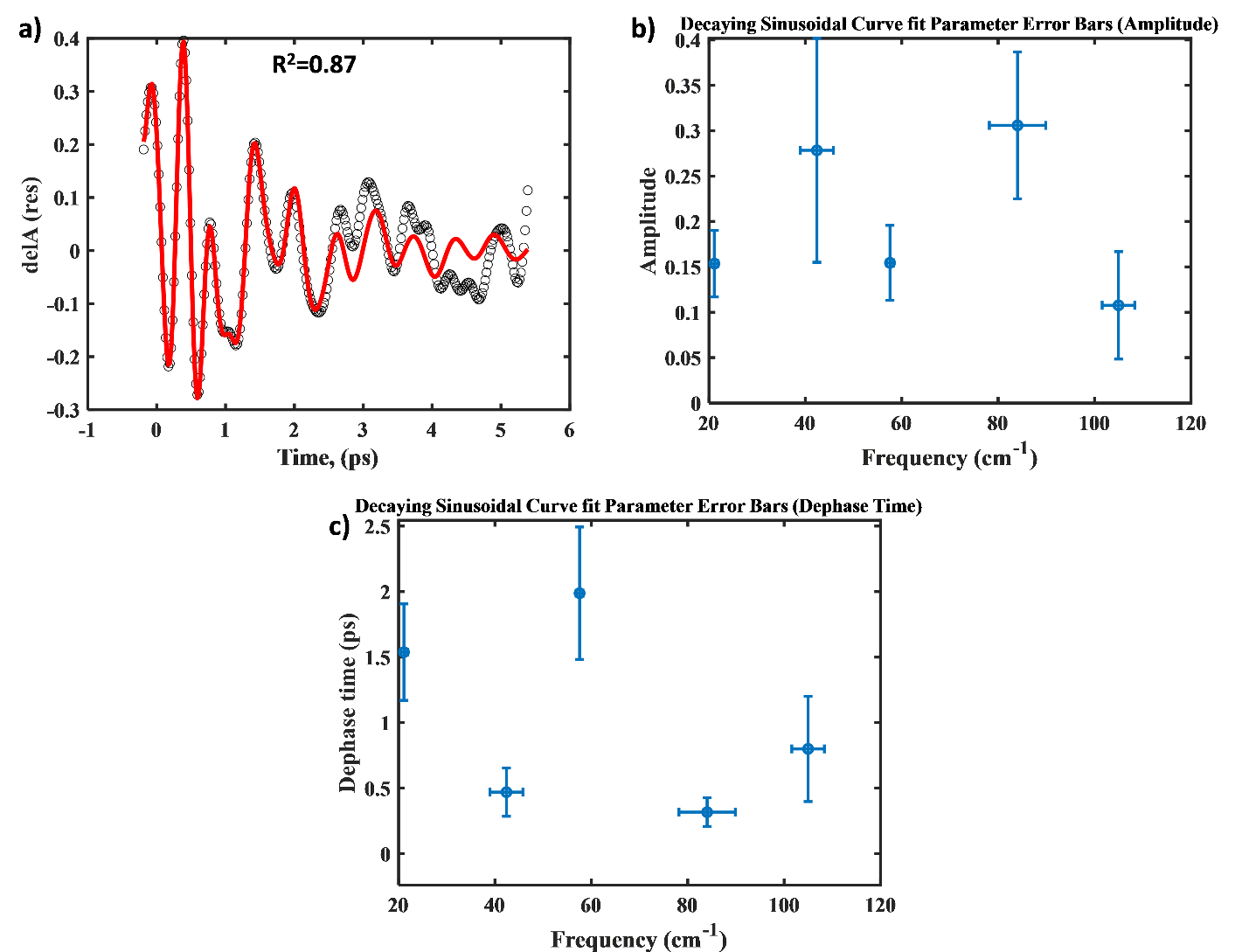


**Figure S18**. (a) Decaying sinusoidal fit of the Fourier filtered data in Figure S17a. (b) Error bars of the fit, amplitude vs frequency. (c) Error bars of the fit, dephasing time vs frequency. Amplitudes less than 10% have large error bars in the dephasing time. They comprise the non-decaying components in the fitting with large error bars in the dephasing time.


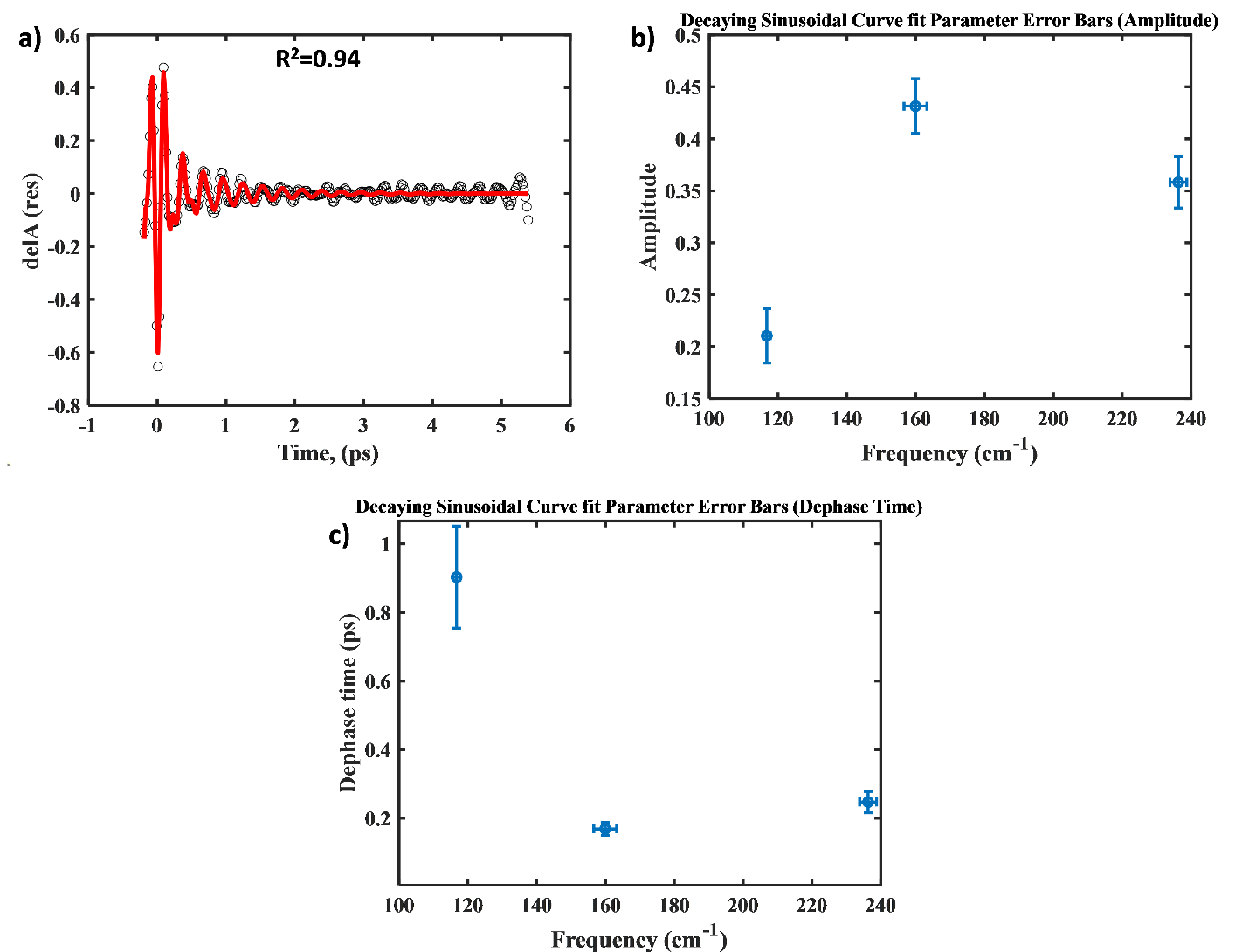


**Figure S19**. (a) Decaying sinusoidal fit of the Fourier filtered data in Figure S17b. (b) Error bars of the fit, amplitude vs frequency. (c) Error bars of the fit, dephasing time vs frequency. Amplitudes less than 10% have large error bars in the dephasing time. They comprise the non-decaying components in the fitting with large error bars in the dephasing time.


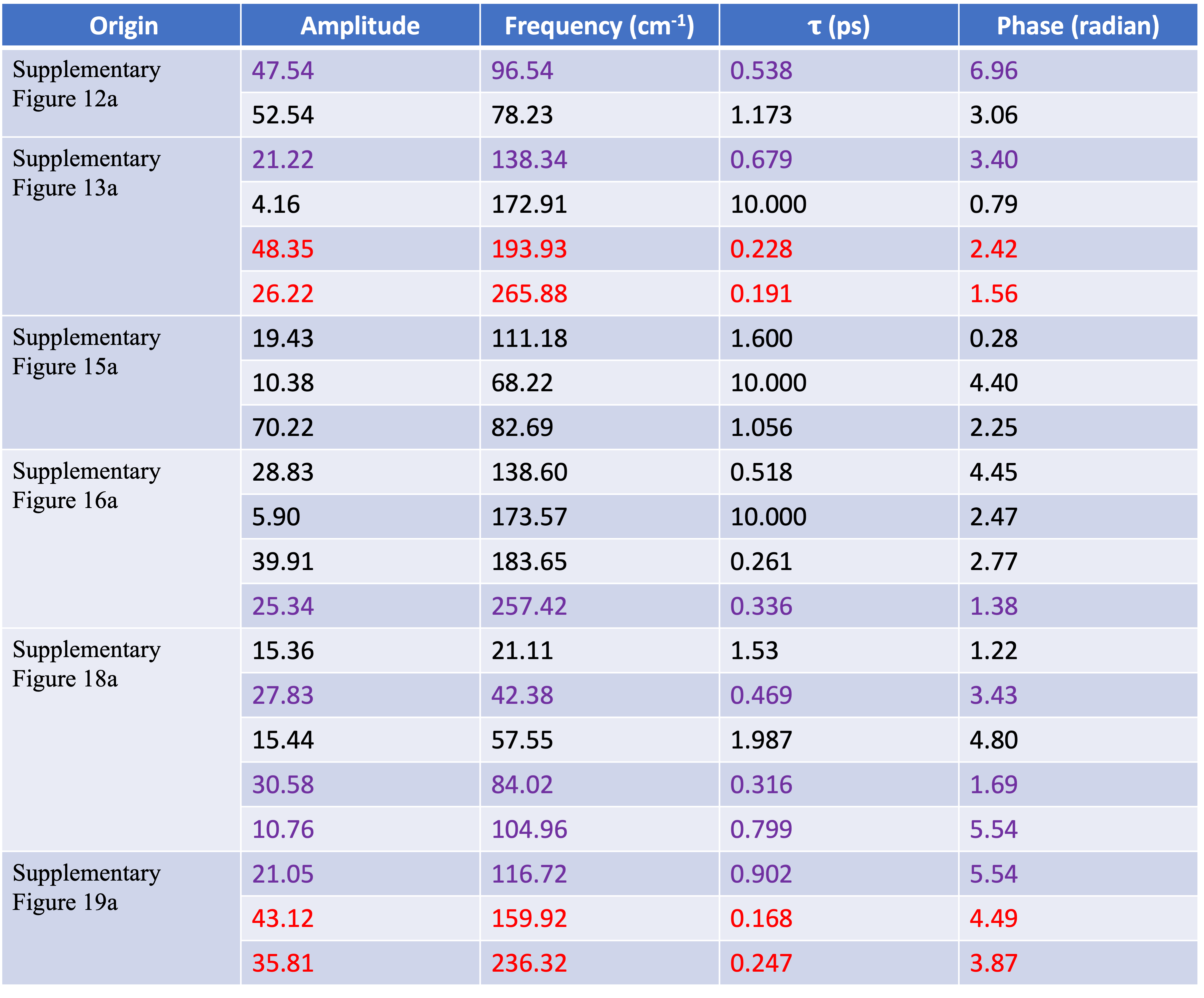


**Table S2**. The above parameters (A*_i_*, τ*_i_*, ω*_i_*, φ*_i_*) were obtained after fitting the Fourier-filtered data with the sum of decaying sinusoidal functions (see Section S3.3). The highlighted parameters are used in the vibrational analysis. The parameters highlighted in red are reactive modes, and the ones highlighted in violet are assistive modes^16^.

**Figure S20**. The list of assistive or reorganization modes, along with their decay time (shown in the inset). These were obtained by fitting the Fourier-filtered FFT spectrum of the residuals in the time domain (Section S3.3 and Table S2).


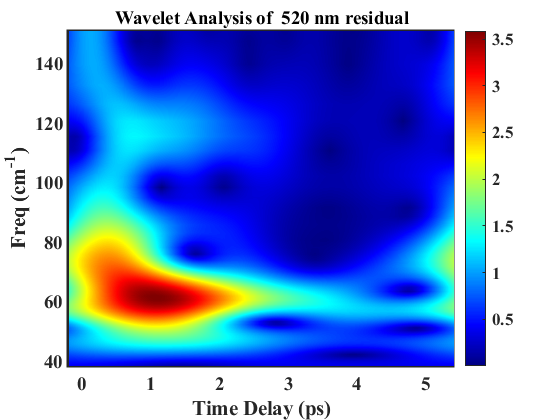


**Figure S21**. Wavelet analysis of the unfiltered residual at 520 nm.

**
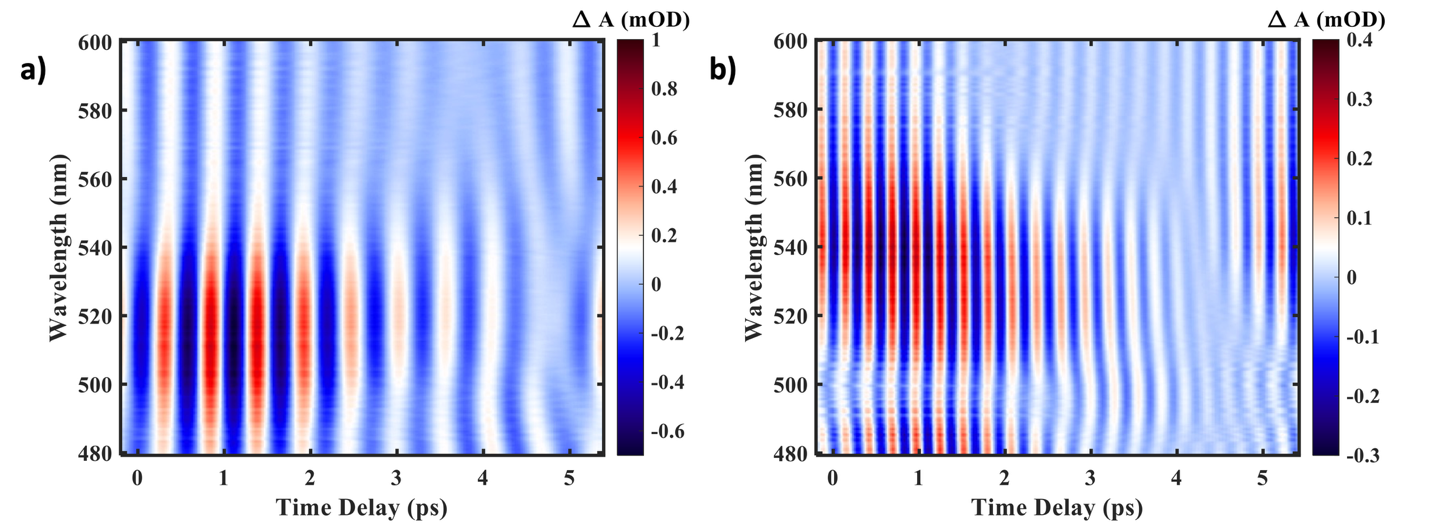
Figure S22**. 2D temporal map of the filtered frequency components at (a) 65 cm^-1^, and (b) 125 cm^-1^, respectively.

**Section S4. Coordinate dependence of anharmonicity and non-linear mixing**

The following equation highlights the series expansion of the spin-orbit Hamiltonian:

$\left\langle\Psi_{D} | \hat{H}_{SO} | \Psi_{H} \right\rangle\left( \vec{Q} \right)=\left\langle\Psi_{D} | \hat{H}_{SO} | \Psi_{H} \right\rangle\left( 0 \right)+ \Sigma_{\alpha}\left. \frac{\partial\left\langle\Psi_{D} | \hat{H}_{SO} | \Psi_{H} \right\rangle}{\partial Q_{\alpha}} \right|_{Q=0}Q_{\alpha}+ \Sigma_{\alpha}\Sigma_{\beta}\left. \frac{\partial^{2}\left\langle\Psi_{D} | \hat{H}_{SO} | \Psi_{H} \right\rangle}{\partial Q_{\alpha}\partial Q_{\beta}} \right|_{Q=0}Q_{\alpha}Q_{\beta}+ \Sigma_{\alpha}\Sigma_{\beta}{\Sigma_{\gamma}\left. \frac{\partial^{3}\left\langle\Psi_{D} | \hat{H}_{SO} | \Psi_{H} \right\rangle}{\partial Q_{\alpha}\partial Q_{\beta}\partial Q_{\gamma}} \right|}_{Q=0}Q_{\alpha}Q_{\beta}Q_{\gamma}+\ldots$

The above equation represents the potential energy expression for the molecular solid, where {*Q*} describes a set of normal coordinates, and {*Q = 0*} means derivatives evaluated at *r_e_*. The third term is the cubic anharmonic correction.

Ψ_D_ = Doublet electronic wavefunction, Ψ_H_ = Sextet electronic wavefunction, Ψ_n_ = Electronic basis set wavefunction, and $\hat{H}_{SO}=$Spin-orbit Hamiltonian, $\sum_{\alpha} \frac{\partial<\Psi_{D}\left| \hat{H}_{SO} \right|\Psi_{H}>}{\partial Q_{\alpha}}Q_{\alpha}$ and $\sum_{\alpha,\beta} \frac{\partial^{2}<\Psi_{D}\left| \hat{H}_{SO} \right|\Psi_{H}>}{\partial Q_{\alpha}\partial Q_{\beta}}Q_{\alpha}Q_{\beta}$ terms depend on the motion along particular coordinates $Q_{\alpha}{and Q}_{\beta}$. For the third term in the above equation, $\sum_{\alpha,\beta} \frac{\partial^{2}<\Psi_{D}\left| \hat{H}_{SO} \right|\Psi_{H}>}{\partial Q_{\alpha}\partial Q_{\beta}}Q_{\alpha}Q_{\beta}$, the coordinates can be written as,$Q_{\alpha}=q_{1}\cos\omega_{1}t, Q_{\beta}$=$q_{2}\cos\omega_{2}t$. This would give rise to the difference frequency and the sum frequency terms, weighed by the $\sum_{\alpha,\beta} \frac{\partial^{2}<\Psi_{D}\left| \hat{H}_{SO} \right|\Psi_{H}>}{\partial Q_{\alpha}\partial Q_{\beta}}$ factor.

Analogous to non-linear optics in bulk crystals, the second-order susceptibility term, χ^2^, determines the mixing of different frequency components^[16,24]^. It is the second-order derivative term in the above equation that determines the vibronic non-linear mixing process, and the efficiency depends on the magnitude of the associated non-linear coefficient. This χ^2^ term is determined by the anharmonicity in the polarizability for non-linear optics. In this regard, the non-linear mixing of vibrational modes is very similar, except that the mixing process occurs in the localized molecular frame of reference. In a similar fashion to non-linear optics, the non-linear coupling between vibrational modes will be strongly influenced by the anharmonicity of the potential energy surface. In this case, the HS potential energy surface ^[24]^. This novel effect is greatly enhanced by the translational symmetry of the crystal. The limited mobility of molecular species in the crystalline domain, along with the absence of solvent bath modes, which inherently wash out the spatial correlations through isotropic ensembles, makes it more favorable to observe the non-linear mixing effects in crystals. The vibrations or assistive modes involved have long enough coherence (sub-picosecond) to observe the non-linear mixing, whereas in solution, these modes would be strongly damped and decay too fast to observe^16^. We presume that the collapse of the wavefunction during the non-linear mixing process occurs in a highly anharmonic component of the reorganizational coordinate. The inherently high anharmonic nature of the collective potential directs the SCO reaction, making the non-linear mixing process highly efficient relative to other nuclear configurations. The fast SCO transition precludes any recurrence towards the initial photoexcited LS state, which conserves the coherent non-linear mixing of vibrational modes induced by this transition (Figure S23).

From ref. 28, we can calculate the temperature-dependent vibrational relaxation lifetime ${}_{\nu}(T)$ of a mode with frequency $\nu$,^[25–28]^

$\left[ \tau_{\upsilon}(T) \right]^{-1}\propto\sum_{\omega_{1}} \sum_{\omega_{2}} \left| {{\hat{H}_{SO}}^{\left( 3 \right)}}_{\nu,\omega_{1},\omega_{2}} \right|^{2} \{\delta\left[ \hbar\left( \nu-\omega_{1}-\omega_{2} \right) \right]\times\left[ n_{\omega_{1}}\left( T \right)+n_{\omega_{2}}\left( T \right)+1 \right]+ \delta\left[ \hbar\left( \nu-\omega_{1}-\omega_{2} \right) \right] [n_{\omega_{1}}\left( T \right)-n_{\omega_{2}}\left( T \right)]$

Where $n_{\omega}$ is the Bose-Einstein distribution function at temperature *T*.

The above equation shows that the cubic anharmonic coupling allows frequency $\nu$ to relax by interaction with a pair of modes $\omega_{1}$and $\omega_{2}$. In the following section 4.1, we derive the non-linear mixing in a classical framework for oscillators 1, 2, and 3. Oscillators 1 and 2 would mix non-linearly to generate oscillator 3.

**Section S4.1. Classical analog of non-linear mixing**

Let $x_{1}(t)$ : Displacement of oscillator 1

Let $x_{2}(t)$ : Displacement of oscillator 2

Let $x_{3}(t)$ : Displacement of oscillator 3 (Difference frequency generation, DFG oscillator)

Equations of motion:

$$\ddot{x}_{1}+2\gamma\dot{x}_{1}+\omega_{1}^{2}x_{1}=0$$

$$\ddot{x}_{2}+2\gamma\dot{x}_{2}+\omega_{2}^{2}x_{2}=0$$

$\ddot{x}_{3}+2\gamma\dot{x}_{3}+\omega_{3}^{2}x_{3}=k x_{1}\left( t \right).x_{2}(t)$

Where,

$\omega_{i}=2\pi c\nu_{i}$ is the angular frequency corresponding to the wavenumber $\nu_{i}$ (cm^-1^).

$\gamma=1/\tau$ is the damping rate (for the simulation $\tau=1$ ps).

$k$ is the non-linear coupling constant or the DFG driving force (for the simulations $k={10}^{28}$)

Figure S24 provides a classical analogue of the non-linear DFG mechanism highlighted in Section S4 and Figure S23. In the molecular frame of reference (quantum world), it is the anharmonicity of the product PES that led to the non-linear DFG mechanism.


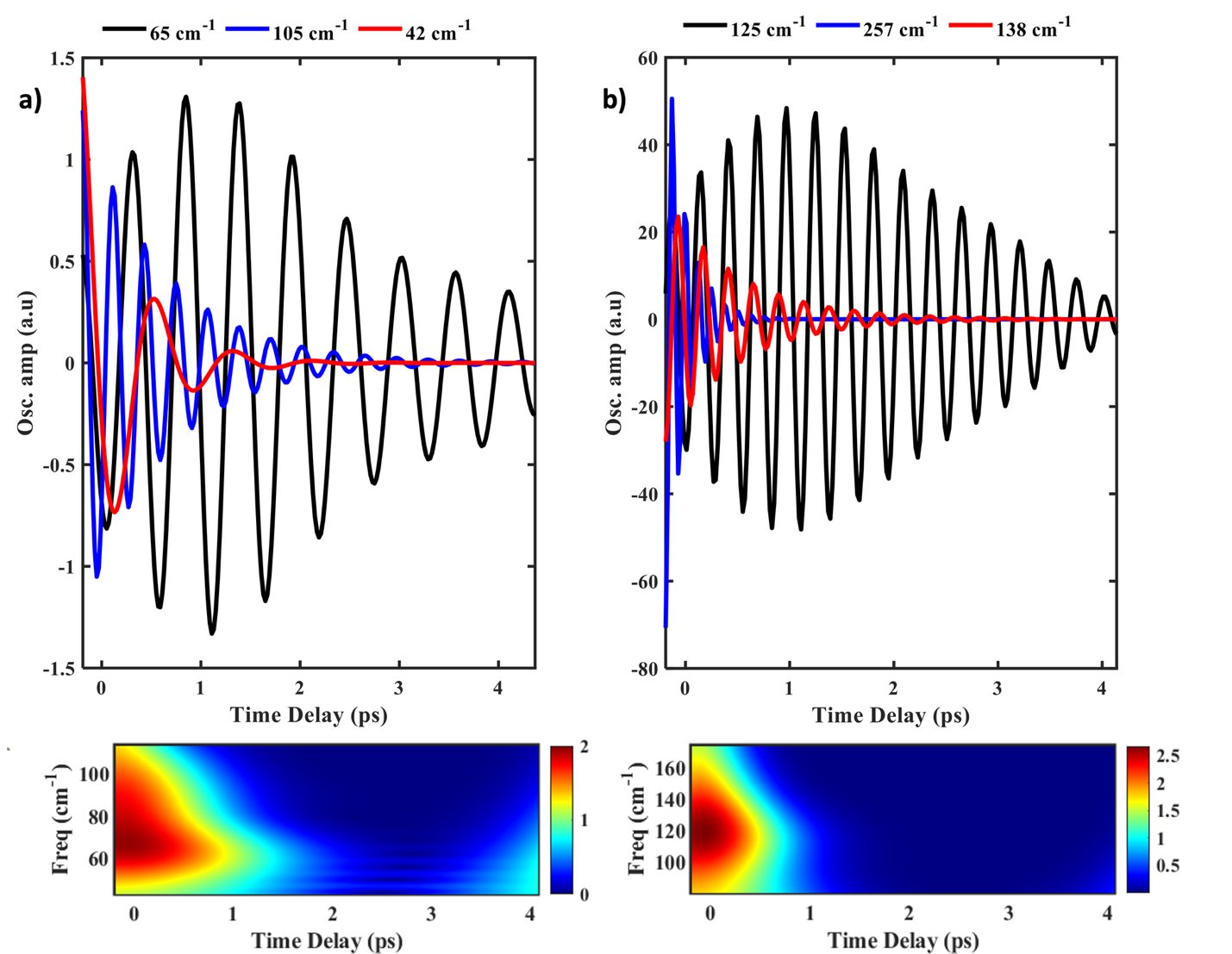


**Figure S23**. (a) and (b): show the coherent build-up of vibrational amplitude, which can be explained as non-linear mixing of assistive modes coherently generating a difference frequency with increasing amplitude (See Figure S24 for the classical analog). The modes implicated are unique combinations within the observed spectral modulation that could coherently drive polarizations at 65 cm^-1^ and 125 cm^-1^, respectively. The bottom panels are the wavelet analysis showing the overall spectral form obtained from the mixing of the assistive modes.

**
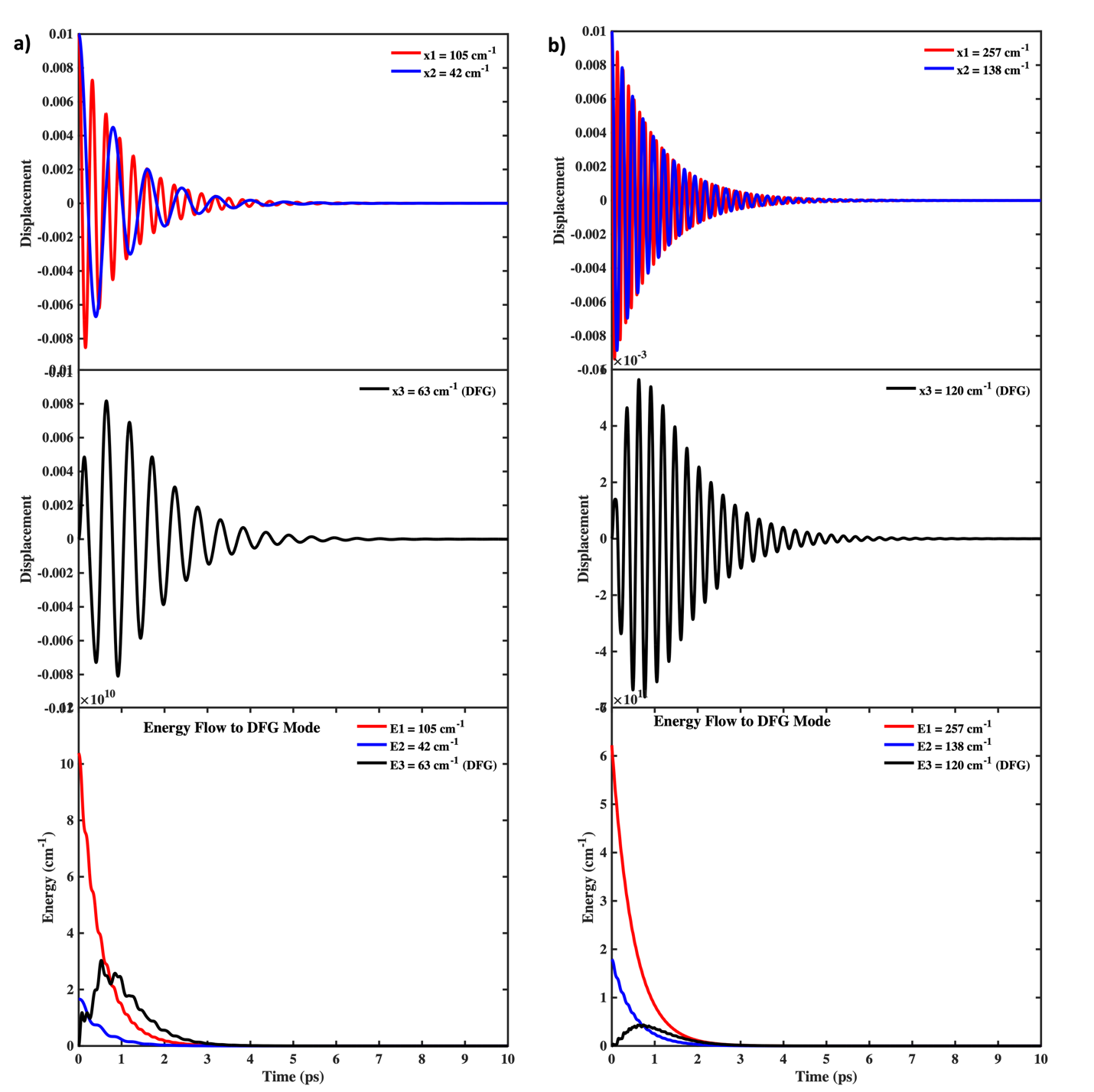
Figure S24**. Upper panel: Shows the decay of the pairs of assistive modes in Fe(III) SCO complex, and the middle panel shows the growth of the (a) 63 cm^-1^ and (b) 120 cm^-1^ mode from the classical analog of non-linear mixing explained in Section S4.1. The lower panel shows the energy flow to the DFG mode (black trace) in the classical oscillator model.


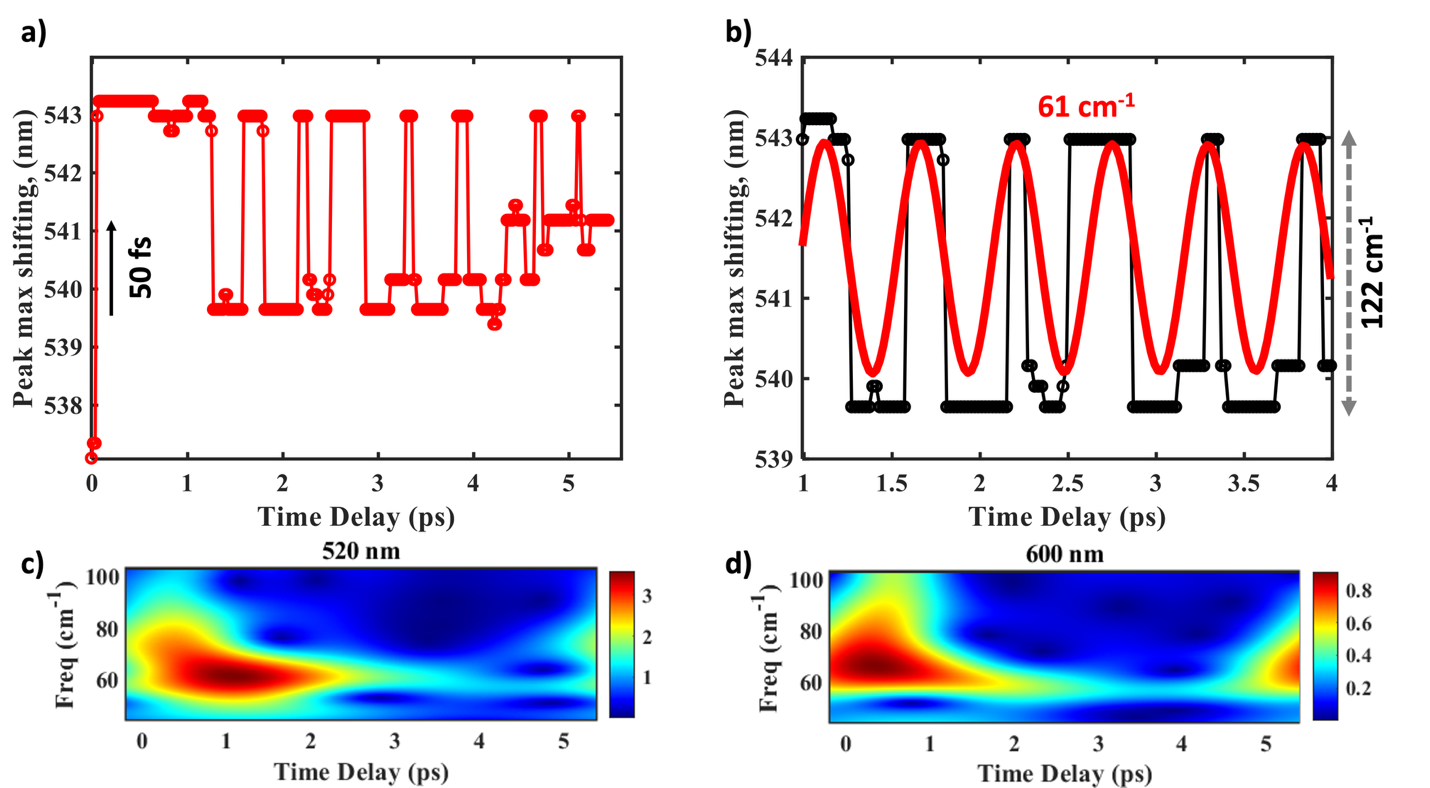


**Figure S25.** (a) Plot of peak maximum shift (ESA feature) versus time delays. (b) Fitting the oscillatory behavior of the peak shift after 1 ps. The sinusoidal fit frequency is 61 cm^-1^, and the energy gap of the modulation is 122 cm^-1^, consistent with vibronic mixing or modulation of the electronic levels (Figure S23). This observation matches the non-impulsive modes with growing amplitude shown in Figure 3d (main manuscript). Wavelet analysis of the unfiltered residual trace shows (c) the growth of the ~65 cm^-1^ mode in the 520 nm probe wavelength region, and (d) the decay of the ~65 cm^-1^ mode in the 600 nm probe wavelength region.

**Section S5. Nonadiabatic dynamics simulations**

The doublet LMCT D_12_ and D_15_ states are predominantly populated at the start of the simulation (see Figure S26). Shortly after excitation, a significant portion of the population is transferred from the low-lying doublet states to the quartet states, which reach approximately 60% of the total population within the first 200 fs. According to the population analysis of natural orbitals from the reference diabatic states, the quartet states actively populated during the dynamics exhibit an open-shell configuration, with unpaired electrons localized on either the ligand or metal orbitals and one electron occupying the e_g_ orbitals. The sextet states (red line) display a slightly slower rise time than the quartet states, reaching approximately 90% of the total excited population at the end of the first picosecond and maintaining this value until the end of the simulation. The sextet states, actively populated during the dynamics, exhibit an open-shell configuration, as revealed by the population analysis of natural orbitals from the reference diabatic states, with unpaired electrons localized on the metal 3d orbitals and two electrons occupying the e_g_ orbitals. The quartet state population peaks at around 200 fs after excitation but diminishes to less than 10% by the end of the simulation. The doublet state population becomes even smaller over time, eventually becoming almost negligible.


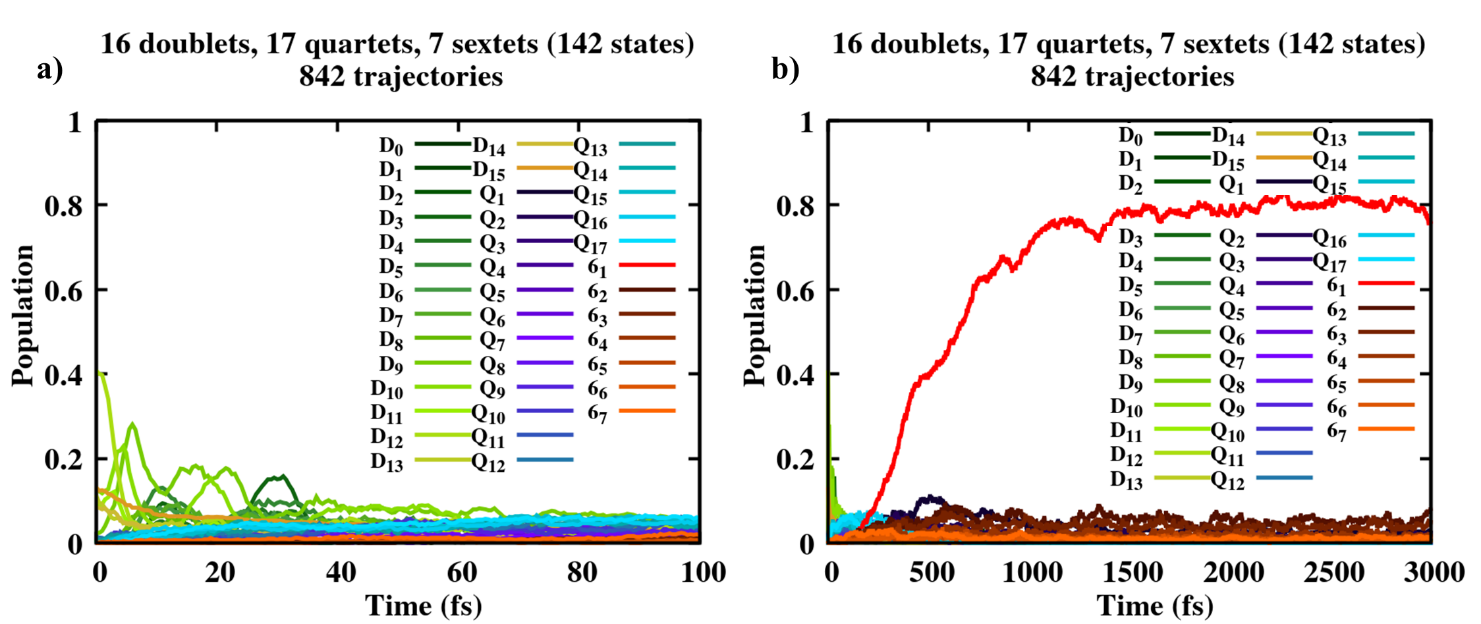


**Figure. S26**. Time-resolved electronic populations obtained from non-adiabatic dynamics simulations and averaged over 842 trajectories. The blue lines represent the populations of doublet states, the green lines correspond to the populations of quartet states, and the red line corresponds to the populations of sextet states. (a) From 0 to 100 fs, (b) from 0 to 3 ps.

**
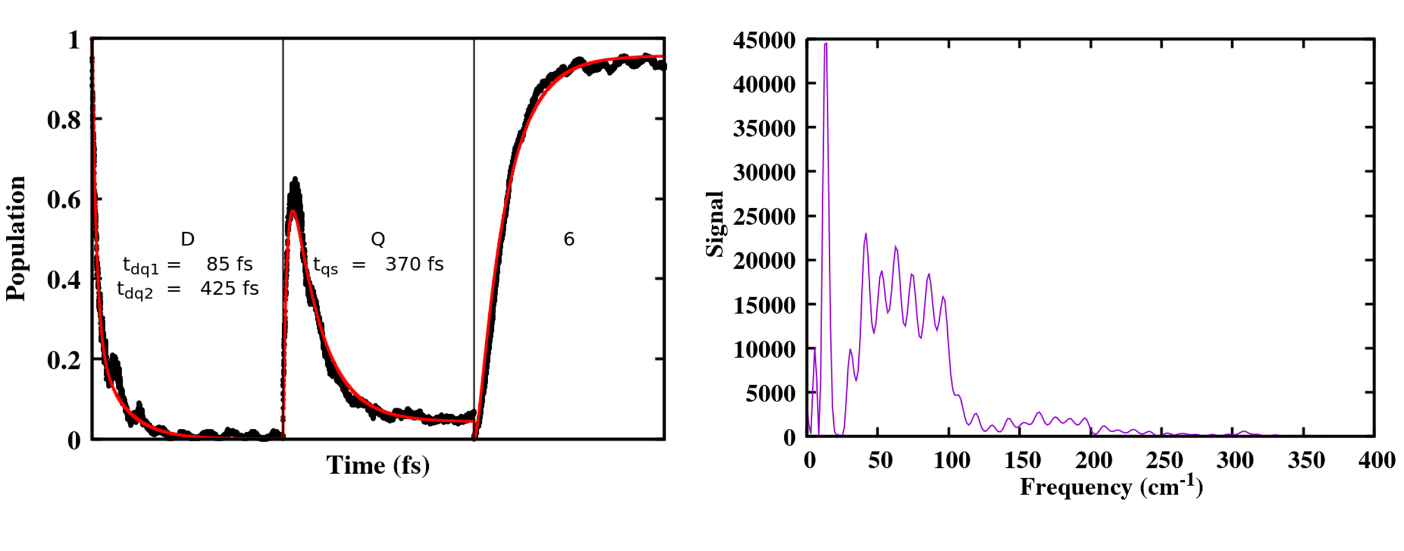
** **Figure S27**. (a) Kinetic fit of the time-resolved electronic populations of the summed doublet (D), quartet (Q), and sextet (6) states. The initial D → Q intersystem crossing is modeled as a bi-exponential decay with time constants τ_dq1_ = 85 fs (75% amplitude) and τ_dq2_ = 425 fs (25% amplitude), indicating two distinct timescales for the depopulation of the doublet state. It is assumed that, at time zero, only the doublet states are populated due to δ-pulse excitation. (b) Fourier transform spectra of the time-resolved electronic populations of the quartet states. The Fourier transform analysis reveals several frequencies, primarily in the low-frequency region, with main peaks identified at 23 cm⁻¹, 40 cm⁻¹, 52 cm⁻¹, 75 cm⁻¹, 96 cm⁻¹, and 108 cm⁻¹.

**
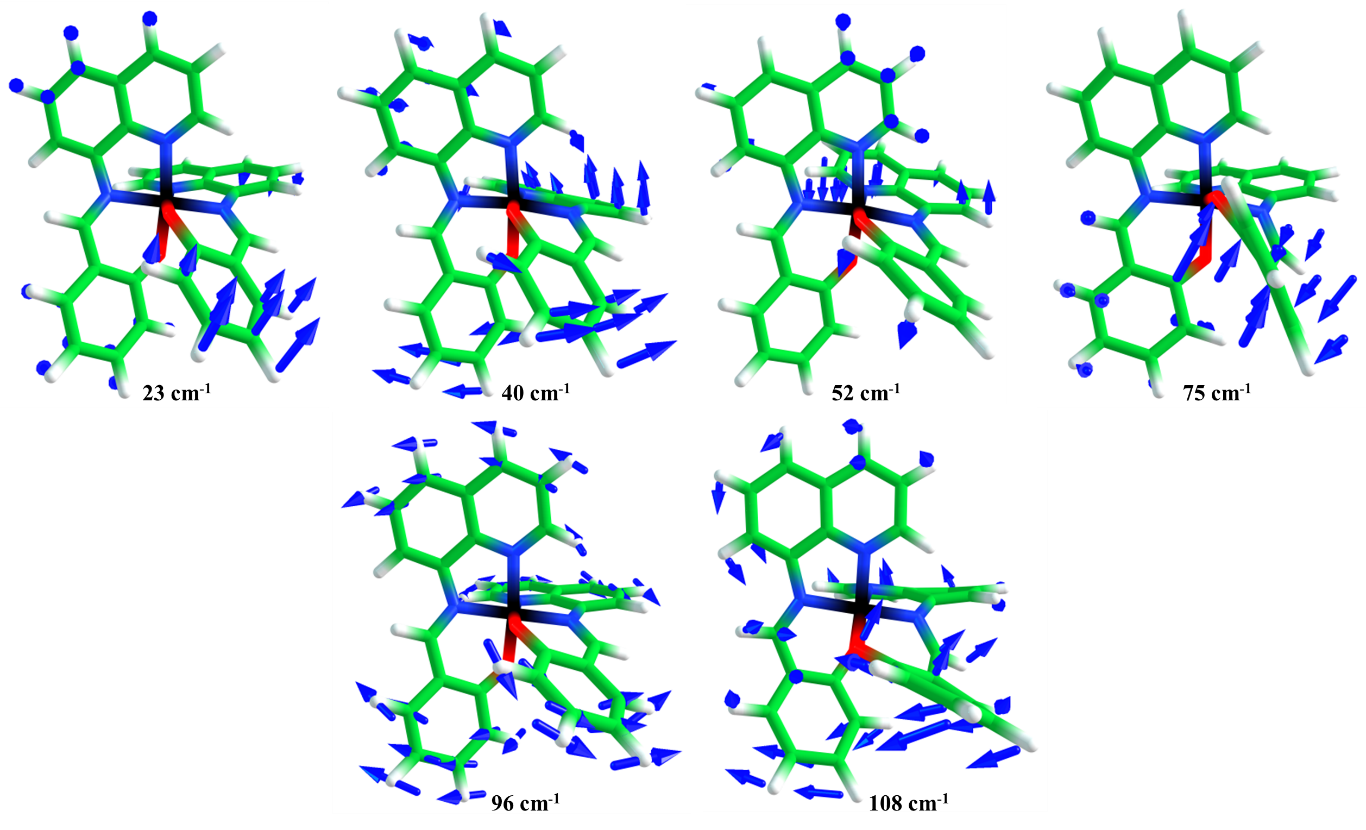
**

**Figure S28**. Normal mode motions identified from the Fourier transform of the time-resolved quartet population.

**Section S6. Normal modes from simulated transient absorption spectr**
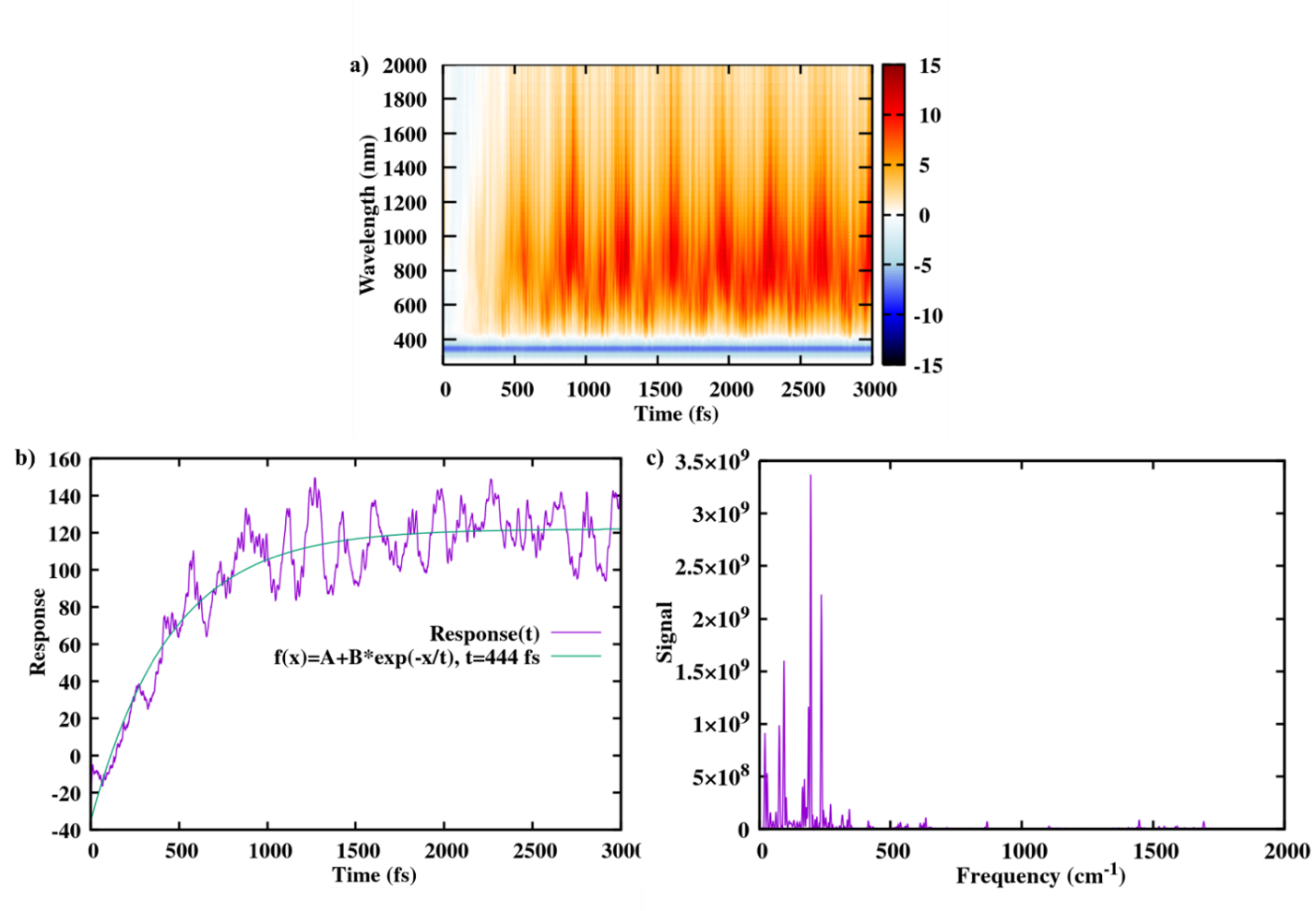
**um**

**Figure S29**. Simulated (a) transient absorption (TA) spectrum, (b) exponential fit of the modulation in the TA signal, and (c) Fourier transform of the oscillatory signal. The simulated TA exhibits a positive signal above 480 nm and a negative signal below this wavelength. Strong oscillatory modulation in the TA signal indicates coherent nuclear wave packet motion. Kinetic fitting of the signal yielded a time constant of 444 fs. The Fourier transform analysis of the oscillatory component reveals frequency components at 23 cm⁻¹, 40 cm⁻¹, 52 cm⁻¹, 75 cm⁻¹, 96 cm⁻¹, 108 cm⁻¹, 164 cm⁻¹, 196 cm⁻¹, and 238 cm⁻¹, which closely match experimentally observed vibrational modes (See Table 1, main manuscript).


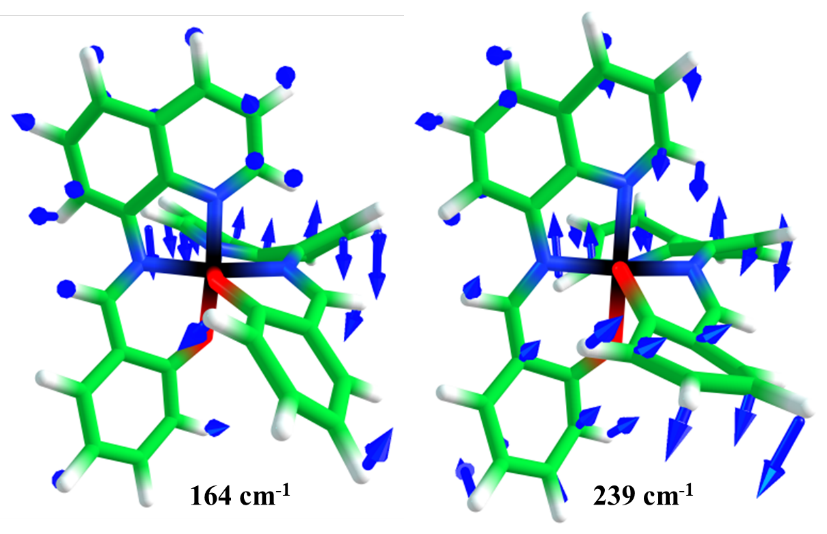


**Figure S30**. Normal mode motions identified from the Fourier transform of the simulated TA spectrum.

| **Calculated Frequency (cm^-1^)** | **Experimental Frequency (cm^-1^)** |
| --- | --- |
| 23 | 23 |
| 40 | 42 |
| 52 | 53 |
| 75 | 84 |
| 96 | 96 |
| 108 | 104 |
| 164 | 160 |
| 196 (v_max_) | 194 |
| 238 (v_max2_) | 236 |

**Table S3**. List of calculated frequencies from Figure S29 and experimental frequencies observed in the experimental TA data.

**Section S7. Coherence analysis**

The approach for coherence mode analysis was taken from Refs. ^[29,30]^. The nonadiabatic dynamics simulations were performed in Cartesian coordinates, while the subsequent coherence analysis was carried out in the harmonic normal mode representation. The transformation from Cartesian to normal mode coordinates was performed using a mass-weighted approach. The resulting $Q_{i}$ are thus mass- and frequency-scaled, dimensionless displacements, defined like:

$$Q_{i}=\sqrt{\frac{\omega_{i}}{\hbar}}\sum_{A} K_{Ai}\sqrt{M_{A}}\left( r_{A}-r_{A}^{\mathrm{ref}} \right)$$

where ω_i_ is the frequency of normal mode i, M_A_ is an atomic mass, and K_Ai_ is the conversion matrix between mass-weighted Cartesian and normal coordinates, the reference geometry corresponds to 𝑄 = 0.

To identify the extent of coherent motion, we compute two measures for each mode:

**Section S7.1. Total standard deviation**
The total standard deviation for the mode *m* accounts for fluctuations over all trajectories and time steps:

$$\sigma_{\mathrm{tot}}^{\left( m \right)}=\sqrt{\frac{1}{NT}\sum_{i=1}^{N} \sum_{t} \left( Q_{i}^{\left( m \right)}\left( t \right)-\left\langle Q^{\left( m \right)} \right\rangle\right)^{2}}$$

where N is the number of trajectories, T the total number of time steps, t is the time, and

$$\left\langle Q^{\left( m \right)} \right\rangle=\frac{1}{NT}\sum_{i,t} Q_{i}^{\left( m \right)}\left( t \right)$$

is the ensemble average for mode *m*.

**Section S7.2. Coherent standard deviation**
The coherent component is obtained by first computing the time-dependent average trajectory for each mode:

$$\bar{Q}^{(m)}(t)=\frac{1}{N}\sum_{i=1}^{N} Q_{i}^{(m)}(t)$$

The standard deviation of the average trajectory (i.e., the coherent standard deviation) is then given by

$$\sigma_{\mathrm{coh}}^{(m)}=\sqrt{\frac{1}{T}\sum_{t} \left( \bar{Q}^{(m)}(t)-\left\langle\bar{Q}^{(m)} \right\rangle_{t} \right)^{2}}$$

with

$$\left\langle\bar{Q}^{(m)} \right\rangle_{t}=\frac{1}{T}\sum_{t} \bar{Q}^{(m)}(t)$$

The ratio between $\sigma_{\mathrm{coh}}^{(m)}$ and $\sigma_{\mathrm{tot}}^{\left( m \right)}$ serves as an indicator of the coherence in the *m*-th mode. A high value of $\sigma_{\mathrm{coh}}^{(m)}/\sigma_{\mathrm{tot}}^{(m)}$ implies that the nuclear motion along that mode is largely in-phase (coherent) across the ensemble of trajectories, whereas a low value indicates that random (incoherent) fluctuations dominate.


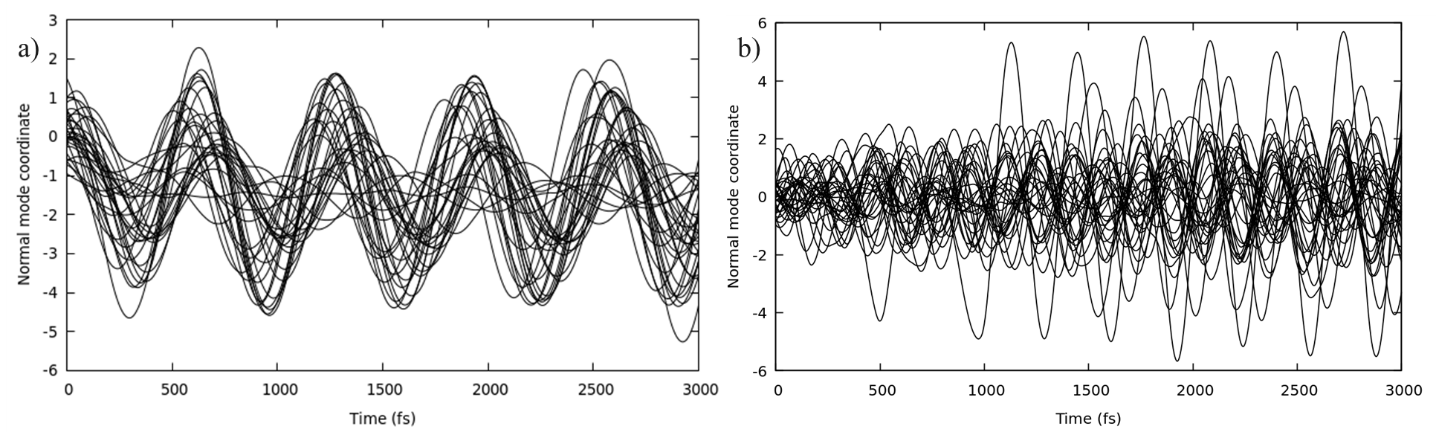
 **Figure S31**. Comparison of normal mode behavior: (a) Coherent motion exhibiting synchronous, in-phase oscillations across trajectories and (b) random motion whose fluctuations cancel in the averaged trajectory.

This procedure, adapted from Refs. 29 and 30, allows us to identify which normal modes exhibit significant and coherent nuclear displacements. Such modes are presumed to be closely associated with the excitation process and the subsequent ultrafast dynamics.
Using described above analysis we identified the normal modes with frequencies 23 cm⁻¹, 40 cm⁻¹, 52 cm⁻¹, 80 cm⁻¹, 96 cm⁻¹, 164 cm⁻¹, 201 cm⁻¹, 229 cm⁻¹, 242 cm⁻¹, 257 cm⁻¹, 273 cm⁻¹, 1720 cm⁻¹.


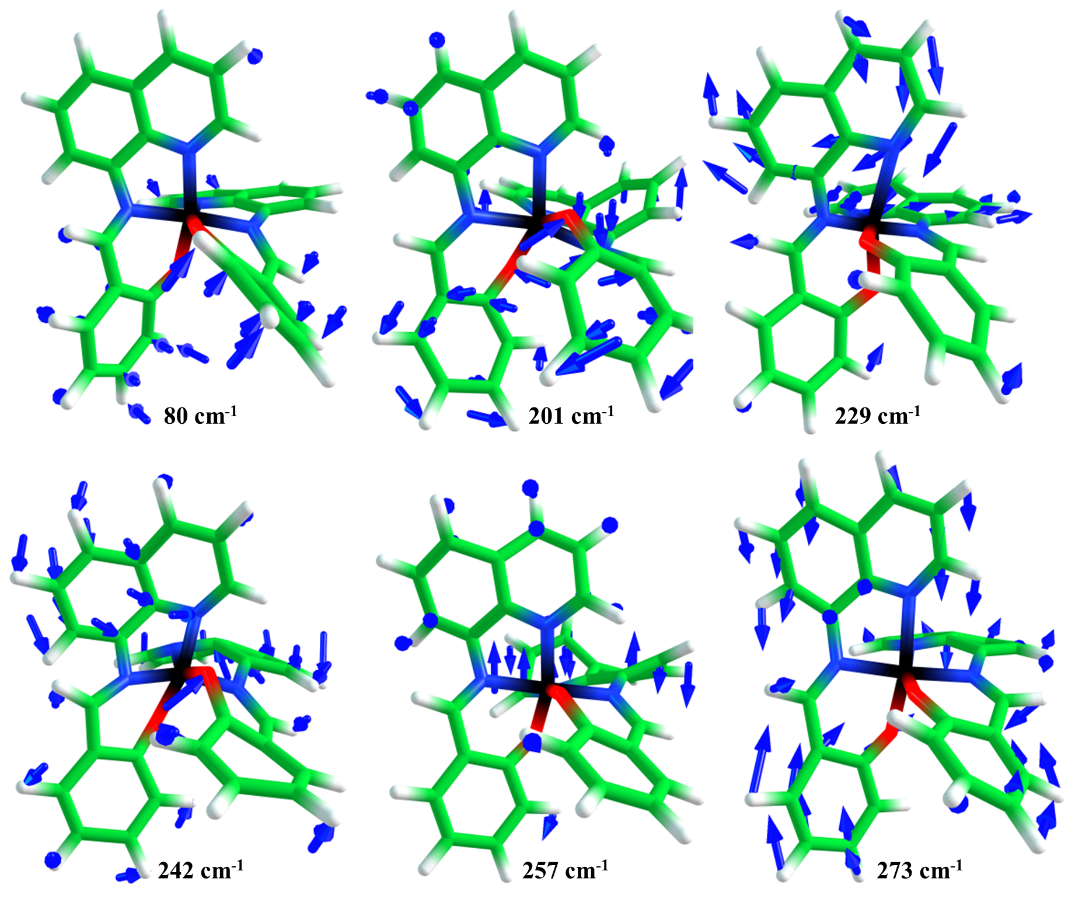


**Figure S32**. Normal mode motions identified from the coherence analysis

**Section S8. Bond length analysis**


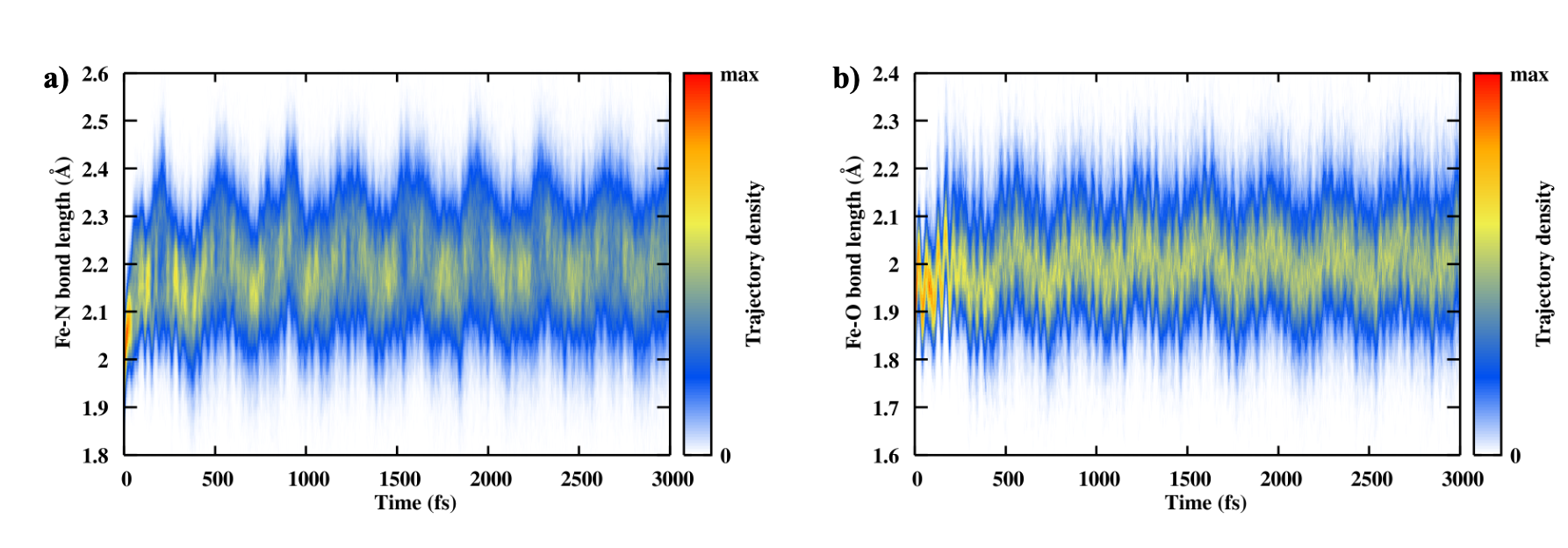


**Figure S33**. Temporal evolution of the (a) Fe–N and (b) Fe–O bond length distributions for the simulated [Fe(qsal)_2_]^2+^ cation following photoexcitation. The heatmaps represent the trajectory density obtained from 842 non-adiabatic dynamics trajectories (convolved with a Gaussian FWHM of 0.01 Å). The prominent red regions track the center of the nuclear wavepacket, highlighting the rapid structural expansion and subsequent coherent oscillations associated with the high-spin state.

Figure S33 illustrates the structural reorganization of the primary coordination sphere during the spin-crossover process. At time zero, the density is concentrated in a narrow region corresponding to the LS equilibrium geometry (Fe–N ≈ 1.96 Å; Fe–O ≈ 1.88 Å). Immediately following excitation, this density streak shifts vertically to longer bond lengths within the first ~100 fs. This rapid displacement is consistent with the fast kinetic component (τ =85 fs) derived from the population dynamics. After a while, the system settles into a new oscillating quasi-equilibrium centered at significantly elongated bond lengths: Fe–N expands to approximately 2.20 Å (an increase of ~0.24 Å) and Fe–O to approximately 2.00 Å (an increase of ~0.12 Å). This elongation is the direct structural signature of the High-Spin (HS) state, resulting from the population of the antibonding e_g_ orbitals.

The trajectory density does not simply relax into a static distribution, but exhibits pronounced, long-lived oscillations (visible as the "wavy" high-density red/yellow regions) that persist throughout the 3 ps simulation window. The expansions and contractions of the Fe–N and Fe–O bonds occur in phase, which confirms the activation of the symmetric breathing mode. The oscillation period observed in the heatmaps is around 200 fs, which corresponds to a vibrational frequency of ∼160 cm⁻¹. The persistence of these coherent oscillations suggests that the secondary kinetic step (quartet to sextet) occurs while the molecule is still undergoing significant vibrational motion. This implies that the transition to the final sextet state is likely modulated by this metal-ligand stretching motion, which periodically brings the geometry into the vicinity of the quartet-sextet crossing seam.

**Section S9. References**

[1] D. Escudero, L. González, “RASPT2/RASSCF vs Range-Separated/Hybrid DFT Methods: Assessing the Excited States of a Ru(II)bipyridyl Complex” *J. Chem. Theory Comput.* 2012, *8*, 203–213.

[2] F. Aquilante, R. Lindh, T. Bondo Pedersen, “Unbiased auxiliary basis sets for accurate two-electron integral approximations” *J. Chem. Phys.* 2007, *127*, 114107.

[3] B. O. Roos, R. Lindh, P.-Å. Malmqvist, V. Veryazov, P.-O. Widmark, “New Relativistic ANO Basis Sets for Transition Metal Atoms” *J. Phys. Chem. A.* 2005, *109*, 6575–6579.

[4] B. O. Roos, R. Lindh, P.-Å. Malmqvist, V. Veryazov, P.-O. Widmark, “Main Group Atoms and Dimers Studied with a New Relativistic ANO Basis Set” *J. Phys. Chem. A.* 2004, *108*, 2851–2858.

[5] K. Andersson, B. O. Roos, “Excitation energies in the nickel atom studied with the complete active space SCF method and second-order perturbation theory” *Chem. Phys. Lett.* 1992, *191*, 507–514.

[6] K. Pierloot, S. Vancoillie, “Relative energy of the high-(^5^T_2g_) and low-(^1^A_1g_) spin states of [Fe(H_2_O)_6_]^2+^, [Fe(NH_3_)_6_]^2+^, and [Fe(bpy)_3_]^2+^: CASPT2 versus density functional theory” *J. Chem. Phys.* 2006, *125*, 124303.

[7] F. Plasser, A. I. Krylov, A. Dreuw, “libwfa: Wavefunction analysis tools for excited and open‐shell electronic states” *WIREs Comput. Mol. Sci.*  2022, *12*, e1595.

[8] R. Field, L. C. Liu, W. Gawelda, C. Lu, R. J. D. Miller, “Spectral Signatures of Ultrafast Spin Crossover in Single Crystal [Fe^II^(bpy)_3_](PF_6_)_2_” *Eur. J. Inorg. Chem.*  2016, *22*, 5118–5122.

[9] Y. Jiang, S. Hayes, S. Bittmann, A. Sarracini, L. C. Liu, H. M. Müller-Werkmeister, A. Miyawaki, M. Hada, S. Nakano, R. Takahashi, S. Banu, S. Koshihara, K. Takahashi, T. Ishikawa, R. J. D. Miller, “Direct observation of photoinduced sequential spin transition in a halogen-bonded hybrid system by complementary ultrafast optical and electron probes” *Nat. Commun.* 2024, *15*, 4604.

[10] K. M. Siddiqui, G. Corthey, S. A. Hayes, A. Rossos, D. S. Badali, R. Xian, R. S. Murphy, B. J. Whitaker, R. J. D. Miller, “Synchronised Photoreversion of Spirooxazine Ring Opening in Thin Crystals to Uncover Ultrafast Dynamics” *Cryst. Eng. Comm.* 2016, *18*, 7212.

[11] R. J. D. Miller, O. Paré-Labrosse, A. Sarracini, J. E. Besaw, “Three-dimensional view of ultrafast dynamics in photoexcited bacteriorhodopsin in the multiphoton regime and biological relevance” *Nat. Commun.* 2020, *11*, 1240.

[12] K. Takahashi, K. Yamamoto, T. Yamamoto, Y. Einaga, Y. Shiota, K. Yoshizawa, H. Mori, “High-Temperature Cooperative Spin Crossover Transitions and Single-Crystal Reflection Spectra of [Fe^III^(qsal)_2_](CH_3_OSO_3_) and Related Compounds” *Crystals (Basel).* 2019, *9*, 81.

[13] L. C. Liu, *Chemistry in Action: Making Molecular Movies with Ultrafast Electron Diffraction and Data Science*, Springer International Publishing, 2020.

[14] I. H. M. van Stokkum, D. S. Larsen, R. van Grondelle, “Global and target analysis of time-resolved spectra” *Biochim. Biophys. Acta. – Bioenergetics.* 2004, *1657*, 82–104.

[15] S. F. Bittmann, R. Dsouza, K. M. Siddiqui, S. A. Hayes, A. Rossos, G. Corthey, M. Kochman, V. I. Prokhorenko, R. S. Murphy, H. Schwoerer, R. J. D. Miller, “Ultrafast ring-opening and solvent-dependent product relaxation of photochromic spironaphthopyran” *Phys. Chem. Chem. Phys.* 2019, *21*, 18119–18127.

[16] S. Mitra, M. Zhang, S. F. Bittmann, J. Cai, X. Dong, R. S. Murphy, Z. Li, R. J. D. Miller, “Elucidating the reaction kernel and probing the effect of anharmonicity in the ring-closing reaction of fulgide single crystals” *Chem. Sci.* 2025, *16*, 19118–19129.

[17] R. Bertoni, M. Lorenc, H. Cailleau, A. Tissot, J. Laisney, M. L. Boillot, L. Stoleriu, A. Stancu, C. Enachescu, E. Collet, “Elastically driven cooperative response of a molecular material impacted by a laser pulse” *Nat. Mater.* 2016, *15*, 606–610.

[18] A. Marino, M. Cammarata, S. F. Matar, J.-F. Létard, G. Chastanet, M. Chollet, J. M. Glownia, H. T. Lemke, E. Collet, “Activation of coherent lattice phonon following ultrafast molecular spin-state photo-switching: A molecule-to-lattice energy transfer” *Struct. Dyn.* 2016, *3*, 023605.

[19] D. Vinci, K. Ridier, F. Qi, F. Ardana-Lamas, P. Zalden, L. C. Liu, T. Eklund, M. S. Jakobsen, R. Schubert, D. Khakhulin, C. Deiter, N. Bottin, H. Yousef, D. von Stetten, P. Łaski, R. Kamiński, K. N. Jarzembska, R. F. Wallick, T. Stensitzki, R. M. van der Veen, H. M. Müller-Werkmeister, G. Molnár, D. Xiang, C. Milne, M. Lorenc, Y. Jiang, “Capturing ultrafast molecular motions and lattice dynamics in spin crossover film using femtosecond diffraction methods” *Nat. Commun.* 2025, *16*, 2043.

[20] S. Mitra, S. R. K. Ainavarapu, J. Dasgupta, “Long-Range Charge Delocalization Mediates the Ultrafast Ligand-to-Metal Charge Transfer Dynamics at the Cu^2+^ -Active Site in Azurin” *J. Phys. Chem. B.* 2022, *126*, 5390–5399.

[21] M. Liebel, C. Schnedermann, T. Wende, P. Kukura, “Principles and Applications of Broadband Impulsive Vibrational Spectroscopy” *J. Phys. Chem. A.* 2015, *119*, 9506–9517.

[22] P. J. M. Johnson, A. Halpin, T. Morizumi, V. I. Prokhorenko, O. P. Ernst, R. J. D. Miller, “Local vibrational coherences drive the primary photochemistry of vision” *Nat. Chem.* 2015, *7*, 980–986.

[23] R. Xian, G. Corthey, D. M. Rogers, C. A. Morrison, V. I. Prokhorenko, S. A. Hayes, R. J. D. Miller, “Coherent ultrafast lattice-directed reaction dynamics of triiodide anion photodissociation” *Nat. Chem.* 2017, *9*, 516–522.

[24] R. W. Boyd in *Nonlinear Optics (Third Edition)* (Ed.: R.W. Boyd), Academic Press, Burlington, 2008, pp. 253–275.

[25] D. D. Dlott, “Vibrational cooling (and heating) of large molecules in solids” *J. Lumin*.1990, *45*, 397–400.

[26] J. R. Hill, D. D. Dlott, “Theory of vibrational cooling in molecular crystals: Application to crystalline naphthalene” *J. Chem. Phys.* 1988, *89*, 842–858.

[27] J. R. Hill, D. D. Dlott, “A model for ultrafast vibrational cooling in molecular crystals” *J. Chem. Phys.* 1988, *89*, 830–841.

[28] H. Kim, D. D. Dlott, “Theory of ultrahot molecular solids: Vibrational cooling and shock-induced multiphonon up pumping in crystalline naphthalene” *J. Chem. Phys.* 1990, *93*, 1695–1709.

[29] S. Mai, L. González, “Identification of important normal modes in nonadiabatic dynamics simulations by coherence, correlation, and frequency analyses” *J. Chem. Phys.* 2019, *151*, 244115.

[30] F. Plasser, M. Barbatti, A. J. A. Aquino, H. Lischka, “Excited-State Diproton Transfer in [2,2′-Bipyridyl]-3,3′-diol: the Mechanism Is Sequential, Not Concerted” *J. Phys. Chem. A.* 2009, *113*, 8490–8499.
